# Exposure Versus Cognitive Restructuring Techniques in Brief Internet-Based Cognitive Behavioral Treatment for Arabic-Speaking People With Posttraumatic Stress Disorder: Randomized Clinical Trial

**DOI:** 10.2196/48689

**Published:** 2023-12-13

**Authors:** Jana Stein, Max Vöhringer, Birgit Wagner, Nadine Stammel, Yuriy Nesterko, Maria Böttche, Christine Knaevelsrud

**Affiliations:** 1 Clinical Psychological Intervention Department of Education and Psychology Freie Universität Berlin Berlin Germany; 2 Department for Transcultural and Traumatic Stress Studies Center ÜBERLEBEN Berlin Germany; 3 Clinical Psychology and Psychotherapy Medical School Berlin Berlin Germany; 4 Department of Medical Psychology and Medical Sociology University of Leipzig Leipzig Germany

**Keywords:** posttraumatic stress, posttraumatic stress disorder, PTSD, Middle East, North Africa, Arabic, Arabic-speaking people, internet, internet-based, exposure, cognitive restructuring, randomized clinical trial, RCT, cognitive behavioral therapy, CBT, cognitive behavioral treatment

## Abstract

**Background:**

Cognitive behavioral interventions delivered via the internet are demonstrably efficacious treatment options for posttraumatic stress disorder (PTSD) in underserved, Arabic-speaking populations. However, the role of specific treatment components remains unclear, particularly in conflict-affected areas of the Middle East and North Africa.

**Objective:**

This study aims to evaluate 2 brief internet-based treatments in terms of efficacy, including change in PTSD symptom severity during treatment. Both treatments were developed in line with Interapy, an internet-based, therapist-assisted cognitive behavioral therapy protocol for PTSD and adapted to the specific research question. The first treatment comprised self-confrontation and social sharing (exposure treatment; 6 sessions); the second comprised cognitive restructuring and social sharing (cognitive restructuring treatment; 6 sessions). The 2 treatments were compared with each other and with a waitlist control group.

**Methods:**

In total, 365 Arabic-speaking participants from the Middle East and North Africa (mean age 25.49, SD 6.68 y) with PTSD were allocated to cognitive restructuring treatment (n=118, 32.3%), exposure treatment (n=122, 33.4%), or a waitlist control group (n=125, 34.2%) between February 2021 and December 2022. PTSD symptom severity, posttraumatic maladaptive cognitions, anxiety, depressive and somatoform symptom severity, and quality of life were assessed via self-report at baseline and after treatment or waiting time. PTSD symptom severity was also measured throughout treatment or waiting time. Treatment satisfaction was assessed after treatment completion. Treatment use and satisfaction were compared between the 2 treatment conditions using appropriate statistical tests (eg, chi-square and Welch tests). Multiple imputation was performed to address missing data and evaluate treatment-associated changes. These changes were analyzed using multigroup change modeling in the completer and intention-to-treat samples.

**Results:**

Overall, 200 (N=240, 83.3%) participants started any of the treatments, of whom 123 (61.5%) completed the treatment. Treatment condition was not significantly associated with the proportion of participants who started versus did not start treatment (*P*=.20) or with treatment completion versus treatment dropout (*P*=.71). High treatment satisfaction was reported, with no significant differences between the treatment conditions (*P*=.48). In both treatment conditions, PTSD, anxiety, depressive and somatoform symptom severity, and posttraumatic maladaptive cognitions decreased, and quality of life improved significantly from baseline to the posttreatment time point (*P*≤.001 in all cases). Compared with the baseline assessment, overall PTSD symptom severity decreased significantly after 4 sessions in both treatment conditions (*P*<.001). Moreover, both treatment conditions were significantly superior to the waitlist control group regarding overall PTSD symptom severity (*P*<.001) and most other comorbid mental health symptoms (*P*<.001 to *P*=.03). Differences between the 2 conditions in the magnitude of change for all outcome measures were nonsignificant.

**Conclusions:**

Internet-based cognitive behavioral treatments for PTSD focusing primarily on either self-confrontation or cognitive restructuring are applicable and efficacious for Arabic-speaking participants.

**Trial Registration:**

German Clinical Trials Register DRKS00010245; https://drks.de/search/de/trial/DRKS00010245

## Introduction

### Background

The Middle East and North Africa region is both geographically and culturally diverse. Ongoing civil wars, local conflicts, political instability, economic insecurity (ie, high unemployment rates), and high levels of displacement [1,2] have taken a toll on the mental health of the civilian population in this region [3,4]. A meta-analysis of prevalence rates of mental disorders in the Eastern Mediterranean region identified depression, generalized anxiety disorder, and posttraumatic stress disorder (PTSD) as the most prevalent disorders, with pooled current prevalence rates of 20.5%, 10.3%, and 9.5%, respectively [5]. However, despite the high prevalence of mental disorders, many individuals do not receive adequate treatment [6,7], partly because of the dearth of available professionals in the region. For instance, the World Health Organization [8] reported that only 0.7 psychologists per 100,000 population serve the Eastern Mediterranean region compared with 5.4 psychologists in the Americas.

Interventions provided via the internet may offer a solution to bridge the gap between the high demand for mental health services and the limited access to such support. By combining the advantage of high availability with the independence of the therapist’s location, internet-based interventions represent a promising opportunity to provide support in regions with low access to psychotherapeutic help (eg, in regions shattered by conflict [9]). Moreover, the easy accessibility and greater visual anonymity of internet-based interventions often facilitate their use by people who have been exposed to highly stigmatizing traumatic events, who may fear prejudgment when seeking help for mental health problems, or when mobility is limited (ie, women not being permitted to leave the house without a male attendant). A survey of 503 Arabic-speaking people found that 73% were willing to try an intervention for anxiety and depression delivered via the internet [10]. Furthermore, in a previous study, >6000 Arabic-speaking people completed a screening process to participate in internet-based interventions for the treatment of depression or PTSD [11], suggesting that Arabic-speaking people have a strong interest in psychological treatments delivered via the internet.

In addition, several meta-analyses have pointed to the efficacy of internet-delivered cognitive behavioral interventions for the treatment of PTSD [12]. Cognitive behavioral interventions delivered through the internet seem to be superior to inactive control groups [12] and noninferior to cognitive behavioral treatments delivered face-to-face [13]. A meta-analysis focusing specifically on the efficacy of cognitive behavioral therapy for Arabic-speaking people with PTSD, anxiety, or depression found large effect sizes (ie, PTSD: *g*=2.08; depression: *g*=1.26; anxiety: *g*=1.44), and a reduction in psychopathological symptoms was reported for all included internet-based cognitive behavioral interventions (n=5 of 9 studies) [14]. Knaevelsrud et al [15] similarly found high levels of satisfaction with an internet-based trauma-focused cognitive behavioral intervention among traumatized Arabic-speaking people. Thus, cognitive behavioral interventions delivered via the internet appear to be accepted and significantly reduce distressing symptoms in different populations, including Arabic-speaking people with PTSD [11,15].

Although trauma-focused cognitive behavioral approaches are superior to cognitive behavioral approaches without a trauma focus [16] and are, therefore, the treatment of first choice for adults with a diagnosis of PTSD [17], and trauma-focused cognitive behavioral approaches delivered via the internet show promising results, it is hugely important to examine the differential effects of specific cognitive behavioral treatment components—particularly when delivered via the internet—to provide the best possible care for individuals with PTSD. In the face-to-face setting, a number of studies have investigated the specific effect of cognitive methods (ie, cognitive restructuring [CR]) on PTSD symptoms compared with exposure-based methods [18-20], but the superiority of exposure methods, cognitive methods, or a combination of the 2 could not be clearly demonstrated [20-22]. Studies on the efficacy of internet-delivered psychotherapeutic interventions with a specific focus on either exposure-based techniques [11] or cognitive methods [23,24] have revealed significant improvements in PTSD and comorbid mental health symptoms. Although exposure-based techniques delivered via the internet have been successfully implemented for Arabic-speaking people with PTSD (overall PTSD symptom improvement during treatment: *d*=1.13) [11], exposure might not be a suitable treatment option for all individuals with PTSD as some may be unwilling to confront the traumatic event in detail and may drop out of the intervention before treatment gains become apparent. In particular, for people in Arabic-speaking cultures who have experienced any form of sexual violence, going through the traumatic event in detail may be a huge burden as this type of trauma is likely to be associated with great shame, loss of honor, or feelings of guilt [25]. For internet-delivered interventions combining exposure and cognitive methods for Arabic-speaking people with PTSD, dropout rates of approximately 37% have been reported [26]. Similar dropout rates were found when only providing exposure treatment for this population [11], highlighting the need for additional treatment options without a focus on exposure.

In summary, studies conducted in face-to-face psychotherapy settings have proven that both exposure and cognitive methods have beneficial effects on PTSD and comorbid mental health symptoms [19-22]. However, little research has investigated the differential effects of cognitive and exposure-based treatments by comparing the 2 treatment techniques with each other directly and with a passive control group in internet-based settings. To the best of our knowledge, no study has addressed this topic in Arabic-speaking populations. Therefore, addressing this issue is of considerable practical relevance, especially in areas with limited access to treatment.

### Study Aims

The aim of this study was to evaluate 2 brief internet-based treatments—1 including CR as the main treatment component and the other including exposure—for Arabic-speaking participants with PTSD. Specifically, we sought to examine the association between the 2 treatment conditions and treatment use investigating the proportion of individuals who started treatment and the proportion who dropped out during treatment as well as treatment duration. We expected that the proportion of treatment starters and dropouts as well as treatment duration would not differ between the 2 treatment conditions. Furthermore, we compared completers’ treatment satisfaction between the 2 treatment conditions and, again, did not expect any differences between conditions. Finally, changes in posttraumatic stress symptom severity, posttraumatic cognitions, anxiety, depressive and somatoform symptom severity, and quality of life during the 2 treatments were examined and compared with those of a waitlist control group. On the basis of previous research, we assumed that both treatments would lead to significant improvements in all treatment outcomes between the baseline assessment and posttreatment time point. We expected that the 2 treatments would lead to similar changes in terms of treatment outcomes and would outperform the waitlist control group.

## Methods

### Trial Information

The study was administered by a psychosocial center for the treatment of war and torture survivors in cooperation with the Freie Universität Berlin and the Medical School Berlin, Germany. The study was preregistered at the German Clinical Trials Register (trial DRKS00010245).

### Participants

This study included Arabic-speaking adults from different countries who were seeking help via the internet for posttraumatic stress and depressive symptoms. As an inclusion criterion, all participants were required to be able to speak, read, and write standard Arabic. Individuals were excluded if they self-reported any of the following in the screening battery: age of <18 years, no private email address or access to a computer and internet, simultaneous psychotherapeutic treatment elsewhere or plans for psychotherapeutic treatment within the next 4 weeks, or severe depressive symptoms (Beck Depression Inventory–II of ≥45). After successfully passing the screening battery, participants underwent a clinical interview in which interviewers checked whether participants met the diagnostic criteria for a depressive disorder or PTSD according to the Diagnostic and Statistical Manual of Mental Disorders, Fifth Edition (DSM-5), assessed using the Structured Clinical Interview for the DSM-5 (SCID-5 Clinical Version) [27] as a requirement for participation in any of the offered treatments. If the diagnostic criteria were not met, the participants were excluded. In the interview, participants were further screened for symptoms of mania or hypomania, psychotic experiences, risk of suicide, drug and alcohol use, and current risk of retraumatization (ie, still living with the perpetrator). We further excluded participants who reported psychotic tendencies, manic or hypomanic episodes, a high risk of suicide (ie, serious suicide attempts within the last 3 y or a current intent), dependency on or abuse of drugs or alcohol with current use, or a current danger of retraumatization. In addition, interviewers checked whether any participants receiving psychopharmacological treatment were on a stable dose and whether participants had completed our treatment program within the previous months. Again, participants who failed to meet these criteria were excluded.

### Procedure

**Figure 1:**
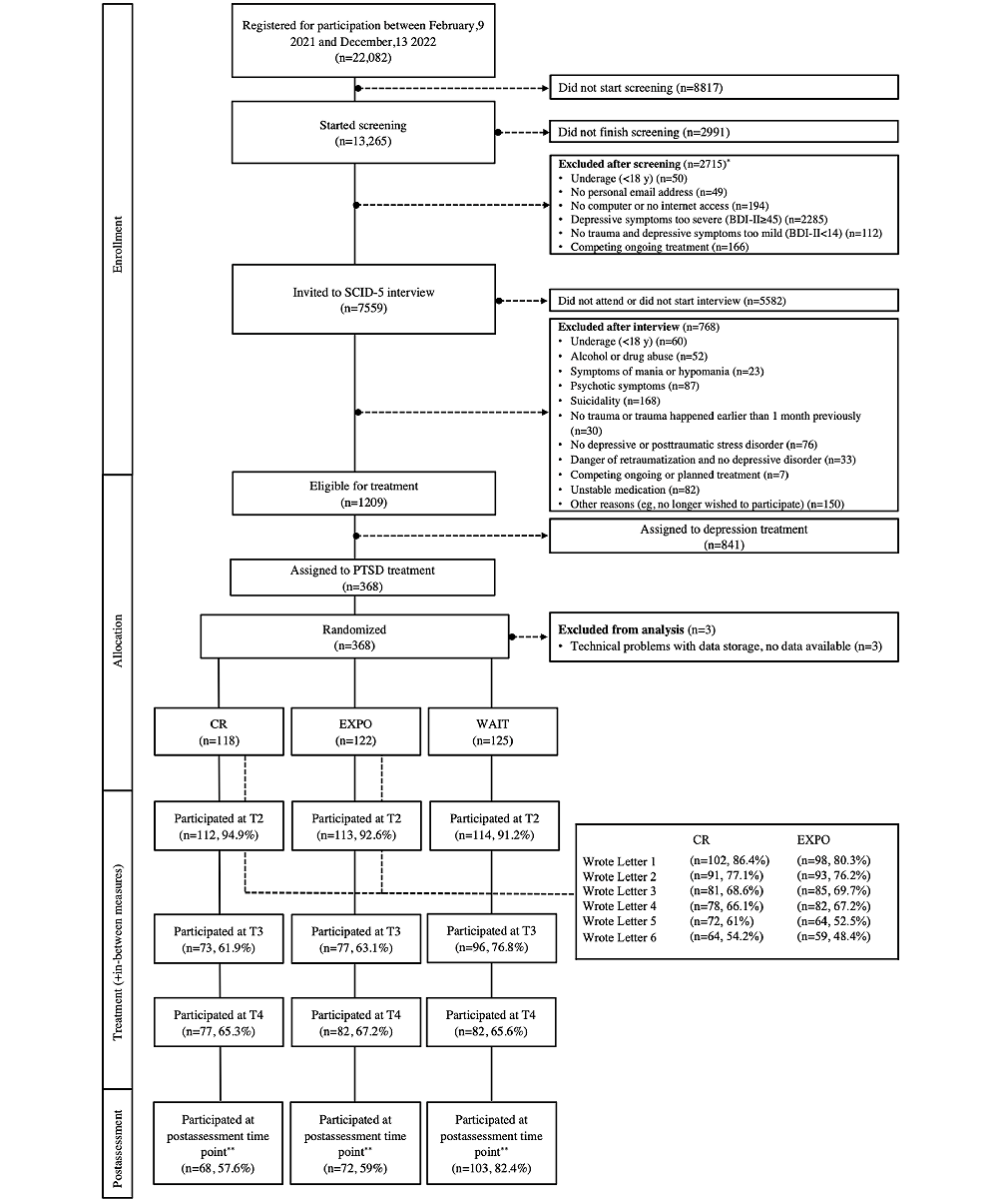
Flowchart. *Some participants fulfill more than one exclusion criterion. **Includes cases that started the assessment without necessarily having completed all questionnaires; BDI-II: Beck Depression Inventory–II; CR: cognitive restructuring treatment; EXPO: exposure treatment; PTSD: posttraumatic stress disorder; SCID-5: Structured Clinical Interview for the Diagnostic and Statistical Manual of Mental Disorders, Fifth Edition; T2: assessment immediately before starting treatment or waiting time; T3: assessment after 2 letters or 1 week of waiting; T4: assessment after 4 letters or 2 weeks of waiting; WAIT: waitlist control condition.

Recruitment took place between February 9, 2021, and December 13, 2022. Participants were recruited through the program’s website (Ilajnafsy [العلاج النفسي], Arabic for *psychotherapy* [28]), word-of-mouth recommendation, and social media (ie, Facebook). Applicants could register on the website for free. The registration page contained information on data security and the terms of participation. Participants were required to provide informed consent (via checkboxes) to receive a confirmation link. After confirming the link, participants could access the password-protected internet portal and begin the web-based screening battery of self-report questionnaires assessing several sociodemographic characteristics, questions on trauma exposure, and clinical characteristics. After successfully completing the screening questionnaires, participants who met the aforementioned inclusion criteria booked an appointment for a clinical interview. Interviews were conducted by trained clinical interviewers by telephone or voice over IP. If further inclusion criteria (outlined previously) assessed in the interview were met, participants were assigned to PTSD or depression treatment based on their primary diagnosis. This study focused only on participants assigned to PTSD treatment. Participants who were deemed eligible for PTSD treatment were randomly allocated to the CR treatment, exposure treatment, or waitlist control group and subsequently assigned to a counselor. Participants allocated to any of the treatments were able to begin treatment after the counselor sent a first letter. At 2 days after the interview, participants who were allocated to the waitlist condition were informed in the password-protected web portal that they would wait for 3 weeks until treatment. During treatment and the waiting time, participants regularly completed a questionnaire on posttraumatic stress symptoms. After completing the treatment or waiting time, participants filled out the set of web-based self-report questionnaires again to examine changes in clinical symptom presentation. Figure 1 shows the flow of participants through the trial.

### Randomization and Blinding

Randomization was performed using block randomization with variable block sizes of 6, 9, and 12. The allocation schedule was created using the R package *Blockrand* (R Foundation for Statistical Computing) [29] and was embedded in the web portal. Allocation to any of the 3 conditions was performed invisibly and automatically on the web portal itself and, thus, was concealed (ie, participants, counselors, and researchers had no previous knowledge of and, therefore, no control over the group to which a participant would be allocated). Owing to the nature of the provided treatments, participants and counselors could not be blinded to the treatment condition received.

### Study Conditions

#### Overview of Treatments

The 2 treatments were based on an internet-based cognitive behavioral treatment approach for PTSD (Interapy) [30]. An overview of the procedure and writing examples of both treatment conditions can be found in Table S1 in Multimedia Appendix 1. The protocols were translated into Modern Standard Arabic. To obtain linguistically and culturally appropriate protocols, we made the following changes: (1) different versions for female and male participants regarding particularities in the Arabic language, (2) strengthening of the advice to not mention real names or places involved in the traumatic event because of basic precautionary measures, (3) use of pictorial metaphors (ie, scar or wound metaphor and linen cupboard metaphor for PTSD) to explain the purpose and process of trauma treatment in a less technical way, and (4) use of an encouraging and motivational but directive writing style. If needed (eg, the participant expressed a high level of faith), counselors could include quotes from the Qur’an. In addition, the layout of the protocols and the technical descriptions within the protocols were adapted to fit the format of the web portal (eg, participants were instructed to use the web-based planner). Both treatments consisted of twice-weekly 45-minute structured writing assignments in the form of letters over a period of approximately 3 weeks (approximately 2 letters/wk). The writing sessions were planned, and participants were instructed to plan the date and hour in which they would write each letter. After receiving each letter from a participant, the counselors provided individual feedback and instructions for the next letter within 2 working days. The feedback and instructions consisted of standard examples that were tailored to the participants’ individual needs and the content of the previous letters. Both treatments began with an introduction by the counselor providing information on writing treatment in general, the procedure of the treatment in detail, and psychoeducational information on PTSD. As the counselors already had knowledge of the traumatic event to be addressed in the treatment through the interview report, they could directly refer to the most distressing traumatic event or associated dysfunctional thoughts and feelings in their first letter. At the beginning of each module, psychoeducational information on the specific treatment phase was provided. In both treatments, a final letter was sent at the end of treatment in which the counselor summarized the participant’s progress during treatment.

#### Exposure Treatment

In addition to the introductory part, the exposure treatment included 2 different phases. In the first phase (self-confrontation), participants were instructed to write 4 letters about the traumatic event and their related thoughts, fears, and physiological reactions. They were asked to describe sensory perceptions in detail and focus on the most distressing situation of the trauma. We also included a section on how traumatic events are processed and why symptoms are maintained, as well as how exposure treatment could help, to make the condition comparable in length with the CR treatment. In the second phase—the social sharing phase—participants were asked to write 2 letters to summarize their memories of the trauma and consider how they were going to deal with the trauma in the future. The social sharing phase focused on a symbolic farewell letter that participants were instructed to address to themselves or to a significant other.

#### CR Treatment

In addition to the introductory part, the CR treatment included 2 different phases. The first phase of CR treatment encompassed 4 letters to reflect on automatic dysfunctional cognitions and adjust unrealistic assumptions (eg, guilt). Participants were instructed to write a letter to a hypothetical friend who had experienced the same traumatic event without necessarily going into the details of the traumatic experience. Compared with the original Interapy protocol, in which the CR phase was implemented after the exposure phase, we had to adapt the CR treatment part to enable participants to begin this phase without the previous knowledge gained through the exposure phase. Therefore, detailed information was provided in advance regarding the impact of traumatic events (ie, how traumatic experiences can influence thoughts and beliefs about oneself, other people, and the world and how these unhelpful thoughts and beliefs lead to emotions such as guilt and shame). In addition, to encourage participants to identify, challenge, and modify unhelpful beliefs, numerous reflective questions (eg, why did the traumatic event occur? What evidence and counterevidence is there that your friend is responsible for what happened?) were included before starting with the writing assignments. The second phase—social sharing—was identical to that described for the exposure treatment.

#### Waitlist Control Group

A comparison with the waitlist control group was conducted to account for the potential influence of elapsed time and quantify the efficacy of the 2 treatments. After a waiting period of 3 weeks, the waitlist participants received an email invitation to start the program. Before starting any of the 2 treatments (to which they had been randomized in advance), they completed all symptom questionnaires.

### Reminder Messages

Participants received automated emails when they were supposed to log in to the portal (eg, when there was a letter or message from the counselor or a writing assignment was due). In addition, they received automated reminders at each step of the procedure if they were inactive. During the registration and screening process, participants received automated reminders after 3 and 7 days of inactivity and were excluded after 14 days of inactivity. During the interview process, they were reminded after 3, 7, and 14 days and excluded after 21 days of inactivity. Participants who had already been included and allocated to one of the conditions received an automated email after 3 and 7 days if they did not respond to the automated invitation (waitlist control group) or if they did not complete the letters on the chosen dates (treatment groups). In addition, if the participants did not respond to the 2 reminder messages, the counselor contacted them by telephone (if possible) to encourage them to continue. If they could not be reached by telephone, a message was sent including a deadline for a response. After 14 days of nonresponse, participants were considered dropouts.

### Counselors

A total of 10 native Arabic-speaking counselors living in Egypt or Germany performed the treatments. All counselors had a diploma in psychology or psychology-related disciplines (eg, social work, counseling, and psychotherapy) or extensive work experience. Counselors received continuous training covering information about and treatment options for PTSD, the fundamentals and technical aspects of internet-based treatments, specific treatment rationales, provision of feedback, and dealing with challenging situations. Furthermore, all counselors attended regular supervision meetings held by experienced psychotherapists. Support for participants via email or telephone was limited to emergency situations (ie, in cases of suicidality or dropout), technical support, or reminders to continue treatment.

### Assessment

#### Structured Clinical Interview

The clinical interview by telephone or voice over IP was conducted in standard Arabic. It consisted of an introductory part (ie, explaining the procedure; informing about data security; and asking about age, current treatment and current medication, and past treatment in Ilajnafsy); a suicide screening measure (suicidal scale of the Mini-International Neuropsychiatric Interview [31]); substance and alcohol screening measures (Alcohol Use Disorders Identification Test [32] and Drug Abuse Screening Test–10 [33]); and relevant parts of the SCID-5 Clinical Version [27], namely, the sections covering PTSD, mood episodes, psychotic and associated symptoms, and a final part. All interviewers completed training on the administration of the interview, attended interviews conducted by an experienced interviewer, and conducted an interview under the supervision of an experienced interviewer with subsequent feedback. In addition, they received weekly supervision.

#### Web-Based Assessment

Primary and secondary outcome measures were self-reported and administered via the internet in a password-protected area. Instruments that were not available in standard Arabic at the time of planning the study were translated using the forward and backward translation method. Initial translation was conducted by a native Arabic-speaking person, and back translation was carried out by a different native speaker who had no knowledge of the original version. Subsequently, the 2 versions were compared, and deviations were discussed by a team of professionals before agreeing on a final version. Moreover, the instruction texts of the original instruments were adapted to fit the web-based format if necessary. Sociodemographic characteristics and exposure to traumatic events were assessed in the screening test battery only. For this purpose, we used items from the Harvard Trauma Questionnaire [34]; the Posttraumatic Diagnostic Scale [35]; and the Life Events Checklist for DSM-5 [36], with a total of 25 items asking about exposure to various potentially traumatic events, as well as the extended version of the Life Events Checklist for DSM-5 asking about further details of the most distressing event. All outcome measures were assessed as part of the screening test battery (baseline assessment; T1) and at the end of treatment or waiting time (postassessment time point; T5). Questionnaires asking about satisfaction with the treatment were administered after participants had completed any of the treatment conditions. In addition, the Posttraumatic Stress Disorder Checklist for the DSM-5 (PCL-5) [37] was administered at 3 measurement time points during treatment (T2: assessment immediately before starting treatment; T3: assessment after 2 letters; T4: assessment after 4 letters). In the waitlist control group, intermediate measures were administered in correspondence with the treatment groups (ie, participants were invited to complete the PCL-5 every week during the waiting period; T2: immediately before starting the waiting time; T3: assessment after 1 wk; T4: assessment after 2 wk).

#### Primary Outcome Measure

Symptoms of posttraumatic stress in the previous month were assessed using the PCL-5 [37]. The PCL-5 is a self-report questionnaire with 20 items that correspond to the DSM-5 PTSD symptoms. Each item is rated on a 5-point scale (from 0 to 4), with higher scores indicating greater symptom severity. A total of 4 subscales that correspond to the 4 different DSM-5 PTSD symptom clusters (re-experiencing, avoidance, negative alterations in cognitions and mood, and hyperarousal) can be differentiated. For each subscale, a sum score was calculated to assess the severity of each DSM-5 symptom cluster. The PCL-5 has proven to be a valid and reliable screening instrument for traumatized Arabic-speaking populations [38]. In this study, the Cronbach α was .87 for the overall scale and ranged from .70 (hyperarousal) to .83 (avoidance) for the subscales.

#### Secondary Outcome Measures

Posttraumatic maladaptive beliefs about the world, others, and the self were assessed using the self-report Posttraumatic Maladaptive Beliefs Scale (PMBS) [39]. The scale encompasses 15 statements that are rated on a 7-point scale (from 1 to 7). For the sake of consistency with the PCL-5, we used a past-month timeline of inquiry for the PMBS. A sum score was calculated to assess overall posttraumatic maladaptive beliefs, with higher scores indicating higher levels of maladaptive beliefs. Sensitivity to changes that can occur during treatment has been demonstrated [39]. The Cronbach α in this study was .75.

Trauma-related guilt cognitions were assessed using the guilt cognitions scale of the self-report Trauma-Related Guilt Inventory (TRGI) [40]. Respondents rate 22 statements on a 5-point scale (from 4 to 0) to indicate the degree to which they believe the statement is true. Mean scores were calculated, with higher scores indicating higher levels of maladaptive guilt cognitions. For the sake of consistency with the PCL-5, a past-month timeline of inquiry was used for the TRGI. The Cronbach α for the guilt cognitions scale in this sample was .90.

Anxiety symptom severity was measured using the self-report Arabic version of the Generalized Anxiety Disorder**–**7 (GAD-7) [41]. An Arabic version of the GAD-7 was used [42]. The questionnaire asks about general anxiety symptoms using 7 items rated on a 4-point scale (from 0 to 3) referring to the previous 2 weeks. The sum score of all items serves as an indicator of generalized anxiety. The GAD-7 has shown poorer psychometric properties in Arabic-speaking populations than in Western populations [42,43]. In this sample, the Cronbach α was .80.

Depressive symptom severity was assessed using the self-report Patient Health Questionnaire–9 (PHQ-9) [44,45]. An Arabic version of the PHQ-9 was used [42]. The PHQ-9 includes 9 items rated on a 4-point scale (from 0 to 3). A sum score was calculated to determine depressive symptoms, with higher scores indicating greater symptom severity. The PHQ-9 has shown good internal consistency in different Arabic-speaking populations [42,46] and has already been used as a treatment outcome measure in patients from an Arab immigrant population receiving internet-based interventions for depression and anxiety [47]. In this sample, the Cronbach α was .79.

Somatoform symptom severity was measured using the self-report Patient Health Questionnaire–15 [48], which assesses somatic symptoms over the previous month. A total of 15 items for women and 14 items for men are rated on a 3-point scale (from 0 to 2). A sum score was calculated, with higher scores indicating greater impairment. The Patient Health Questionnaire–15 is widely used and has shown good psychometric properties in Western samples [48]. It showed good internal consistency in a study with Saudi Arabian university students [46] and was found to be valid in studies conducted with Saudi Arabian primary care patients [43]. In this sample, the Cronbach α was .79.

Quality of life was assessed using the self-report EUROHIS Quality of Life 8-item index, an adapted version of the World Health Organization Quality of Life Questionnaire and its shorter version [49]. The EUROHIS Quality of Life 8-item index assesses markers of quality of life using 8 items rated on a 5-point scale. A general quality of life index was determined by summing all items, with higher scores indicating better quality of life. The Arabic version of the short version of the World Health Organization Quality of Life Questionnaire has demonstrated adequate psychometric properties [50]. In this sample, the Cronbach α was .66.

#### Posttreatment Evaluation Questions

After completing any of the treatment conditions, participants were further asked about their experience of the treatment using the following specific questions: How satisfied were you with the treatment? (5-point scale from *totally satisfied* to *unsatisfied*), Was the treatment helpful? (5-point scale from *very helpful* to *not helpful*), Would you recommend the treatment*?* (5-point scale from *definitely* to *definitely not*), and How do you rate the duration of the treatment? (*too short*, *sufficient*, or *too long*).

### Statistical Analyses

#### Overview of Statistical Analyses

Analyses were conducted using the R statistical software (version 4.2.2) [51] and the Mplus statistical modeling software (version 8, Muthén and Muthén) [52]. All 3 conditions were compared regarding baseline characteristics to see whether randomization worked properly. We examined the association between treatment condition and the proportion of participants who did not start treatment and those who wrote at least one letter (nonstarters vs starters). Nonstarters and starters were further compared in terms of sociodemographic, trauma-related, and clinical characteristics reported at baseline. Similarly, we investigated the association between treatment condition and the proportion of participants who stopped treatment before completing all 6 letters and those who completed all 6 letters (dropouts vs completers). Furthermore, in both treatment conditions, dropouts and completers were compared in terms of sociodemographic, trauma-related, and clinical characteristics reported at baseline. In addition, the duration of both treatment conditions (in days) was compared between both treatment conditions. The results of the posttreatment evaluation questions as markers of treatment satisfaction were compared between the 2 treatment conditions. All the aforementioned group differences were investigated using Welch or chi-square tests. If assumptions for the Welch tests were not met, the Kruskal-Wallis or Mann-Whitney tests were applied. The Fisher exact test was used as an alternative to the chi-square test. Treatment-associated changes in primary and secondary outcome measures across different measurement time points were modeled using multigroup latent change models [53,54]. The models were estimated using the robust maximum likelihood estimator. The rate of change is determined under the assumption that the score at a specific measurement time point after the initial assessment is composed of the initial score and the difference between the initial score and the score obtained at the specific measurement time point after the initial assessment (ie, the postassessment score) [55]. Thus, the rate of change between measurement time points is directly modeled in the form of the change score. The mean of the change scores represents the average change (decrease or increase) between 2 measurements within each condition in units of the questionnaire. Between-group effects are represented by the differences between the group-specific mean change scores. Within-group effect sizes (*d*) were computed by dividing the mean change scores by their SD for each group. Between-group effect sizes (*d*) were computed by dividing the mean difference between the mean change scores of the 2 groups by the pooled SD. All the results of treatment-associated changes were pooled across multiple imputed data sets. Bonferroni correction was applied to maintain the error rate at 0.05 for within-group changes and between-group differences. Therefore, a *P* value of <.005 (adjusted for 11 treatment outcomes) was considered statistically significant for within- and between-group comparisons. To assess reliable changes in individual posttraumatic stress symptom severity between baseline and the postassessment time point in all 3 conditions, we calculated the reliable change index for each participant [56] using the test-retest reliability of *r*=0.82 for the PCL-5 [57] and the SD at baseline of this sample (SD of 13.28 pooled across imputed data sets). According to this calculation, changes in posttraumatic stress symptom severity were considered statistically significant if the difference between baseline and the postassessment time point exceeded 16 points in the PCL-5 (α=.05). The proportions of participants with reliable improvement (16-point decrease minimum) or deterioration (16-point increase minimum) were calculated. Furthermore, we calculated the rates of remitted participants (ie, participants with a baseline PCL-5 value of ≥23 as an indicator of caseness [having PTSD] and a postassessment PCL-5 value of <23). The cutoff value of 23 was chosen based on a study with Arabic-speaking people [38]. The results of the study showed that the PCL-5 achieved the best balance between sensitivity and specificity in the Arabic-speaking sample when this cutoff was used. In addition, the proportions of participants who experienced both reliable and clinically significant improvement (RCSI) were determined. The association between all 3 conditions and the proportion of participants with reliable change, experience of remission, and RCSI was examined using chi-square tests, which were pooled across all imputed data sets [58]. Analyses of treatment-associated changes were conducted on the intention-to-treat (ITT) and completer samples. Completers in both treatment conditions were defined as those participants who completed all 6 letters. In the waitlist control condition, completers were defined as participants who completed all questionnaires of the postassessment time point (T5). The results of the completer analyses can be found in Tables S2-S5 in Multimedia Appendix 1.

#### Missing Data

At baseline, 7 participants (CR treatment: n=3; exposure treatment: n=4) did not provide data for the trauma-related questionnaires (trauma exposure questions, PCL-5, PMBS, and TRGI). Owing to the low rate of missingness, statistical comparisons between specific groups (nonstarters vs starters and dropouts vs completers) that only included baseline scores were conducted using listwise deletion. The rates of missing values at the postassessment time point in the ITT sample with respect to all primary and secondary outcome measures ranged from 42.4% to 48.3% in the CR treatment group, from 41% to 48.4% in the exposure treatment group, and from 17.6% to 19.2% in the waitlist control condition. In the completer sample, rates of missing values at the postassessment time point were lower (CR treatment: 7.8%-12.5%; exposure treatment: 0%-3.4%). To deal with missing data, multiple imputation (100 imputed data sets; 50 iterations) for primary and secondary outcome measures was performed using the R package *MICE* [59]. All outcome measures were used in the imputation model. Predictive mean matching on the level of sum scores was applied for all variables except for the overall sum score of the PCL-5. For the overall sum scores of the PCL-5 at all measurement time points, passive imputation was used to account for the dependency of the overall PCL-5 sum score on the sum scores of the symptom clusters [60]. Multiple imputation was conducted separately for each of the 3 conditions. Following recommended guidelines [61], a sensitivity analysis was conducted to investigate whether deviations from the missing-at-random assumption would affect the conclusions drawn from the results calculated under the assumption that data are missing at random. For the primary outcome measured using the PCL-5, a total of 3 different conditions were modeled for the ITT sample. Individual imputed scores at each measurement time point (after the baseline assessment) increased by 25%, 50%, and 75% for all participants (Tables S6-S8 in Multimedia Appendix 1).

### Ethics Approval

The Ethics Committee of the Freie Universität Berlin approved the study (107/2016).

## Results

### Participants

In total, 365 Arabic-speaking participants (CR treatment: n=118, 32.3%; exposure treatment: n=122, 33.4%; waitlist control: n=125, 34.2%) were included in this study. Participants were mainly female (272/365, 74.5%), single (227/365, 62.2%), living in urban areas (327/365, 89.6%), highly educated (331/365, 90.7%), and young adults (mean age 25.49, SD 6.68; range 18-53 y). The largest shares of participants were from Egypt (96/365, 26.3%), Saudi Arabia (69/365, 18.9%), and Syria (46/365, 12.6%) and were currently residing in Egypt (100/365, 27.4%), Saudi Arabia (60/365, 16.4%), and Jordan (25/365, 6.8%). On average, participants reported 5.19 (SD 3.72) different traumatic events in the trauma exposure questionnaire, with the worst event most frequently involving sexual violence (ie, “sexual assault by family member or acquaintance” [69/358, 19.3%], “sexual contact while under the age of 18 with a person at least 5 years older” [55/358, 15.4%], and “sexual assault by a stranger” [27/358, 7.5%]). PCL-5 scores at baseline ranged between 5 and 77, with a mean of 48.1 (SD 13.31). On average, participants reported an elevated level of depressive (mean 17.55, SD 5.17; range 2-27), anxiety (mean 14.21, SD 4.4; range 2-21), and somatoform (mean 14.5, SD 5.19; range 2-29) symptoms. In addition to having PTSD, most participants (268/365, 73.4%) had a comorbid depressive disorder (current or previous depressive episode, dysthymia, or both [“double depression”]), as assessed using the SCID-5. An overview of the sociodemographic, trauma-related, and clinical characteristics of participants in the total sample and in each condition is displayed in Table 1. No significant differences were found among the conditions regarding sociodemographic or trauma-related characteristics at baseline (Table 1). With regard to outcome measures, differences in anxiety symptom severity were found, with baseline values higher in the waitlist control group than in the 2 treatment conditions (Games-Howell post hoc tests: mean difference of 1.37, 95% CI 0.12-2.61, and *P*=.03 for exposure treatment vs waitlist control; mean difference of 1.41, 95% CI 0.09-2.73, and *P*=.03 for CR treatment vs waitlist control).

**Table 1:** Sociodemographic, trauma-related, and clinical characteristics of the total sample and subsamples in each condition (N=365).

					Total			CR^a^ (n=118)			EXPO^b^ (n=122)			WAIT^c^ (n=125)			*F* test (*df*)^d^				*P* value
**Sociodemographic characteristics**																					
	Age (years), mean (SD)			25.49 (6.68)			25.04 (6.47)			26.39 (7.85)			25.03 (5.51)			0.10 (2)^e^				.61	
	Female sex, n (%)			272 (74.5)			91 (77.1)			91 (74.6)			90 (72)			0.84 (2)^f^				.66	
	**Marital status, n (%)**																	Fisher^g^			.05
		Single	227 (62.2)			78 (66.1)			68 (55.7)			81 (64.8)									
		Married or in a relationship	121 (33.2)			30 (25.4)			51 (41.8)			40 (32)									
		Divorced	15 (4.1)			8 (6.8)			3 (2.5)			4 (3.2)									
		Widowed	2 (0.5)			2 (1.7)			0 (0)			0 (0)									
	**Education, n (%)**																	2.38 (2)^f^			.30
		High education (high school, university, or college diploma)	331 (90.7)			111 (94.1)			109 (89.3)			111 (88.8)									
		Low education (no or intermediate school diploma)	34 (9.3)			7 (5.9)			13 (10.7)			14 (11.2)									
	**Type of residence, n (%)**																	0.13 (2)^f^			.96
		Urban (metropolitan city, small town, or suburb)	327 (89.6)			106 (89.8)			110 (90.2)			111 (88.8)									
		Rural (village or single farmstead)	38 (10.4)			12 (10.2)			12 (9.8)			14 (11.2)									
**Trauma-related characteristics**																					
	Number of different traumatic events (trauma exposure list), mean (SD)			5.19 (3.72)			4.8 (3.39)			5.17 (4.01)			5.59 (3.71)			3.53 (2)^e^				.17	
	Exposure to sexual violence during most distressing trauma (LEC-5^h^), n (%)			113 (31.6)^i^			40 (34.8)^j^			33 (28)^k^			40 (32)			1.27 (2)^f^				.53	
**Clinical characteristics, mean (SD)**																					
	**Posttraumatic stress symptom severity (PCL-5^l^)**																				
		Overall	48.1 (13.31)^i^			46.63 (13.11)^j^			47.77 (13.62)^k^			49.77 (13.12)			1.77 (2, 235.86)				.17		
		Re-experiencing	11.03 (4.68)^i^			10.5 (4.96)^j^			10.85 (4.53)^k^			11.69 (4.51)			3.09 (2)^e^				.21		
		Avoidance	5.12 (2.35)^i^			4.95 (2.35)^j^			5.19 (2.35)^k^			5.22 (2.37)			1.14 (2)^e^				.57		
		Negative alterations in cognitions and mood	18.18 (5.25)^i^			17.89 (5.1)^j^			18.08 (5.47)^k^			18.54 (5.2)			1.68 (2)^e^				.64		
		Hyperarousal	13.77 (4.72)^i^			13.29 (4.83)^j^			13.64 (4.77)^k^			14.32 (4.55)			1.52 (2, 235.21)				.22		
	Posttraumatic maladaptive beliefs (PMBS^m^)		67.08 (13.33)^i^			67.5 (14.77)^j^			66.88 (12.6)^k^			66.86 (12.68)			0.08 (2, 233.72)				.93		
	Trauma-related guilt (TRGI^n^)		1.78 (0.85)^i^			1.81 (0.86)^j^			1.76 (0.85)^k^			1.77 (0.84)			0.28 (2)^e^				.87		
	Anxiety symptom severity (GAD-7^o^)		14.21 (4.4)			13.71 (4.81)			13.75 (4.41)			15.12 (3.84)			7.24 (2)^e^				.03		
	Depressive symptom severity (PHQ-9^p^)		17.55 (5.17)			17.52 (5.11)			17.14 (5.41)			17.98 (5)			1.30 (2)^e^				.52		
	Somatoform symptom severity (PHQ-15^q^)		14.5 (5.19)			14.02 (4.79)			14.38 (5.53)			15.06 (5.19)			1.36 (2, 240.85)				.26		
	Quality of life (EUROHIS-QOL-8^r^)		12.8 (4.43)			12.94 (4.45)			12.91 (4.34)			12.57 (4.51)			0.26 (2, 241.04)				.80		

^a^CR: cognitive restructuring treatment.

^b^EXPO: exposure treatment.

^c^WAIT: waitlist control group.

^d^Group comparisons among all 3 groups are based on the Welch test unless otherwise stated.

^e^Kruskal-Wallis test (test statistic H).

^f^Chi-square test of independence.

^g^Fisher exact test.

^h^LEC-5: Life Events Checklist for the Diagnostic and Statistical Manual of Mental Disorders, Fifth Edition (extended version).

^i^n=358 because of missing data.

^j^n=115 because of missing data.

^k^n=118 because of missing data.

^l^PCL-5: Posttraumatic Stress Disorder Checklist for the Diagnostic and Statistical Manual of Mental Disorders, Fifth Edition.

^m^PMBS: Posttraumatic Maladaptive Beliefs Scale.

^n^TRGI: Trauma-Related Guilt Inventory.

^o^GAD-7: Generalized Anxiety Disorder–7.

^p^PHQ-9: Patient Health Questionnaire–9.

^q^PHQ-15: Patient Health Questionnaire–15.

^r^EUROHIS-QOL-8: EUROHIS Quality of Life 8-item index.

### Use of Treatment

#### Starters Versus Nonstarters

Overall, 16.7% (40/240) of the participants did not start any of the treatments (CR treatment: 16/118, 13.6%; exposure treatment: 24/122, 19.7%) after allocation to treatment. There was no significant association between treatment condition and the proportion of participants who did not start treatment versus those who wrote at least one letter (χ^2^_1_=1.6; *P*=.20). Concerning trauma-related and clinical characteristics, none of the comparisons between nonstarters and starters reached significance (*P*>.05 in all cases). Regarding sociodemographic characteristics, starters included a significantly higher proportion of female participants (158/200, 79%) than nonstarters (24/40, 60%; *P*=.01).

#### Completers Versus Dropouts

Of the 200 participants who began any of the treatments, 123 (61.5%) completed all 6 letters (CR treatment: 64/102, 62.7%; exposure treatment: 59/98, 60%). There was no significant association between treatment condition and the proportion of participants who completed the treatment versus participants who dropped out (*χ*^2^_1_=0.1; *P*=.71). Welch tests revealed significant differences between completers and dropouts in both treatment conditions regarding baseline scores of overall posttraumatic stress symptom severity, negative alterations in cognitions and mood, maladaptive posttraumatic beliefs, depressive symptom severity, and quality of life. Other comparisons were nonsignificant (*P*>.05 in all cases). Games-Howell post hoc tests indicated that, compared with participants who dropped out of the CR treatment, those who completed the CR treatment had lower baseline posttraumatic stress symptom severity (mean difference of 6.93, 95% CI 0.90-12.95; *P*=.02), negative alterations in cognitions and mood (mean difference of 2.69, 95% CI 0.33-5.06; *P*=.02), and maladaptive posttraumatic beliefs (mean difference of 8.39, 95% CI 0.59-16.20; *P*=.03). Furthermore, compared with participants who dropped out of the CR treatment, those who completed either the exposure treatment or the CR treatment had lower depressive symptom severity at baseline, with a mean difference of 2.97 (95% CI 0.47-5.48; *P*=.01) and 2.71 (95% CI 0.20-5.22; *P*=.03), respectively. Quality of life at baseline was significantly higher in participants who completed the CR treatment than in those who dropped out of the CR treatment, with a mean difference of 2.50 (95% CI 0.20-4.80; *P*=.03). Table 2 summarizes the characteristics of participants who dropped out and those who completed any of the treatments, as well as the statistical results.

**Table 2:** Characteristics of and comparison between participants who completed <6 letters (dropouts) and participants who completed all 6 letters (completers).

					EXPO^a^							CR^b^						*F* test (*df*)^c^					*P* value
					Dropouts (n*=*39)			Completers (n*=*59)			Dropouts (n*=*38)			Completers (n*=*64)									
**Sociodemographic characteristics**																							
	Age (years), mean (SD)			24.74 (6.47)			27.81 (8.12)			24.03 (6.11)			24.97 (6.31)			7.08 (3)^d^				.07			
	Female sex, n (%)			28 (72)			47 (80)			30 (79)			53 (83)			1.80 (3)^e^				.62			
	**Marital status, n (%)**																	Fisher^f^				.29	
		Single	22 (56)			34 (58)			24 (63)				44 (69)										
		Married or in a relationship	17 (44)			22 (37)			10 (26)				17 (27)										
		Divorced	0 (0)			3 (5)			3 (8)				3 (5)										
		Widowed	0 (0)			0 (0)			1 (3)				0 (0)										
	**Education, n (%)**																	Fisher^f^				.58	
		High education (high school, university or college diploma)	35 (90)			53 (90)			36 (95)				61 (95)										
		Low education (no or intermediate school diploma)	4 (10)			6 (10)			2 (5)				3 (5)										
	**Type of residence, n (%)**																	Fisher^f^				.07	
		Urban (metropolitan city, small town, or suburb)	36 (92)			54 (92)			30 (79)				61 (95)										
		Rural (village or single farmstead)	3 (8)			5 (8)			8 (21)				3 (5)										
**Trauma-related characteristics**																							
	Number of different traumatic events (trauma exposure list), mean (SD)			5.15 (4.16)			5.58 (3.99)			4.53 (3.03)			4.92 (3.5)			1.82 (3)^d^				.61			
	Exposure to sexual violence during most distressing trauma (LEC-5^g^), n (%)			15 (41)^h^			13 (23)^i^			17 (46)^h^			19 (31)^j^			6.57 (3)^e^				.09			
**Clinical characteristics, mean (SD)**																							
	**Posttraumatic stress symptom severity (PCL-5^k^)**																						
		Overall	47.43 (13.15)^h^			48.25 (12.37)^i^			52.30 (10.85)^h^				45.37 (11.37)^j^		3.02 (3, 94.5)				.03				
		Re-experiencing	10.70 (4.55)^h^			10.82 (4.43)^i^			11.81 (4.64)^h^				10.24 (4.70)^j^		0.87 (3, 94.8)				.46				
		Avoidance	5.22 (2.45)^h^			5.30 (2.20)^i^			5.30 (2.60)^h^				4.97 (2.10)^j^		1.68 (3)^d^				.64				
		Negative alterations in cognitions and mood	17.68 (5.42)^h^			18.37 (5.16)^i^			20.08 (4.13)^h^				17.39 (4.67)^j^		3.19 (3, 95.3)				.03				
		Hyperarousal	13.84 (4.72)^h^			13.75 (4.43)^i^			15.11 (4.35)^h^				12.77 (4.50)^j^		2.15 (3, 94.6)				.10				
	Posttraumatic maladaptive beliefs (PMBS^l^)		68.76 (12.08)^h^			66.65 (13.42)^i^			72.81 (14.09)^h^				64.42 (14.69)^j^		2.83 (3, 96.3)				.04				
	Trauma-related guilt (TRGI^m^)		1.59 (0.75)^h^			1.89 (0.81)^i^			1.92 (0.84)^h^				1.75 (0.89)^j^		1.54 (3, 96.2)				.21				
	Anxiety symptom severity (GAD-7^n^)		14.15 (4.57)			13 (4.46)			14.18 (4.34)				13.34 (4.97)		0.82 (3, 99.8)				.49				
	Depressive symptom severity (PHQ-9^o^)		17.36 (5.9)			16.24 (4.92)			19.21 (4.38)				16.5 (5.15)		3.78 (3, 98.7)				.01				
	Somatoform symptom severity (PHQ-15^p^)		14.95 (4.97)			14.17 (5.97)			15.13 (4.86)				13.67 (4.53)		0.10 (3, 98.4)				.40				
	Quality of life (EUROHIS-QOL-8^q^)		13.28 (4.62)			12.93 (4.38)			11.34 (4.04)				13.84 (4.69)		2.80 (3, 99.7)				.04				

^a^EXPO: exposure treatment.

^b^CR: cognitive restructuring treatment.

^c^Group comparisons based on the Welch test unless otherwise stated.

^d^Kruskal-Wallis test (test statistic H).

^e^Chi-square test of independence.

^f^Fisher exact test.

^g^LEC-5: Life Events Checklist for the Diagnostic and Statistical Manual of Mental Disorders, Fifth Edition (extended version).

^h^n=37 because of missing data.

^i^n=57 because of missing data.

^j^n=62 because of missing data.

^k^PCL-5: Posttraumatic Stress Disorder Checklist for the Diagnostic and Statistical Manual of Mental Disorders, Fifth Edition (extended version).

^l^PMBS: Posttraumatic Maladaptive Beliefs Scale.

^m^TRGI: Trauma-Related Guilt Inventory.

^n^GAD-7: Generalized Anxiety Disorder–7.

^o^PHQ-9: Patient Health Questionnaire–9.

^p^PHQ-15: Patient Health Questionnaire–15.

^q^EUROHIS-QOL-8: EUROHIS Quality of Life 8-item index.

#### Duration of Treatment

Participants who started the CR treatment (102/118, 86.4%) were in treatment for an average of 38.8 (SD 19.7) days. Participants who started the exposure treatment (98/122, 80.3%) were in treatment for an average of 38 (SD 18.4) days. For participants who completed the CR treatment (64/102, 62.7%), the mean treatment duration was 35.2 (SD 15) days. For participants who completed the exposure treatment (59/98, 60%), the mean treatment duration was 32.2 (SD 13.3) days. The duration of treatment did not differ between the 2 treatment conditions either regarding participants who started any of the treatments (*U*=4882; *P*=.78) or regarding participants who completed any of the treatments (*U*=1652; *P*=.23).

### Treatment Satisfaction

Of all completers who answered the evaluation questions, 89% (53/59) in the CR treatment and 86% (51/59) in the exposure treatment were completely satisfied, very satisfied, or satisfied with the treatment (*U*=1867; *P*=.48). Moreover, 93% (55/59) of patients in the CR treatment and 92% (54/59) of patients in the exposure treatment experienced the treatment as very helpful, helpful, or rather helpful (*U*=1920; *P*=.31). In both treatments, 95% (56/59) of the participants would recommend the treatment to someone else (*U*=1787.5; *P*=.78). In terms of treatment duration, 58% (34/59) of the participants in the CR treatment and 64% (38/59) of the participants in the exposure treatment experienced the treatment duration as sufficient (CR treatment: n=0 too long and 25/59, 42% too short; exposure treatment: 1/59, 2% too long and 20/59, 34% too short; *P*=.45).

### Changes in Primary Outcome (ITT Sample)

#### Within-Group Changes

In both treatment conditions, no significant changes in overall posttraumatic stress symptom severity emerged between baseline and T2 (directly before starting treatment) or T3 (after 2 letters). Significant changes in overall posttraumatic stress symptom severity were found between baseline and T4 (after 4 letters; CR treatment: *d=*−0.45; exposure treatment: *d=*−0.54) and between baseline and the postassessment time point (CR treatment: *d=*−1.03 and exposure treatment: *d=*−1.00) in both treatment conditions. With regard to the subscale scores, significant changes between baseline and T3 (after 2 letters) emerged for the “re-experiencing” subscale in the CR treatment (*d=*−0.39) and for the “negative alterations in cognitions and mood” subscale in the exposure treatment (*d=*−0.30). Moreover, changes on the subscales “re-experiencing,” “negative alterations in cognitions and mood,” and “hyperarousal” between baseline and T4 (after 4 letters) were statistically significant in both treatment conditions, ranging from *d=*−0.32 (CR treatment; “hyperarousal” subscale) to *d=*−0.50 (CR treatment; “re-experiencing” subscale). In both treatment conditions, changes in symptom severity from baseline to the postassessment time point were statistically significant for all subscales (effect sizes ranging from *d=*−0.58 [CR treatment; “avoidance” subscale] to *d=*−0.94 [CR treatment; “re-experiencing” subscale]). Participants in the waitlist control condition showed statistically significant changes in overall posttraumatic stress symptom severity between baseline and T3 (after 1 wk of waiting) and T4 (after 2 wk of waiting) and between baseline and the postassessment time point (*d=*−0.29, *d=*−0.34, and *d=*−0.31, respectively). In addition, the waitlist control participants showed significant changes in the “re-experiencing” subscale between baseline and all subsequent measurement time points (effect sizes ranging from *d*=−0.28 to *d*=−0.43). In the “negative alterations in cognitions and mood” subscale, significant changes emerged between baseline and T4 (after 2 wk of waiting; *d=*−0.35). Table 3 provides further information on estimates for within-group changes in posttraumatic stress symptom severity between baseline and each subsequent measurement time point in the ITT sample.

**Table 3:** Estimated within-group changes in posttraumatic stress symptom severity between baseline and subsequent assessments (intention-to-treat sample)^a^.

Outcome (PCL-5^b^) and group			T2^c^–T1^d^							T3^e^–T1							T4^f^–T1						T5^g^–T1				
			M^h^ (SE; 95% CI)		*P* value		*d*		M (SE; 95% CI)			*P* value		*d*		M (SE; 95% CI)			*P* value	*d*		M (SE; 95% CI)			*P* value		*d*
**Overall**																											
	CR^i^	−1.06 (1.00; −3.02 to 0.90)		.29		−0.10		−2.52 (1.16; −4.79 to −0.26)			.03		−0.22		−6.78 (1.54; −9.79 to −3.76)			*<.001* ^j^		−0.45	−19.11 (2.17; −23.36 to −14.86)			*<.001*		−1.03	
	EXPO^k^	−1.54 (1.05; −3.61 to 0.52)		.14		−0.14		−3.81 (1.45; −6.65 to −0.98)			.008		−0.27		−7.76 (1.48; −10.65 to −4.86)			*<.001*		−0.54	−17.00 (1.91; −20.74 to −13.26)			*<.001*		−1.00	
	WAIT^l^	−2.11 (1.05; −4.17 to −0.06)		.04		−0.18		−3.27 (1.08; −5.40 to −1.14)			*.003*		−0.29		−4.28 (1.26; −6.74 to −1.81)			*.001*		−0.34	−3.79 (1.18; −6.09 to −1.48)			*.001*		−0.31	
**Re-experiencing**																											
	CR	−0.88 (0.35; −1.58 to −0.19)		.01		−0.24		−1.52 (0.44; −2.38 to −0.67)			*<.001*		−0.39		−2.23 (0.47; −3.15 to −1.31)			*<.001*		−0.50	−5.36 (0.63; −6.59 to −4.12)			*<.001*		−0.94	
	EXPO	−0.47 (0.33; −1.11 to 0.17)		.15		−0.14		−0.83 (0.45; −1.71 to 0.05)			.06		−0.19		−1.89 (0.45; −2.77 to −1.01)			*<.001*		−0.43	−3.99 (0.61; −5.20 to −2.79)			*<.001*		−0.74	
	WAIT	−1.17 (0.38; −1.92 to −0.42)		*.002*		−0.28		−1.80 (0.41; −2.61 to −0.99)			*<.001*		−0.43		−1.86 (0.45; −2.74 to −0.98)			*<.001*		−0.43	−1.82 (0.43; −2.65 to −0.98)			*<.001*		−0.41	
**Avoidance**																											
	CR	0.06 (0.22; −0.38 to 0.50)		.79		0.03		0.20 (0.26; −0.31 to 0.70)			.44		0.08		0.01 (0.25; −0.48 to 0.50)			.95		0.01	−1.66 (0.33; −2.32 to −1.01)			*<.001*		−0.58	
	EXPO	0.03 (0.20; −0.37 to 0.42)		.89		0.01		−0.12 (0.28; −0.67 to 0.42)			.66		−0.05		−0.49 (0.29; −1.05 to 0.07)			.09		−0.18	−1.84 (0.33; −2.49 to −1.20)			*<.001*		−0.62	
	WAIT	0.33 (0.22; −0.10 to 0.75)		.13		0.14		0.16 (0.22; −0.28 to 0.60)			.47		0.07		0.11 (0.22; −0.32 to 0.54)			.61		0.05	0.25 (0.22; −0.18 to 0.68)			.25		0.11	
**Negative alterations in cognitions and mood**																											
	CR	−0.09 (0.47; −1.00 to 0.83)		.85		−0.02		−0.69 (0.51; −1.69 to 0.31)			.18		−0.14		−2.79 (0.69; −4.15 to −1.44)			*<.001*		−0.42	−7.33 (0.96; −9.22 to −5.44)			*<.001*		−0.91	
	EXPO	−1.01 (0.55; −2.09 to 0.06)		.07		−0.17		−1.90 (0.66; −3.19 to −0.61)			*.004*		−0.30		−3.20 (0.68; −4.53 to −1.87)			*<.001*		−0.47	−6.65 (0.85; −8.32 to −4.98)			*<.001*		−0.92	
	WAIT	−0.83 (0.43; −1.68 to 0.02)		.06		−0.18		−1.08 (0.47; −2.00 to −0.16)			.02		−0.22		−1.75 (0.51; −2.74 to −0.75)			*.001*		−0.35	−1.27 (0.51; −2.26 to −0.28)			.01		−0.24	
**Hyperarousal**																											
	CR	−0.15 (0.41; −0.95 to 0.65)		.71		−0.04		−0.51 (0.46; −1.41 to 0.40)			.27		−0.11		−1.76 (0.57; −2.89 to −0.64)			*.002*		−0.32	−4.76 (0.71; −6.15 to −3.37)			*<.001*		−0.82	
	EXPO	−0.09 (0.36; −0.79 to 0.61)		.80		−0.02		−0.96 (0.47; −1.89 to −0.03)			.04		−0.21		−2.18 (0.51; −3.18 to −1.19)			*<.001*		−0.46	−4.51 (0.62; −5.74 to −3.29)			*<.001*		−0.80	
	WAIT	−0.44 (0.38; −1.19 to 0.31)		.25		−0.10		−0.56 (0.39; −1.33 to 0.21)			.15		−0.14		−0.78 (0.47; −1.70 to 0.15)			.10		−0.17	−0.95 (0.40; −1.74 to −0.16)			.02		−0.23	

^a^All estimates were pooled across 100 imputed data sets.

^b^PCL-5: Posttraumatic Stress Disorder Checklist for the Diagnostic and Statistical Manual of Mental Disorders, Fifth Edition.

^c^T2: assessment immediately before starting treatment or waiting time.

^d^T1: baseline assessment.

^e^T3: assessment after 2 letters or 1 week of waiting.

^f^T4: assessment after 4 letters or 2 weeks of waiting.

^g^T5: postassessment time point.

^h^M: change score (mean change in raw score units of the questionnaire).

^i^CR: cognitive restructuring treatment.

^j^Significant *P* values.

^k^EXPO: exposure treatment.

^l^WAIT: waitlist control group.

#### Between-Group Differences

There were no statistically significant differences between the 2 treatment conditions regarding the magnitude of change in posttraumatic stress symptom severity (overall or subscales) at any measurement time point. Differences between the CR treatment and waitlist control group and between the exposure treatment and waitlist control group regarding the magnitude of change in symptom severity (overall or subscales) only reached significance between baseline and the postassessment time point. The effect sizes for overall posttraumatic stress symptom severity were high between the CR treatment and waitlist control group (*d=*0.98) and between the exposure treatment and waitlist control group (*d=*0.89). The effect sizes for the subscales ranged from *d=*0.44 (exposure treatment vs waitlist control; “re-experiencing” subscale) to *d=*0.90 (CR treatment vs waitlist control; “negative alterations in cognitions and mood” subscale).

Table 4 presents further details on estimated between-group differences in mean change scores and effect sizes with regard to posttraumatic stress symptom severity between baseline and each subsequent measurement time point for the ITT sample.

**Table 4:** Estimated between-group differences in posttraumatic stress symptom severity between baseline and subsequent assessments (intention-to-treat sample)^a^.

Outcome (PCL-5^b^) and group comparison			T2^c^–T1^d^						T3^e^–T1						T4^f^–T1					T5^g^–T1				
			ΔM^h^ (SE; 95% CI)		*P* value		*d*		ΔM (SE; 95% CI)		*P* value		*d*		ΔM (SE; 95% CI)		*P* value	*d*		ΔM (SE; 95% CI)		*P* value		*d*
**Overall**																								
	CR^i^ vs EXPO^j^	−0.48 (1.45; −3.31 to 2.35)		.74		−0.04		−1.29 (1.88; −4.98 to 2.40)		.49		−0.10		−0.98 (2.15; −5.20 to 3.23)		.65		−0.07	2.12 (2.87; −3.51 to 7.74)		.46		0.12	
	EXPO vs WAIT^k^	−0.57 (1.49; −3.49 to 2.35)		.70		−0.05		0.54 (1.80; −2.98 to 4.06)		.76		0.04		3.48 (1.94; −0.32 to 7.29)		.07		0.26	13.21 (2.27; 8.76 to 17.66)		*<.001* ^l^		0.89	
	CR vs WAIT	−1.05 (1.45; −3.90 to 1.80)		.47		−0.10		−0.75 (1.57; −3.83 to 2.34)		.64		−0.07		2.50 (2.00; −1.43 to 6.43)		.21		0.18	15.32 (2.48; 10.47 to 20.18)		*<.001*		0.98	
**Re-experiencing**																								
	CR vs EXPO	0.41 (0.48; −0.52 to 1.35)		.38		0.12		0.69 (0.63; −0.54 to 1.92)		.27		0.17		0.34 (0.66; −0.95 to 1.64)		.60		0.08	1.37 (0.85; −0.31 to 3.04)		.11		0.25	
	EXPO vs. WAIT	−0.70 (0.51; −1.70 to 0.30)		.17		−0.18		−0.97 (0.60; −2.15 to 0.22)		.11		−0.23		0.03 (0.64; −1.23 to 1.28)		.97		0.01	2.18 (0.76; 0.68 to 3.67)		*.004*		0.44	
	CR vs WAIT	−0.29 (0.53; −1.32 to 0.75)		.59		−0.07		−0.27 (0.60; −1.45 to 0.91)		.65		−0.07		0.37 (0.66; −0.92 to 1.66)		.57		0.08	3.54 (0.77; 10.47 to 20.18)		*<.001*		0.70	
**Avoidance**																								
	CR vs EXPO	−0.03 (0.30; −0.62 to 0.56)		.92		−0.01		−0.32 (0.38; −1.07 to 0.43)		.40		−0.12		−0.50 (0.37; −1.24 to 0.23)		.18		−0.20	−0.18 (0.48; −1.13 to 0.77)		.71		−0.06	
	EXPO vs WAIT	0.30 (0.29; −0.28 to 0.88)		.31		0.13		0.28 (0.35; −0.40 to 0.97)		.42		0.11		0.60 (0.36; −0.11 to 1.31)		.10		0.24	2.10 (0.40; 1.31 to 2.89)		*<.001*		0.79	
	CR vs WAIT	0.27 (0.31; −0.34 to 0.87)		.39		0.11		−0.04 (0.34; −0.71 to 0.63)		.91		−0.01		0.10 (0.34; −0.56 to 0.75)		.77		0.04	1.92 (0.40; 1.12 to 2.71)		*<.001*		0.74	
**Negative alterations in cognitions and mood**																								
	CR vs EXPO	−0.93 (0.72; −2.33 to 0.48)		.20		−0.17		−1.21 (0.84; −2.86 to 0.43)		.15		−0.21		−0.41 (0.98; −2.33 to 1.51)		.68		−0.06	0.68 (1.28; −1.82 to 3.19)		.59		0.09	
	EXPO vs WAIT	0.18 (0.70; −1.19 to 1.55)		.80		0.03		0.82 (0.81; −0.76 to 2.41)		.31		0.14		1.45 (0.85; −0.21 to 3.12)		.09		0.24	5.38 (0.99; 3.43 to 7.32)		*<.001*		0.85	
	CR vs WAIT	−0.74 (0.64; −1.99 to 0.50)		.24		−0.15		−0.39 (0.69; −1.74 to 0.97)		.58		−0.08		1.04 (0.86; −0.63 to 2.72)		.22		0.18	6.06 (1.08; 3.95 to 8.17)		*<.001*		0.90	
**Hyperarousal**																								
	CR vs EXPO	0.06 (0.54; −1.00 to 1.12)		.91		0.01		−0.45 (0.68; −1.79 to 0.88)		.51		−0.10		−0.42 (0.77; −1.93 to 1.10)		.59		−0.08	0.25 (0.96; −1.64 to 2.14)		.80		0.04	
	EXPO vs WAIT	−0.35 (0.52; −1.38 to 0.68)		.50		−0.09		0.40 (0.61; −0.80 to 1.60)		.52		0.09		1.40 (0.69; 0.05 to 2.76)		.04		0.30	3.56 (0.75; 2.10 to 5.03)		*<.001*		0.72	
	CR vs WAIT	−0.29 (0.56; −1.39 to 0.81)		.61		−0.07		−0.05 (0.60; −1.24 to 1.13)		.93		−0.01		0.99 (0.75; −0.48 to 2.46)		.19		0.20	3.81 (0.81; 2.22 to 5.39)		*<.001*		0.76	

^a^All estimates were pooled across 100 imputed data sets.

^b^PCL-5: Posttraumatic Stress Disorder Checklist for the Diagnostic and Statistical Manual of Mental Disorders, Fifth Edition.

^c^T2: assessment immediately before starting treatment or waiting time.

^d^T1: baseline assessment.

^e^T3: assessment after 2 letters or 1 week of waiting.

^f^T4: assessment after 4 letters or 2 weeks of waiting.

^g^T5: postassessment time point.

^h^ΔM: difference between group-specific means of change scores.

^i^CR: cognitive restructuring treatment.

^j^EXPO: exposure treatment.

^k^WAIT: waitlist control group.

^l^Significant *P* values.

#### Reliable Change, Remission, and RCSI

Table 5 summarizes the rates of reliable change, remission, and RCSI for all 3 conditions in the ITT sample. The 2 treatment conditions did not significantly differ regarding the rates of participants who experienced reliable change (*P*=.74), remission (*P*=.49), or RCSI (*P*=.52) between baseline and the postassessment time point. The rates of reliable change (*P*<.001), remission (*P*<.001), and RCSI (*P*<.001) differed significantly between the CR treatment and waitlist control group. Similarly, the exposure treatment and waitlist control group differed significantly with regard to rates of reliable change (*P*<.001), remission (*P*<.001), and RCSI (*P*<.001).

**Table 5:** Rates of reliable change, remission, and reliable change and significant improvement (RCSI; intention-to-treat sample)^a^.

			Baseline assessment to postassessment time point (PCL-5^b^), n (%)				
			CR^c^ (n=118)		EXPO^d^ (n=122)		WAIT^e^ (n=125)
**Reliable change**							
	Improvement	65.56 (55.6)		68.4 (56.1)		22.68 (18.1)	
	No change	48.96 (41.5)		48.75 (40)		93.27 (74.6)	
	Deterioration	3.48 (2.9)		4.85 (4)		9.05 (7.2)	
Remission			48.44 (41.1)		45.63 (37.4)		4.65 (3.7)
RCSI			43.38 (36.8)		42.04 (34.5)		3.35 (2.7)

^a^All results were averaged across imputed data sets; therefore, the counts contain decimals.

^b^PCL-5: Posttraumatic Stress Disorder Checklist for the Diagnostic and Statistical Manual of Mental Disorders, Fifth Edition. An increase or decrease of 16 PCL-5 points between the baseline and postassessment time points was defined as reliable deterioration or improvement, respectively. Remission was defined as a score of ≥23 at baseline and a score of <23 at the postassessment time point. RCSI was defined as experiencing both remission and reliable improvement from baseline to the postassessment time point.

^c^CR: cognitive restructuring treatment.

^d^EXPO: exposure treatment.

^e^WAIT: waitlist control group.

### Changes in Secondary Outcomes (ITT Sample)

#### Within-Group Changes

In both the CR and exposure treatments, all changes in secondary outcome measures between the baseline and postassessment time points were significant. In the ITT sample, the effect size estimates ranged from *d=*−0.41 (exposure treatment; trauma-related guilt) to *d=*−1.10 (CR treatment; depressive symptom severity). In the waitlist control condition, the levels of depressive and anxiety symptoms as well as quality of life also changed significantly (*d=*−0.50, *d=*−0.44, and *d=*0.33, respectively). Other changes were nonsignificant. The estimated within-group changes for all secondary outcomes between baseline and the postassessment time point in the ITT sample are shown in Table 6.

**Table 6:** Estimated within-group changes and between-group differences for secondary outcomes between baseline and the postassessment time point (intention-to-treat sample)^a^.

Outcome and group		Within-group changes				Group comparison	Between-group differences		
		M^b^ (SE; 95% CI)	*P* value	*d*			ΔM^c^ (SE; 95% CI)	*P* value	*d*
**Posttraumatic maladaptive beliefs (PMBS^d^)**									
	CR^e^	−15.07 (2.26; −19.50 to −10.64)	*<.001* ^f^	−0.73	CR vs EXPO^g^		3.66 (3.05; −2.31 to 9.63)	.23	0.20
	EXPO	−11.41 (1.93; −15.19 to −7.64)	*<.001*	−0.74	EXPO vs WAIT^h^		10.57 (2.23; 6.19 to 14.95)	*<.001*	0.77
	WAIT	−0.84 (1.14; −3.08 to 1.39)	.46	−0.07	CR vs WAIT		14.22 (2.51; 9.30 to 19.15)	*<.001*	0.86
**Trauma-related guilt (TRGI^i^)**									
	CR	−0.44 (0.09; −0.62 to −0.26)	*<.001*	−0.60	CR vs EXPO		0.07 (0.14; −0.21 to 0.34)	.64	0.08
	EXPO	−0.37 (0.11; −0.58 to −0.16)	*.001*	−0.41	EXPO vs WAIT		0.26 (0.12; 0.02 to 0.51)	.03	0.34
	WAIT	−0.11 (0.06; −0.23 to 0.01)	.09	−0.18	CR vs WAIT		0.33 (0.11; 0.12 to 0.54)	*.002*	0.49
**Anxiety symptom severity (GAD-7^j^)**									
	CR	−5.34 (0.69; −6.70 to −3.98)	*<.001*	−0.89	CR vs EXPO		0.30 (1.01; −1.68 to 2.28)	.77	0.05
	EXPO	−5.04 (0.69; −6.38 to −3.69)	*<.001*	−0.87	EXPO vs WAIT		2.95 (0.82; 1.33 to 4.56)	*<.001*	0.56
	WAIT	−2.09 (0.46; −2.98 to −1.19)	*<.001*	−0.44	CR vs WAIT		3.25 (0.83; 1.63 to 4.87)	*<.001*	0.60
**Depressive symptom severity (PHQ-9^k^)**									
	CR	−7.75 (0.86; −9.43 to −6.07)	*<.001*	−1.10	CR vs EXPO		1.14 (1.16; −1.13 to 3.41)	.33	0.16
	EXPO	−6.61 (0.80; −8.17 to −5.04)	*<.001*	−0.95	EXPO vs WAIT		4.06 (0.94; 2.23 to 5.90)	*<.001*	0.67
	WAIT	−2.55 (0.49; −3.50 to −1.59)	*<.001*	−0.50	CR vs WAIT		5.20 (0.99; 3.25 to 7.15)	*<.001*	0.85
**Somatoform symptom severity (PHQ-15^l^)**									
	CR	−2.80 (0.64; −4.04 to −1.55)	*<.001*	−0.50	CR vs EXPO		0.44 (0.90; −1.33 to 2.21)	.62	0.08
	EXPO	−2.36 (0.62; −3.58 to −1.13)	*<.001*	−0.43	EXPO vs WAIT		2.12 (0.75; 0.65 to 3.58)	.005	0.44
	WAIT	−0.24 (0.40; −1.02 to 0.55)	.55	−0.06	CR vs WAIT		2.56 (0.77; 1.06 to 4.06)	*.001*	0.52
**Quality of life (EUROHIS-QOL-8^m^)**									
	CR	5.18 (0.74; 3.72 to 6.64)	*<.001*	0.83	CR vs EXPO		−1.19 (1.10; −3.35 to 0.98)	.28	−0.19
	EXPO	3.99 (0.74; 2.53 to 5.45)	*<.001*	0.66	EXPO vs WAIT		−2.63 (0.85; −4.29 to −0.96)	*.002*	−0.51
	WAIT	1.36 (0.41; 0.56 to 2.17)	*.001*	0.33	CR vs WAIT		−3.81 (0.85; −5.48 to −2.15)	*<.001*	−0.72

^a^All estimates were pooled across 100 imputed data sets.

^b^M: change score (mean change in raw score units of the questionnaire).

^c^ΔM: difference between group-specific means of change scores.

^d^PMBS: Posttraumatic Maladaptive Beliefs Scale.

^e^CR: cognitive restructuring treatment.

^f^Significant *P* values.

^g^EXPO: exposure treatment.

^h^WAIT: waitlist control group.

^i^TRGI: Trauma-Related Guilt Inventory.

^j^GAD-7: Generalized Anxiety Disorder–7.

^k^PHQ-9: Patient Health Questionnaire–9.

^l^PHQ-15: Patient Health Questionnaire–15.

^m^EUROHIS-QOL-8: EUROHIS Quality of Life 8-item index.

#### Between-Group Differences

The results revealed no evidence of differences in the magnitude of change for any secondary outcome measure between the 2 treatment conditions. Significant differences in the magnitude of change between the exposure treatment and waitlist control group and between the CR treatment and waitlist control group were found with regard to posttraumatic maladaptive beliefs (*d=*0.77 and *d=*0.86, respectively), anxiety symptom severity (*d=*0.56 and *d=*0.60, respectively), depressive symptom severity (*d=*0.67 and *d=*0.85, respectively), and quality of life (*d=*−0.51 and *d=*−0.72, respectively). The differences in the magnitude of change regarding somatoform symptom severity (*d=*0.52) and trauma-related guilt cognitions (*d=*0.49) were significant only between the CR treatment and waitlist control group. The estimated between-group differences between baseline and the postassessment time point for all secondary outcomes in the ITT sample are shown in Table 6.

### Sensitivity Analysis (ITT Sample)

The results of the sensitivity analysis for the ITT sample are reported in Tables S6-S8 in Multimedia Appendix 1. For the CR treatment and exposure treatment, changes in overall posttraumatic stress symptom severity between baseline and the postassessment time point were statistically significant when individual values increased by 25% or 50%. Effect sizes decreased to *d=*−0.79 and *d=*−0.57 in the CR treatment and to *d=*−0.75 and *d=*−0.51 in the exposure treatment. Under the most conservative *missing not at random* (MNAR) assumption, with a 75% increase in individual imputed values, only changes within the CR treatment remained statistically significant (*d=*−0.38). For the waitlist control condition, comparisons were either nonsignificant or marked by a significant increase in posttraumatic stress symptom severity.

The results of the between-group comparisons showed no significant differences between the 2 treatment conditions and the waitlist control condition regarding the magnitude of change in posttraumatic stress symptom severity between baseline and the second, third, and fourth measurement time points. In addition, no significant differences were found between the 2 treatment conditions at any measurement time point.

Differences between the exposure treatment and waitlist control group in the magnitude of change regarding overall posttraumatic stress symptom severity between baseline and the postassessment time point were significant under the 25% and 50% MNAR assumptions. Differences between the CR treatment and waitlist control group regarding the magnitude of change in overall posttraumatic stress symptom severity between baseline and the postassessment time points were significant under all 3 MNAR assumptions.

## Discussion

### Principal Findings

This study aimed to evaluate an internet-based CR treatment and an exposure treatment for Arabic-speaking participants with PTSD in terms of efficacy and course of PTSD symptom severity during treatment. First, we sought to examine the association between the 2 treatment conditions and treatment use. For this purpose, we investigated the proportion of participants who started treatment and the proportion of those who dropped out during treatment in both treatment conditions. Overall, 16.7% (40/240) of participants did not start treatment, which is similar to previous studies conducted in internet-based and face-to-face settings in different populations [11,62,63]. This finding underlines that, regardless of the treatment format and the characteristics of the specific sample, a substantial number of people drop out before attending the first session. In this sample, female participants seemed to be more likely to start treatment. The proportion of participants who dropped out after starting treatment was 38.5% (77/200), which is similar to the dropout rates reported in a study investigating different trauma-focused cognitive behavioral interventions for PTSD in a face-to-face setting [63]. The dropout rate in our study was indeed higher than the mean dropout rate of 20.9% reported in a recent meta-analysis of guideline-recommended PTSD treatments [64]. The highest mean dropout rates in the meta-analysis were reported for cognitive processing therapy (34%) and prolonged exposure (28.7%). However, heterogeneity was high across the studies in this meta-analysis. The dropout rate in our study was also higher than that reported in a previous study using the same short internet-based exposure treatment protocol in an Arabic-speaking population with PTSD as in this study [11]. A possible explanation for this discrepancy might lie in the changing circumstances of living conditions during the COVID-19 pandemic. This study was conducted during the pandemic, which was marked by multiple lockdowns. This might have led to a lower degree of privacy or even endangerment at home (ie, an increase in domestic violence [65]), making it difficult to continue with the treatments. Although internet-based interventions are especially important when physical contact is limited, a minimum of privacy is required to engage in treatment. During the pandemic, it may have been especially difficult for the participants to maintain their privacy as many young unmarried adults in Arabic-speaking countries tend to co-reside with other family members (eg, parents) because of the high value placed on family ties [66]. Furthermore, it is possible that participants could not continue treatment because of a SARS-CoV-2 infection.

There was no significant association between treatment condition and the proportion of participants who did not start treatment versus those who wrote at least one letter. Further, no significant association between treatment condition and the proportion of dropouts and completers was found. The duration of treatment was also similar in both treatment conditions. These findings demonstrate that the 2 treatment conditions were comparable with regard to the use of the treatments. Thus, exposure treatment does not generally seem to be more “aversive” than CR treatment (without detailed confrontation of the trauma) in Arabic-speaking participants even though many participants in this sample had experienced sexual violence, which might be associated with a strong fear of disclosing details of traumatic events [67]. In line with this lack of difference between the 2 treatment conditions regarding treatment use, Gutner et al [63] reported similar proportions of nonstarters versus starters and dropouts versus completers across multiple face-to-face treatment conditions with female participants with PTSD after experiences of interpersonal violence. Thus, the use of different treatment methods seems to be independent of the specifics of different trauma samples. Notably, some differences between completers and dropouts emerged in our study, mainly in the CR treatment. Participants who completed CR treatment were less impaired by general posttraumatic stress and depressive symptoms and reported lower levels of negative alterations in cognitions and mood and posttraumatic maladaptive beliefs. In a study investigating female veterans with military sexual trauma who received cognitive processing therapy, lower trauma-related negative cognitions about the self emerged as a protective factor against attrition, and lower negative cognitions about others or the world were associated with a higher number of sessions (albeit not statistically significantly) [68]. However, research findings on predictors of dropout are inconsistent, with a previous meta-analysis finding no systematic differences across different interventions [69]. Furthermore, the treatment satisfaction of completers was examined and compared between the 2 treatments. In this study, the participants who completed any of the treatments seemed to be satisfied with the treatment and experienced it as helpful, highlighting that a good working alliance, which forms the basis for therapeutic processes, can be established in highly standardized internet-based treatments in Arabic-speaking populations [70]. However, in both conditions, nearly half of completers who answered the evaluation questions (45/118, 38.1%) reported that they experienced the treatment as too short. Thus, greater flexibility regarding treatment duration may be required for our sample of Arabic-speaking people with PTSD, who in addition may be struggling with the instability of living conditions in many countries in the region. For instance, it may be necessary to provide a variable treatment duration, with more sessions offered to those who still feel in need of treatment or may not have benefited sufficiently at the symptom level. In summary, a more personalized and tailored treatment approach (ie, concerning the number of sessions) to match the needs and characteristics of the participants might be necessary for internet-based settings as well.

Finally, we examined changes in posttraumatic stress symptom severity, posttraumatic cognitions, anxiety, depressive and somatoform symptom severity, and quality of life between baseline and the postassessment time point and compared them among all 3 conditions. Consistent with previous research on internet-based trauma-focused interventions in Arabic-speaking people [14,15], both treatments resulted in significant changes in PTSD symptom severity between baseline and the postassessment time point and performed better than the waitlist control group. Between baseline and the postassessment time point, we found large effects for overall PTSD symptom severity and for most symptom clusters within both treatment conditions. Most participants reliably improved during any of the treatment conditions (65.6/118, 55.6% in CR treatment and 68.4/122, 56.1% in exposure treatment). This suggests that internet-based interventions with a focus on processing the traumatic experience are applicable in Arabic-speaking populations independent of the specific treatment method being applied. Although the mean duration of both treatment conditions was slightly longer than intended (mean duration of approximately 35, SD 15 d for CR treatment and 32, SD 13 d for exposure treatment for completers), the treatments were still much shorter than most cognitive behavioral treatments conducted in a face-to-face setting. These findings highlight the potential of brief trauma-focused interventions delivered via the internet to close the treatment gap in the region. Interestingly, significant changes in PTSD symptom severity within both conditions mainly emerged after 4 letters, with no significant differences between the 2 conditions. This corresponds to the findings of a previous study in veterans with PTSD, which reported that a median of 4 sessions of prolonged exposure treatment was necessary to achieve symptomatic changes [71]. Differences in the magnitude of change between the exposure treatment and waitlist control groups, as well as between the CR treatment and waitlist control groups, were significant for the comparison between baseline and the postassessment time point. Therefore, despite their differing theoretical foundations, exposure and cognitive treatment techniques did not appear to target different PTSD symptom clusters at different time points as the 2 treatment conditions were comparable regarding changes in the 4 PTSD symptom clusters. These findings are in line with those of studies comparing exposure and cognitive techniques in face-to-face settings [18-20], which similarly revealed no differences in efficacy between the 2 techniques. Notably, some significant symptom changes between baseline and the postassessment time point were also observed in the waitlist control group, with re-experiencing symptoms declining significantly leading to a significant decline in overall PTSD symptom severity, although the effect sizes were small. A meta-analysis by Bradley et al [21] encompassing 15 randomized controlled trials also found a small “no-treatment” effect on PTSD symptoms. Nevertheless, a significant change in overall posttraumatic stress symptom severity between baseline and the postassessment time point could not be detected in the waitlist control group under MNAR conditions.

In line with previous research with Arabic-speaking people [11], changes in secondary outcomes between baseline and the postassessment time point were significant in both treatment conditions. In our sample, the largest effects for within-group changes were found for depressive and anxiety symptom severity, and the lowest effects were found for somatoform symptom severity and trauma-related guilt cognitions. As the 2 treatment conditions did not differ significantly in any of the secondary outcomes, they appear to be equally effective for the treatment of other comorbid mental health symptoms, posttraumatic cognitions, and quality of life aspects in Arabic-speaking populations. The treatment conditions outperformed the waitlist control group in terms of most secondary outcomes, with mostly medium-sized effects.

The sensitivity analysis did not alter the conclusions regarding changes within both treatment conditions as well as differences among all conditions under the 25% and 50% MNAR assumptions for overall posttraumatic stress symptom severity between baseline and the postassessment time point. Under the most conservative 75% MNAR assumption, only changes in overall posttraumatic symptom severity between baseline and the postassessment time point in the CR treatment remained significant. With regard to within-group changes during treatment, the results of the sensitivity analysis provided some indication that, during the first sessions, a positive change seems unlikely.

### Limitations

Some limitations need to be considered when interpreting our findings. The sample represented a rather specific group of the Arabic-speaking population as it mainly comprised female, young, and well-educated participants living in metropolitan areas. It seems that young and well-educated people with a stable internet connection are more familiar with new media and are more comfortable using the internet as a medium to receive treatment. In line with this, research has shown that women are more likely to use the internet for health purposes and that youth and higher education seem to positively affect health-related internet use [72]. Moreover, because of limited personnel resources, we were unable to assess the participants’ clinical status at the end of treatment by means of a clinical interview. Thus, the results on the efficacy of the intervention are based on self-report measures. Finally, because of the limited follow-up data, we did not study the long-term effects of the interventions.

### Conclusions and Outlook

In summary, the results of this study suggest that both exposure and CR treatment delivered via the internet are effective for Arabic-speaking people with PTSD. Even though both treatments were very brief, they performed significantly better than a waitlist control group, showing the high potential of brief trauma-focused cognitive behavioral treatment delivered via the internet to serve more people in need of treatment for symptoms of PTSD. Both treatments seem to lead to similar changes in PTSD and other comorbid mental health symptoms. It is worth mentioning that we observed higher attrition rates than in previous studies, which might be due to the COVID-19 pandemic and the associated potential lack of privacy at home. In view of the numerous challenges faced by Arabic-speaking people and the limited resources for mental health problems, internet-based interventions are a suitable option as they can be delivered independently of location, can be received at home, and have a short duration. In areas where more psychological help for mental health problems is needed, the interventions can be scaled up accordingly. Nevertheless, it is essential to gain more knowledge about those individual participants who did not benefit from any of the treatments (ie, reasons for dropout or nonimprovement). Therefore, future research needs to differentially examine predictors of treatment outcomes for both treatment types to obtain greater insights into whether specific groups of Arabic-speaking participants (ie, those with particular symptom profiles or higher posttraumatic emotions, cognitions, and behavior at baseline) benefit more from one treatment or the other and whether further adaptations are needed. In addition, it would be fruitful to examine participants’ individual trajectories of change to identify specific groups of individuals and examine the characteristics of those who dropped out of any of the treatments (ie, through qualitative interviews). Future research should also examine the long-term effects of both treatment types.

## Appendix

Multimedia Appendix 1 Overview of both treatment conditions with writing examples, results of completer analyses, and results of sensitivity analysis.

Multimedia Appendix 2 CONSORT-eHEALTH checklist (V 1.6.1).

## Abbreviations

CR: cognitive restructuring
DSM-5: Diagnostic and Statistical Manual of Mental Disorders, Fifth Edition
GAD-7: Generalized Anxiety Disorder–7
ITT: intention-to-treat
MNAR: missing not at random
PCL-5: Posttraumatic Stress Disorder Checklist for the Diagnostic and Statistical Manual of Mental Disorders, Fifth Edition
PHQ-9: Patient Health Questionnaire–9
PMBS: Posttraumatic Maladaptive Beliefs Scale
PTSD: posttraumatic stress disorder
RCSI: reliable and clinically significant improvement
SCID-5: Structured Clinical Interview for the Diagnostic and Statistical Manual of Mental Disorders, Fifth Edition
TRGI: Trauma-Related Guilt Inventory
