# Supplementary material for: Exposure Versus Cognitive Restructuring Techniques in Brief Internet-Based Cognitive Behavioral Treatment for Arabic-Speaking People With Posttraumatic Stress Disorder: Randomized Clinical Trial
Source: JMIR Ment Health. 2023 Dec 13;10:e48689. doi: 10.2196/48689 (PMC10753431; doi:10.2196/48689)
Supplement: Multimedia Appendix 1 [file mental_v10i1e48689_app1.docx]

**Supplementary material**

**Table S1.** Overview of both treatment conditions (EXPO^a^ and CR^b^) with writing examples of counselors^c^.

| **Week 1 (Letters 1 and 2; introduction + trauma exposure or cognitive restructuring)** |
| --- |
| ***Counselor task:*** write letter C1^d^ (welcome patient, introduction of counselor, introduction to treatment, psychoeducation PTSD). C1 is identical for both treatment conditions. |
| *Example excerpt from C1:*  Welcome to our online program Ilajnafsy. Thank you very much for your patience and for answering some questions about your complaints.  (…)  First of all, I would like to introduce myself to you: my name is <…>, I am a counselor at Ilajnafsy, an internet-based program for Post-Traumatic Stress Disorder for Arabic-speaking people.  (…)  As you might already know, the program will last approximately at least three weeks. During this period, I will ask you to write six texts about what you have experienced. You will receive specific instructions from me to start processing the trauma. You will write approximately twice a week for 45 minutes each time. I will give you feedback on your texts and suggestions for the following texts.  (…)  Now let’s start with the content of the program. You described that <use the trauma description from the interview report>. I can imagine that this experience and the painful memories and feelings associated with it are still bothering you.  (…)  It may be that you feel worse at the beginning because you are more intensively involved with the traumatic experience. Please don't let this discourage you.  (…) |
| ***Patient task:*** assessment T2^e^, read letter C1, plan writing letters P1^f^ and P2^g^ |
| ***Counselor task:*** write letter C2^h^ (psychoeducation PTSD, rational for treatment, instruction for letters P1 and P2). |
| *Example excerpt from C2 (EXPO):*  (…)  First of all, it is completely normal that you are burdened after a traumatic experience and suffer from symptoms such as nightmares, frequent waking up, physical tension, feelings such as anger or annoyance. In some cases, these symptoms lessen over time. However, some people develop a long-lasting stress reaction called Posttraumatic Stress Disorder.  (…)  You can think of the different parts of the memory like pieces of clothes in a closet. As long as the clothes are unsorted in the closet, they press against the closet door. If you don't pay attention or open the door to take out a sweater, for example, everything comes out. Only when you pick up all the clothes one by one, look at them, fold them neatly and put them in the right place in the closet, will you eventually be able to close the closet door without the closet constantly threatening to burst open again. We will do the same with your memories. Processing the trauma is done by writing down the feelings, thoughts, and situations that you normally try to avoid. At first, this sounds scary. However, by confronting yourself, you might make the experience that you don't have to be afraid of your own memories.  (…)  Once you have decided which event and moment of the event you will write about, focus on that specific moment for a few minutes first. Begin writing when you can recall the situation. Choose one moment from your traumatic event. One moment that you can hardly bear to think about, but that keeps intruding on your thoughts. Write down the most painful memories and emotions you have and describe everything that you experienced, every feeling, thought and physical reaction in as much detail as possible.  (…)  Write in the first person and in the present tense, as if you were experiencing the event at that moment. To assist you in this, the rest of the instructions are written in the present tense.  (…)  *Example excerpt from C2 (CR):*  (…)  First of all, it is completely normal that you are burdened after a traumatic experience and suffer from symptoms such as nightmares, frequent waking up, physical tension, feelings such as anger or annoyance. In some cases, these symptoms lessen over time. However, some people develop a long-lasting stress reaction called Posttraumatic Stress Disorder.  (…)  Studies have shown that a traumatic experience can affect how someone views life, e.g. the way you think about the world, others and yourself. Negative and radical thoughts may form or get worse and can hinder you from moving on.  (…)  These negative changes can have long-term consequences that are often not associated with the upsetting experience. Possible reaction are:  - Feelings of guilt  - Feelings of insecurity in certain situations  - Feeling less self-confidence  - Feeling powerlessness  (…)  The following texts should help you to look at your traumatic experience from a different perspective. It may be that you have experienced several serious events or that different events have arisen from the same stressful situation. In such a case, it would be important to choose one clear event or experience to focus on. You do not need to write down details about the traumatic event itself.  (…)  Imagine you are writing a supportive letter to a friend who experienced the same situation as you.  (…)  Assume that your friend is struggling with the same thoughts and feelings you are having, such as <insert information from interview report>.  (…)  What other ideas can you give him or her regarding the situation, causing <( e.g., guilt, use the information from the interview report>?  (…)  The following questions are designed to help you formulate your advice. You do not need to address them point by point in your letter, but they serve to give you ideas.  (…)  Could your friend has foreseen what happened?  Has something similar happened before, which would have allowed your friend to know how he or she should have reacted in this situation?  (…)  Do you resent him or her for acting the way he or she did?  Who was responsible for the event? Was your friend responsible? Is there evidence that your friend was responsible for what happened?  (…)  Do you think it is reasonable for your friend to continue thinking about it in this way? Or are there other aspects to describe your own experience?  What are your own attitudes, your view of the world? If your friend had taken the same attitude, what would you advise him/her to do?  (…) |
| ***Patient task:*** read letter C2, writing letters P1 and P2, plan writing letters P3^i^ and P4^j^ |
| **Week 2 (Letters 3 and 4; trauma exposure or cognitive restructuring)** |
| ***Counselor task:*** write letter C3^k^ (feedback to letters P1 and P2, instruction for letters P3 and P4). |
| *Example excerpt from C3 (EXPO):*  (…)  You have written about <…>. How painful the moment was and how you felt during the event. I'm sure it wasn't easy.  I would like to ask you, in the next texts, to choose a specific moment from <...>. A moment that you hardly think about, that still bothers you a lot. Most often, this is a moment that keeps coming back to you strongly, triggering feelings and causing physical reactions such as sweating, cold hands, or trepidation.  Again, write in the first person and in the present tense. Describe the moment you have chosen as accurately as possible. Every detail is important: what you see, what you smell, what you hear. Also describe everything that is going on inside you, every feeling, every thought, and how your body reacts (e.g., sweaty palms or a dry mouth).  (…)  Again, before writing, think about what you will do afterwards to relax and recover from reliving the experience.  Write as spontaneously as possible, do not censor yourself. You do not need to look for "nice" phrases. Bad words or repetition are allowed. Grammar and spelling are not important.  Try to think back to the event for 2 minutes before writing. Imagine an object related to the traumatic event or place that object (e.g., photo, clothes) on your desk. Maybe put on some music that reminds you of what happened. Then read your first two texts again and then start writing.  (…)  *Example excerpt from C3 (CR):*  (…)  You described very clearly in both texts that <....>. I can well understand that this experience and the painful memories associated with it are still bothering you. You are immensely brave that you managed to write a letter despite the pain that writing might causes in you.  The negative thoughts and beliefs you describe often occur as a result of trauma and affect how we think and act. I can very well imagine that writing the letters was exhausting and took a lot of strength. In the following, I would like you to continue the letter in the next two texts. In particular, address the following:  < insert negative thoughts; pay attention to expressions of  - guilt  - shame inducing thoughts  - decreased self-confidence  - Lack of trust in others  - powerlessness  - diminished self-esteem  - no respect for others  - difficulty with intimacy  - insecurity with others  - insecurity in certain situations  (…) |
| ***Patient task:*** assessment T3^l^, read letter C3, writing letters P3 and P4 |
| **Week 3 (Letters 5 and 6; social sharing)** |
| ***Counselor task:*** write letter C4^m^ (feedback to letters P3 and P4, instructions for next letters). Parts of C4 are identical for both treatment conditions. |
| *Example excerpt from C4:*  (…)  You have made a great effort and done a good job. It was certainly not easy for you. <Summarize main points of the last four letters, e.g. you have described well that…; Point out what might need to be addressed in the future>. In the previous weeks, you wrote about your trauma. In your writing, you have expressed your feelings and thoughts regarding this event.  (…)  In the next texts, you will write a farewell letter. The fifth text will be the "rough draft" of the farewell letter, and the sixth will be the final version. The purpose of this letter is to provide a symbolic closure to the past. Your final letter should be a document you can be proud of. You can keep it and read it again or (later) give it to other people to read, trusted people you want to explain what happened to you. The letter must do justice to your feelings and be easy to read. It should become a valuable document.  (…) |
| ***Patient task:*** assessment T4^n^, read letter C4, choose addressee of letter, plan writing letter P5^o^ |
| ***Counselor task:*** write letter C5^p^ (instruction for letter P5). C5 is identical for both treatment conditions. |
| *Example excerpt from C5 (example: addressee to a trusted person):*  (…)  You have written that you would like to address a letter to a trusted person. You can use the following questions and notes to help you write this letter:  Begin the letter with a brief superficial description of the event so that the recipient can get an idea of what actually happened. While doing this, always think about what moments were most important to you: What things do you find so important that you want to tell others about them?  How have you judged the way you acted in the past? How do you feel about it today?  What is the significance of the event in your life, now and in the future?  When you look at everything together, what is the most important conclusion you can draw from what happened at this moment?  (…) |
| ***Patient task:*** read letter C5, write letter P5, plan writing letter P6^q^ |
| ***Counselor task:*** write letter C6^r^ (feedback to letter P5, instruction for letter P6). C6 is identical for both treatment conditions. |
| *Example excerpt from C6:*  (…)  Your letter is a good start regarding the final version <moderate if the letter still needs a lot of revision, e.g. you have put a lot of effort into writing a good letter so far>. There are still some points you could pay attention to when writing your final letter  < insert/combine, e.g.>:  - swear words  - reproachful tone  - linguistic errors  - letter form (introduction, conclusion)  - what is expected by the recipient? Remember that no specific actions should be expected, but that the recipient should be made aware of that ...  - Describe facts, without (self-)accusations  - Distribute attention to the present, past and future: not too much past?  - what were the main themes of the previous texts?>  (…)  The goal is to create a letter you can be proud of. A letter in which you pay attention to both the past, the present and the future.  (…) |
| ***Patient task:*** read letter C6, write letter P6 |
| ***Counselor task:*** write letter C7^s^ (feedback to letter P6, goodbye words). C7 is identical for both treatment conditions. |
| *Example excerpt from C7:*  (…)  A very big compliment for the way you wrote your letter. It is a worthy and beautiful letter. A letter to read over and over again. To read it again and again might be a good idea especially in the future, in moments when old feelings come to the surface again.  (…)  If you wish, you can write something in it to say goodbye to Ilajnafsy and to me as your counselor.  (…) |
| ***Patient task:*** read letter C7, optional: write goodbye letter, assessment T5^t^ |

^a^EXPO: exposure treatment.

^b^CR: cognitive restructuring treatment.

^c^Examples freely translated from Arabic to English. Letters, particularly the feedbacks, are tailored to the patients’ needs.

^d^C1: counselor letter 1.

^e^T2: assessment immediately before starting treatment.

^f^P1: patient letter 1.

^g^P2: patient letter 2.

^h^C2: counselor letter 2.

^i^P3: patient letter 3.

^j^P4: patient letter 4.

^k^C3: counselor letter 3.

^l^T3: assessment after 2 letters.

^m^C4: counselor letter 4.

^n^T4: assessment after 4 letters.

^o^P5: patient letter 5.

^p^C5: counselor letter 5.

^q^P6: patient letter 6.

^r^C6: counselor letter 6.

^s^C7: counselor letter 7.

^t^T5: postassessment time point.

**Changes in primary outcome (completer sample)**

***Within-group changes***

For both treatment conditions, no significant changes in overall posttraumatic stress symptom severity emerged between baseline and T2 (directly before starting treatment) as well as T3 (after 2 letters). Significant changes in overall posttraumatic stress symptom severity were found between baseline and T4 (after 4 letters) as well as between baseline and postassessment time point in both treatment conditions (CR treatment: *d=*−0.48; exposure treatment: *d=*−0. 51 and CR treatment: *d=*−1.13; exposure treatment: *d=*−1.21, respectively).

With regard to the subscale scores, significant changes between baseline and T3 (after 2 letters) emerged in the CR treatment on the “re-experiencing” subscale (*d=*−0.40). Concerning results between baseline and T4 (after 4 letters), changes on the subscale “negative alterations in cognitions and mood” were found to be statistically significant in both treatment conditions (CR treatment: *d=*−0.47; exposure treatment: *d=*−0.43), on the “hyperarousal” subscale significant effects emerged only in the exposure treatment (*d=*−0.53) and on the “re-experiencing” subscale significant effects emerged only in the CR treatment (*d=*−0.55).

For both treatment conditions, changes from baseline to postassessment time point were statistically significant for all subscales, effect sizes ranged from *d=*−0.67 (CR treatment; “avoidance” subscale) to *d=*−1.14 (exposure treatment; “negative alterations in cognitions and mood” subscale).

Participants in the waitlist control group showed statistically significant changes in overall posttraumatic stress symptom severity between baseline and T4 (after 2 weeks of waiting) and between baseline and postassessment time point (*d=*−0.35, and *d=*−0.32, respectively).

Additionally, the waitlist control group participants showed significant changes on the “re-experiencing” subscale between baseline and T3 (after 1 week of waiting) and baseline and T4 (after 2 weeks of waiting) as well as baseline and postassessment time point (all *d=*−0.41). For the “negative alterations in cognitions and mood” subscale, significant changes emerged between baseline and T4 (after 2 weeks of waiting) (*d=*−0.34). Other changes within the waitlist control group were nonsignificant after Bonferroni correction. Table S2 provides further information on estimates for within-group changes in posttraumatic stress symptom severity between baseline and each subsequent measurement time point in the completer sample.

**Table S2.** Estimated within-group changes in posttraumatic stress symptom severity between baseline and subsequent assessments (completer sample)^a^.

|  | | | | | | | | | | | | | | | | | |
| --- | --- | --- | --- | --- | --- | --- | --- | --- | --- | --- | --- | --- | --- | --- | --- | --- | --- |
| Outcome (PCL-5^b^) and group | | T2^c^ – T1^d^ | | | | T3^e^ – T1 | | | | T4^f^ – T1 | | | | T5^g^ – T1 | | | |
|  | | M^h^ (SE) | 95% CI | *P* value | *d* | M (SE) | 95% CI | *P* value | *d* | M (SE) | 95% CI | *P* value | *d* | M (SE) | 95% CI | *P* value | *d* |
|  | | | | | | | | | | | | | | | | | |
| **Overall** | | | | | | | | | | | | | | | | | |
|  | CR^i^ | −0.58 (1.37) | −3.27 to 2.11 | .67 | −0.05 | −1.80 (1.35) | −4.44 to 0.85 | .18 | −0.17 | −6.60 (1.74) | −10.01 to −3.18 | *<.001*^j^ | −0.48 | −20.13 (2.30) | −24.64 to −15.62 | *<.001* | −1.13 |
|  | EXPO^k^ | −0.10 (1.16) | −2.37 to 2.18 | .93 | −0.01 | −2.51 (1.42) | −5.29 to 0.26 | .08 | −0.24 | −6.05 (1.55) | −9.08 to −3.01 | *<.001* | −0.51 | −17.96 (1.96) | −21.80 to −14.13 | *<.001* | −1.21 |
|  | WAIT^l^ | −1.77 (1.08) | −3.90 to 0.35 | .10 | −0.16 | −3.10 (1.12) | −5.30 to −0.90 | .006 | −0.28 | −4.24 (1.26) | −6.70 to −1.77 | *.001* | −0.35 | −3.82 (1.19) | −6.16 to −1.49 | *.001* | −0.32 |
| **Re-experiencing** | | | | | | | | | | | | | | | | | |
|  | CR | −0.75 (0.43) | −1.59 to 0.09 | .08 | −0.22 | −1.42 (0.47) | −2.35 to −0.49 | *.003* | −0.40 | −2.33 (0.54) | −3.38 to −1.28 | *<.001* | −0.55 | −5.45 (0.69) | −6.79 to −4.10 | *<.001* | −1.03 |
|  | EXPO | 0.11 (0.44) | −0.75 to 0.97 | .81 | 0.03 | −0.39 (0.49) | −1.35 to 0.56 | .42 | −0.11 | −1.33 (0.50) | −2.32 to −0.35 | .008 | −0.35 | −4.13 (0.62) | −5.36 to −2.91 | *<.001* | −0.87 |
|  | WAIT | −0.97 (0.40) | −1.75 to −0.20 | .01 | −0.25 | −1.68 (0.42) | −2.50 to −0.87 | *<.001* | −0.41 | −1.78 (0.45) | −2.65 to −0.90 | *<.001* | −0.41 | −1.81 (0.43) | −2.66 to −0.96 | *<.001* | −0.41 |
| **Avoidance** | | | | | | | | | | | | | | | | | |
|  | CR | −0.01 (0.30) | −0.59 to 0.58 | .98 | −0.00 | 0.18 (0.32) | −0.44 to 0.80 | .57 | 0.07 | −0.02 (0.29) | −0.60 to 0.55 | .94 | −0.01 | −1.90 (0.37) | −2.62 to −1.18 | *<.001* | −0.67 |
|  | EXPO | 0.16 (0.28) | −0.38 to 0.70 | .56 | 0.08 | 0.11 (0.33) | −0.54 to 0.75 | .75 | 0.04 | −0.35 (0.33) | −0.98 to 0.29 | .29 | −0.14 | −1.99 (0­­.36) | −2.71 to −1.28 | *<.001* | −0.72 |
|  | WAIT | 0.17 (0.23) | −0.27 to 0.62 | .45 | 0.08 | 0.01 (0.23) | −0.45 to 0.47 | .98 | 0.00 | −0.06 (0.22) | −0.49 to 0.38 | .80 | −0.03 | 0.13 (0.22) | −0.30 to 0.56 | .56 | 0.06 |
| **Negative alterations in cognitions and mood** | | | | | | | | | | | | | | | | | |
|  | CR | 0.11 (0.60) | −1.06 to 1.28 | .85 | 0.02 | −0.29 (0.57) | −1.41 to 0.82 | .60 | −0.07 | −2.64 (0.71) | −4.04 to −1.24 | *<.001* | −0.47 | −7.68 (0.96) | −9.57 to −5.80 | *<.001* | −1.04 |
|  | EXPO | −0.24 (0.65) | −1.51 to 1.04 | .72 | −0.05 | −1.15 (0.71) | −2.55 to 0.24 | .11 | −0.22 | −2.46 (0.75) | −3.93 to −0.98 | *.001* | −0.43 | −7.08 (0.82) | −8.69 to −5.48 | *<.001* | −1.14 |
|  | WAIT | −0.50 (0.44) | −1.36 to 0.36 | .26 | −0.11 | −0.85 (0.47) | −1.78 to 0.08 | .07 | −0.18 | −1.64 (0.50) | −2.62 to −0.66 | *.001* | −0.34 | −1.19 (0.50) | −2.17 to −0.21 | .02 | −0.24 |
| **Hyperarousal** | | | | | | | | | | | | | | | | | |
|  | CR | 0.06 (0.55) | −1.02 to 1.15 | .91 | 0.01 | −0.26 (0.54) | −1.31 to 0.80 | .63 | −0.06 | −1.61 (0.64) | −2.86 to −0.36 | .01 | −0.32 | −5.10 (0.73) | −6.52 to −3.68 | *<.001* | −0.92 |
|  | EXPO | −0.13 (0.39) | −0.89 to 0.63 | .74 | −0.04 | −1.07 (0.47) | −2.00 to −0.14 | .02 | −0.32 | −1.91 (0.48) | −2.84 to −0.98 | *<.001* | −0.53 | −4.76 (0.63) | −5.99 to −3.52 | *<.001* | −0.99 |
|  | WAIT | −0.47 (0.41) | −1.27 to 0.32 | .24 | −0.12 | −0.57 (0.40) | −1.36 to 0.22 | .16 | −0.14 | −0.76 (0.47) | −1.68 to 0.16 | .11 | −0.17 | −0.95 (0.41) | −1.75 to −0.15 | .02 | −0.23 |

^a^All estimates were pooled across 100 imputed data sets.

^b^PCL-5: Posttraumatic Stress Disorder Checklist for the Diagnostic and Statistical Manual of Mental Disorders, Fifth Edition.

^c^T2: assessment immediately before starting treatment or waiting time.

^d^T1: baseline assessment.

^e^T3: assessment after 2 letters or 1 week of waiting.

^f^T4: assessment after 4 letters or 2 weeks of waiting.

^g^T5: postassessment time point.

^h^M: change score (mean change in raw score units of the questionnaire).

^i^CR: cognitive restructuring treatment.

^j^Significant *P* values.

^k^EXPO: exposure treatment.

^l^WAIT: waitlist control group.

***Between-group differences***

There were no statistically significant differences between the two treatment conditions regarding the magnitude of change in posttraumatic stress symptom severity (overall or subscales) at any measurement time point. Differences between the CR treatment and the waitlist control group as well as between the exposure treatment and the waitlist control group regarding the magnitude of change in symptom severity (overall or subscales) only reached significance between baseline and postassessment time point. Effect sizes for the overall posttraumatic stress symptom severity were high between the CR treatment and the waitlist control group (*d=*1.08) and between the exposure treatment and the waitlist control group (*d=*1.05). Effect sizes for the subscales ranged from *d=*0.51 (exposure treatment vs. waitlist control group; “re-experiencing” subscale) to *d=*1.04 (CR treatment /exposure treatment vs. waitlist control group; “negative alterations in cognition and mood” subscale). Table S3 presents further details on estimated between-group differences in mean change scores and effect sizes with regard to posttraumatic stress symptom severity between baseline and each subsequent measurement time point for the completer sample.

**Table S3.** Estimated between-group differences in posttraumatic stress symptom severity between baseline and subsequent assessments (completer sample)^a^.

| Outcome (PCL-5^b^) and group comparison | | T2^c^ – T1^d^ | | | | T3^e^ – T1 | | | | T4^f^ – T1 | | | | T5^g^ – T1 | | | |
| --- | --- | --- | --- | --- | --- | --- | --- | --- | --- | --- | --- | --- | --- | --- | --- | --- | --- |
|  | | ΔM^h^ (SE) | 95% CI | *P* value | *d* | ΔM (SE) | 95% CI | *P* value | *d* | ΔM (SE) | 95% CI | *P* value | *d* | ΔM (SE) | 95% CI | *P* value | *d* |
|  | | | | | | | | | | | | | | | | | |
| **Overall** | | | | | | | | | | | | | | | | | |
|  | CR^i^ vs. EXPO^j^ | 0.48 (1.80) | −3.04 to 4.01 | .79 | 0.05 | −0.72 (1.97) | −4.57 to 3.14 | .72 | −0.07 | 0.55 (2.33) | −4.03 to 5.13 | .81 | 0.04 | 2.17 (3.03) | −3.76 to 8.10 | .47 | 0.13 |
|  | EXPO vs. WAIT^k^ | −1.68 (1.59) | −4.79 to 1.44 | .29 | −0.17 | −0.59 (1.80) | −4.11 to 2.94 | .74 | −0.06 | 1.81 (1.99) | −2.10 to 5.72 | .36 | 0.15 | 14.14 (2.29) | 9.65 to 18.63 | *<.001*^l^ | 1.05 |
|  | CR vs. WAIT | −1.19 (1.75) | −4.62 to 2.24 | .50 | −0.11 | −1.30 (1.76) | −4.75 to 2.14 | .46 | −0.12 | 2.36 (2.15) | −1.85 to 6.58 | .27 | 0.18 | 16.31 (2.59) | 11.23 to 21.39 | *<.001* | 1.08 |
| **Re-experiencing** | | | | | | | | | | | | | | | | | |
|  | CR vs. EXPO | 0.86 (0.61) | −0.35 to 2.06 | .16 | 0.26 | 1.03 (0.68) | −0.31 to 2.36 | .13 | 0.29 | 0.99 (0.73) | −0.45 to 2.44 | .18 | 0.25 | 1.32 (0.93) | −0.50 to 3.13 | .16 | 0.26 |
|  | EXPO vs. WAIT | −1.08 (0.59) | −2.24 to 0.08 | .07 | −0.29 | −1.29 (0.64) | −2.54 to −0.03 | .04 | −0.33 | −0.44 (0.67) | −1.76 to 0.88 | .51 | −0.11 | 2.32 (0.76) | 0.83 to 3.81 | *.002* | 0.51 |
|  | CR vs. WAIT | −0.22 (0.58) | −1.37 to 0.92 | .70 | −0.06 | −0.26 (0.63) | −1.49 to 0.97 | .68 | −0.07 | 0.55 (0.70) | −0.81 to 1.92 | .43 | 0.13 | 3.63 (0.81) | 2.04 to 5.23 | *<.001* | 0.75 |
| **Avoidance** | | | | | | | | | | | | | | | | | |
|  | CR vs. EXPO | 0.17 (0.41) | −0.63 to 0.96 | .68 | 0.07 | −0.07 (0.46) | −0.97 to 0.82 | .87 | −0.03 | −0.33 (0.44) | −1.19 to 0.53 | .46 | −0.14 | −0.09 (0.52) | −1.11 to 0.92 | .86 | −0.03 |
|  | EXPO vs. WAIT | 0.01 (0.36) | −0.69 to 0.71 | .98 | 0.01 | −0.10 (0.41) | −0.89 to 0.70 | .81 | −0.04 | 0.29 (0.39) | −0.48 to 1.06 | .46 | 0.13 | 2.12 (0.43) | 1.29 to 2.95 | *<.001* | 0.85 |
|  | CR vs. WAIT | 0.18 (0.38) | −0.56 to 0.91 | .64 | 0.08 | −0.17 (0.40) | −0.95 to 0.60 | .66 | −0.07 | −0.03 (0.37) | −0.76 to 0.69 | .93 | −0.01 | 2.03 (0.43) | 1.19 to 2.87 | *<.001* | 0.80 |
| **Negative alterations in cognitions and mood** | | | | | | | | | | | | | | | | | |
|  | CR vs. EXPO | −0.35 (0.89) | −2.08 to 1.39 | .70 | −0.07 | −0.86 (0.91) | −2.64 to 0.93 | .35 | −0.18 | 0.18 (1.04) | −1.85 to 2.21 | .86 | 0.03 | 0.60 (1.26) | −1.88 to 3.08 | .64 | 0.09 |
|  | EXPO vs. WAIT | −0.26 (0.79) | −1.80 to 1.28 | .74 | −0.06 | 0.30 (0.85) | −1.37 to 1.97 | .73 | 0.06 | 0.81 (0.91) | −0.96 to 2.59 | .37 | 0.15 | 5.90 (0.96) | 4.02 to 7.77 | *<.001* | 1.04 |
|  | CR vs. WAIT | −0.61 (0.74) | −2.06 to 0.85 | .41 | −0.13 | −0.56 (0.74) | −2.02 to 0.90 | .45 | −0.12 | 1.00 (0.87) | −0.71 to 2.71 | .25 | 0.19 | 6.50 (1.08) | 4.37 to 8.62 | *<.001* | 1.04 |
| **Hyperarousal** | | | | | | | | | | | | | | | | | |
|  | CR vs. EXPO | −0.19 (0.68) | −1.52 to 1.13 | .78 | −0.05 | −0.81 (0.72) | −2.23 to 0.60 | .26 | −0.21 | −0.30 (0.80) | −1.87 to 1.26 | .71 | −0.07 | 0.34 (0.97) | −1.55 to 2.24 | .72 | 0.07 |
|  | EXPO vs. WAIT | −0.35 (0.56) | −1.45 to 0.75 | .54 | −0.10 | 0.50 (0.62) | −0.72 to 1.72 | .42 | 0.14 | 1.15 (0.67) | −0.16 to 2.46 | .09 | 0.29 | 3.81 (0.75) | 2.33 to 5.28 | *<.001* | 0.85 |
|  | CR vs. WAIT | −0.54 (0.69) | −1.88 to 0.81 | .43 | −0.13 | −0.31 (0.67) | −1.63 to 1.01 | .64 | −0.08 | 0.85 (0.79) | −0.71 to 2.40 | .29 | 0.18 | 4.15 (0.83) | 2.52 to 5.78 | *<.001* | 0.85 |

^a^All estimates were pooled across 100 imputed data sets.

^b^PCL-5: Posttraumatic Stress Disorder Checklist for the Diagnostic and Statistical Manual of Mental Disorders, Fifth Edition.

^c^T2: assessment immediately before starting treatment or waiting time.

^d^T1: baseline assessment.

^e^T3: assessment after 2 letters or 1 week of waiting.

^f^T4: assessment after 4 letters or 2 weeks of waiting.

^g^T5: postassessment time point.

^h^ΔM: difference between group-specific means of change scores.

^i^CR: cognitive restructuring treatment.

^j^EXPO: exposure treatment.

^k^WAIT: waitlist control group.

^l^Significant *P* values.

***Reliable change, remission, and RCSI***

Table S4 summarizes rates of reliable change, remission, and RCSI in all three conditions in the completer sample. The two treatment conditions did not significantly differ regarding the rates of participants who experienced reliable change (*P*=.87), remission (*P*=.57), or RCSI (*P*=.81) between baseline and postassessment time point.

Rates of reliable change (*P*<.001), remission (*P*<.001), and RCSI (*P*<.001) differed significantly between the CR treatment and the waitlist control group. Similarly, the exposure treatment and the waitlist control group differed significantly with regard to rates of reliable change (*P*<.001), remission (*P*<.001), and RCSI (*P*<.001).

**Table S4.** Rates of reliable change, remission, and reliable change and significant improvement (RCSI;completer sample)^a^.

|  | | Baseline assessment to postassessment time point (PCL-5^b^), n (%) | | |
| --- | --- | --- | --- | --- |
|  | | CR^c^ (n=64) | EXPO^d^ (n=59) | WAIT^e^ (n=101) |
|  | | | | |
| **Reliable change** | | | | |
|  | Improvement | 35.79 (55.9) | 35.69 (60.5) | 18 (17.8) |
|  | No change | 26.94 (42.1) | 22.22 (37.7) | 76 (75.2) |
|  | Deterioration | 1.27 (2) | 1.09 (1.8) | 7 (6.9) |
| **Remission** | | 31.32 (48.9) | 25.8 (43.7) | 4 (4) |
| **RCSI** | | 27.81 (43.5) | 24.66 (41.8) | 3 (3) |

^a^All results were averaged across imputed data sets; therefore, the counts contain decimals.

^b^PCL-5: Posttraumatic Stress Disorder Checklist for the Diagnostic and Statistical Manual of Mental Disorders, Fifth Edition. An increase or decrease of 16 PCL-5 points between the baseline and postassessment time point was defined as reliable deterioration or improvement, respectively. Remission was defined as a score of ≥23 at baseline and a score of <23 at the postassessment time point. RCSI was defined as experiencing both remission and reliable improvement from baseline to the postassessment time point.

^c^CR: cognitive restructuring treatment.

^d^EXPO: exposure treatment.

^e^WAIT: waitlist control group.

**Changes in secondary outcomes (completer sample)**

***Within-group changes***

In both the CR treatment and exposure treatment, all changes in secondary outcome measures between baseline and postassessment time point were significant. Effect size estimates ranged from *d=*−0.41 (exposure treatment; trauma-related guilt) to *d=*−1.19 (CR treatment; depressive symptom severity). In the waitlist control group, levels of depressive and anxiety symptoms as well as quality of life also changed significantly (*d=*−0.49, *d=*−0.48, and *d=*0.38, respectively). Other changes were nonsignificant. Estimated within-group changes for all secondary outcomes between baseline and postassessment time point in the completer sample can be found in table S5.

***Between-group differences***

The results revealed no evidence of differences in the magnitude of change for any secondary outcome measure between the two treatment conditions. Significant differences in the magnitude of change between the exposure treatment and the waitlist control group and between the CR treatment and the waitlist control group in the magnitudes of change between baseline and postassessment time point were found for all secondary outcome measures with effect sizes ranging from *d=*0.49 (exposure treatment versus waitlist control group; anxiety symptom severity) to *d=*0.96 (CR treatment versus waitlist control group; depressive symptom severity). Estimated between-group differences between baseline and postassessment time point for all secondary outcomes in the completer sample can be found in table S5.

**Table S5.** Estimated within-group changes and between-group differences for secondary outcomes between baseline and postassessment time point (completer sample)^a^.

| Outcome and group | | Within-group changes | | | | Group comparison | Between-group differences | | | |
| --- | --- | --- | --- | --- | --- | --- | --- | --- | --- | --- |
|  | | M^b^ (SE) | 95% CI | *P* value | *d* |  | ΔM^c^ (SE) | 95% CI | *P* value | *d* |
|  | | | | | | | | | | |
| **Posttraumatic maladaptive beliefs (PMBS^d^)** | | | | | | | | | | |
|  | CR^e^ | −13.47 (2.48) | −18.34 to −8.60 | *<.001*^f^ | −0.69 | CR vs. EXPO^g^ | 1.41 (3.13) | −4.73 to 7.54 | .65 | 0.08 |
|  | EXPO | −12.07 (1.90) | −15.80 to −8.34 | *<.001* | −0.83 | EXPO vs. WAIT^h^ | 11.30 (2.20) | 6.99 to 15.6 | *<.001* | 0.88 |
|  | WAIT | −0.77 (1.10) | −2.92 to 1.38 | .48 | −0.07 | CR vs. WAIT | 12.70 (2.72) | 7.38 to 18.02 | *<.001* | 0.81 |
| **Trauma-related guilt (TRGI^i^)** | | | | | | | | | | |
|  | CR | −0.44 (0.09) | −0.62 to −0.26 | *<.001* | −0.60 | CR vs. EXPO | −0.07 (0.14) | −0.34 to 0.21 | .63 | −0.09 |
|  | EXPO | −0.51 (0.11) | −0.72 to −0.30 | *<.001* | −0.41 | EXPO vs. WAIT | 0.41 (0.12) | 0.17 to 0.65 | *.001* | 0.58 |
|  | WAIT | −0.10 (0.06) | −0.21 to 0.02 | .10 | −0.18 | CR vs. WAIT | 0.34 (0.11) | 0.13 to 0.56 | *.002* | 0.54 |
| **Anxiety symptom severity (GAD-7^j^)** | | | | | | | | | | |
|  | CR | −5.51 (0.71) | −6.90 to −4.13 | *<.001* | −1.01 | CR vs. EXPO | 0.87 (1.03) | −1.15 to 2.88 | .40 | 0.15 |
|  | EXPO | −4.64 (0.74) | −6.10 to −3.18 | *<.001* | −0.81 | EXPO vs. WAIT | 2.50 (0.87) | 0.80 to 4.21 | *.004* | 0.49 |
|  | WAIT | −2.14 (0.45) | −3.02 to −1.26 | *<.001* | −0.48 | CR vs. WAIT | 3.37 (0.84) | 1.73 to 5.01 | *<.001* | 0.68 |
| **Depressive symptom severity (PHQ-9^k^)** | | | | | | | | | | |
|  | CR | −8.00 (0.87) | −9.71 to −6.29 | *<.001* | −1.19 | CR vs. EXPO | 1.42 (1.22) | −0.97 to 3.81 | .24 | 0.21 |
|  | EXPO | −6.58 (0.85) | −8.25 to −4.90 | *<.001* | −1.00 | EXPO vs. WAIT | 4.20 (0.98) | 2.28 to 6.12 | *<.001* | 0.73 |
|  | WAIT | −2.38 (0.48) | −3.32 to −1.43 | *<.001* | −0.49 | CR vs. WAIT | 5.62 (1.00) | 3.67 to 7.58 | *<.001* | 0.96 |
| **Somatoform symptom severity (PHQ-15^l^)** | | | | | | | | | | |
|  | CR | −3.17 (0.64) | −4.42 to −1.93 | *<.001* | −0.64 | CR vs. EXPO | 0.63 (0.92) | −1.16 to 2.43 | .49 | 0.13 |
|  | EXPO | −2.54 (0.66) | −3.83 to −1.25 | *<.001* | −0.50 | EXPO vs. WAIT | 2.36 (0.77) | 0.86 to 3.87 | *.002* | 0.52 |
|  | WAIT | −0.18 (0.40) | −0.96 to 0.60 | .65 | −0.04 | CR vs. WAIT | 3.00 (0.75) | 1.52 to 4.47 | *<.001* | 0.67 |
| **Quality of life (EUROHIS-QOL-8^m^)** | | | | | | | | | | |
|  | CR | 5.33 (0.77) | 3.82 to 6.83 | *<.001* | 0.91 | CR vs. EXPO | −0.92 (1.05) | −2.98 to 1.14 | .38 | −0.16 |
|  | EXPO | 4.41 (0.71) | 3.01 to 5.81 | *<.001* | 0.80 | EXPO vs. WAIT | −2.92 (0.81) | −4.52 to −1.33 | *<.001* | −0.61 |
|  | WAIT | 1.49 (0.39) | 0.72 to 2.25 | *<.001* | 0.38 | CR vs. WAIT | −3.84 (0.86) | −5.53 to −2.15 | *<.001* | −0.78 |

^a^All estimates were pooled across 100 imputed data sets.

^b^M: change score (mean change in raw score units of the questionnaire).

^c^ΔM: difference between group-specific means of change scores.

^d^PMBS: Posttraumatic Maladaptive Beliefs Scale.

^e^CR: cognitive restructuring treatment.

^f^Significant *P* values.

^g^EXPO: exposure treatment.

^h^WAIT: waitlist control group.

^i^TRGI: Trauma-Related Guilt Inventory.

^j^GAD-7: Generalized Anxiety Disorder–7.

^k^PHQ-9: Patient Health Questionnaire–9.

^l^PHQ-15: Patient Health Questionnaire–15.

^m^EUROHIS-QOL-8: EUROHIS Quality of Life 8-item index.

**Sensitivity analysis**

**Table S6.** Estimated within-group changes in posttraumatic stress symptom severity between baseline and subsequent assessments under various missing not at random conditions (25%, 50% and 75% increase of individual imputed values) in the intention-to-treat sample ^a^.

| Outcome (PCL-5^b^), group and condition | | | T2^c^ – T1^d^ | | | | T3^e^ – T1 | | | | T4^f^ – T1 | | | | | T5^g^ – T1 | | | |
| --- | --- | --- | --- | --- | --- | --- | --- | --- | --- | --- | --- | --- | --- | --- | --- | --- | --- | --- | --- |
|  | | | M^h^ (SE) | 95% CI | *P* value | *d* | M (SE) | 95% CI | *P* value | *d* | M (SE) | 95% CI | *P* value | | *d* | M (SE) | 95% CI | *P* value | *d* |
|  | | | | | | | | | | | | | | | | | | | |
| **Overall** | | | | | | | | | | | | | | | | | | | |
|  | CR^i^ | 25% | −0.50 (1.08) | −2.62 to 1.62 | .64 | −0.04 | 1.73 (1.36) | −0.93 to 4.39 | .20 | 0.13 | −3.40 (1.72) | −6.76 to −0.04 | | .05 | −0.21 | −15.91 (2.48) | −20.78 to −11.05 | *<.001*^j^ | −0.79 |
|  | CR | 50% | 0.06 (1.21) | −2.31 to 2.42 | .96 | 0.00 | 5.99 (1.71) | 2.64 to 9.34 | *<.001* | 0.36 | −0.03 (2.00) | −3.95 to 3.89 | | .99 | −0.00 | −12.72 (2.86) | −18.31 to −7.12 | *<.001* | −0.57 |
|  | CR | 75% | 0.62 (1.36) | −2.05 to 3.28 | .65 | 0.04 | 10.24 (2.14) | 6.05 to 14.44 | *<.001* | 0.48 | 3.35 (2.35) | −1.27 to 7.96 | | .16 | 0.15 | −9.52 (3.27) | −15.92 to −3.12 | *.004* | −0.38 |
|  | EXPO^k^ | 25% | −0.70 (1.09) | −2.84 to 1.44 | .52 | −0.06 | 0.24 (1.65) | −3.00 to 3.48 | .88 | 0.01 | −4.66 (1.63) | −7.85 to −1.47 | | *.004* | −0.30 | −13.68 (2.17) | −17.94 to −9.42 | *<.001* | −0.75 |
|  | EXPO | 50% | 0.15 (1.20) | −2.20 to 2.50 | .90 | 0.01 | 4.29 (1.98) | 0.41 to 8.17 | .03 | 0.23 | −1.56 (1.88) | −5.24 to 2.12 | | .41 | −0.09 | −10.36 (2.50) | −15.26 to −5.45 | *<.001* | −0.51 |
|  | EXPO | 75% | 0.99 (1.36) | −1.67 to 3.66 | .47 | 0.07 | 8.35 (2.38) | 3.68 to 13.01 | *<.001* | 0.36 | 1.54 (2.20) | −2.78 to 5.85 | | .49 | 0.07 | −7.04 (2.87) | −12.67 to −1.40 | .01 | −0.31 |
|  | WAIT^l^ | 25% | −1.11 (1.17) | −3.40 to 1.18 | .34 | −0.09 | −0.73 (1.21) | −3.11 to 1.64 | .55 | −0.06 | −0.67 (1.41) | −3.44 to 2.10 | | .63 | −0.05 | −1.71 (1.31) | −4.27 to 0.85 | .19 | −0.13 |
|  | WAIT | 50% | −0.11 (1.34) | −2.74 to 2.53 | .94 | −0.01 | 1.80 (1.46) | −1.06 to 4.67 | .22 | 0.12 | 2.93 (1.71) | −0.41 to 6.27 | | .09 | 0.18 | 0.37 (1.54) | −2.64 to 3.39 | .81 | 0.02 |
|  | WAIT | 75% | 0.89 (1.56) | −2.16 to 3.95 | .57 | 0.05 | 4.34 (1.78) | 0.84 to 7.84 | .02 | 0.23 | 6.53 (2.08) | 2.47 to 10.60 | | *.002* | 0.32 | 2.45 (1.83) | −1.14 to 6.04 | .18 | 0.13 |
| **Re-experiencing** | | | | | | | | | | | | | | | | | | | |
|  | CR | 25% | −0.78 (0.37) | −1.50 to −0.06 | .04 | −0.20 | −0.66 (0.49) | −1.62 to 0.31 | .18 | −0.15 | −1.52 (0.52) | −2.54 to −0.51 | | *.003* | −0.32 | −4.77 (0.71) | −6.16 to −3.38 | *<.001* | −0.79 |
|  | CR | 50% | −0.67 (0.39) | −1.43 to 0.09 | .09 | −0.17 | 0.21 (0.57) | −0.92 to 1.34 | .72 | 0.04 | −0.81 (0.59) | −1.96 to 0.34 | | .17 | −0.15 | −4.18 (0.80) | −5.75 to −2.62 | *<.001* | −0.64 |
|  | CR | 75% | −0.56 (0.41) | −1.37 to 0.25 | .18 | −0.14 | 1.08 (0.67) | −0.24 to 2.40 | .11 | 0.18 | −0.10 (0.67) | −1.42 to 1.22 | | .88 | −0.02 | −3.60 (0.90) | −5.35 to −1.84 | *<.001* | −0.51 |
|  | EXPO | 25% | −0.28 (0.34) | −0.94 to 0.38 | .41 | −0.08 | 0.11 (0.50) | −0.87 to 1.09 | .82 | 0.02 | −1.18 (0.50) | −2.15 to −0.20 | | .02 | −0.26 | −3.24 (0.70) | −4.62 to −1.86 | *<.001* | −0.56 |
|  | EXPO | 50% | −0.09 (0.36) | −0.81 to 0.62 | .80 | −0.03 | 1.05 (0.58) | −0.08 to 2.19 | .07 | 0.20 | −0.47 (0.56) | −1.57 to 0.64 | | .41 | −0.09 | −2.49 (0.81) | −4.07 to −0.90 | *.002* | −0.39 |
|  | EXPO | 75% | 0.10 (0.40) | −0.69 to 0.88 | .81 | 0.02 | 2.00 (0.68) | 0.67 to 3.32 | *.003* | 0.32 | 0.24 (0.65) | −1.03 to 1.51 | | .71 | 0.04 | −1.74 (0.92) | −3.54 to 0.07 | .06 | −0.25 |
|  | WAIT | 25% | −0.95 (0.41) | −1.75 to −0.15 | .02 | −0.22 | −1.26 (0.45) | −2.14 to −0.39 | .005 | −0.29 | −1.07 (0.51) | −2.06 to −0.07 | | .04 | −0.23 | −1.32 (0.47) | −2.23 to −0.41 | .005 | −0.28 |
|  | WAIT | 50% | −0.72 (0.45) | −1.60 to 0.15 | .10 | −0.16 | −0.73 (0.50) | −1.72 to 0.25 | .14 | −0.15 | −0.27 (0.59) | −1.43 to 0.89 | | .64 | −0.05 | −0.82 (0.53) | −1.85 to 0.21 | .12 | −0.16 |
|  | WAIT | 75% | −0.50 (0.49) | −1.46 to 0.46 | .31 | −0.10 | −0.20 (0.57) | −1.32 to 0.92 | .73 | −0.04 | 0.52 (0.69) | −0.82 to 1.86 | | .45 | 0.08 | −0.32 (0.60) | −1.50 to 0.85 | .59 | −0.06 |
| **Avoidance** | | | | | | | | | | | | | | | | | | | |
|  | CR | 25% | 0.11 (0.23) | −0.34 to 0.56 | .62 | 0.05 | 0.70 (0.28) | 0.14 to 1.25 | .02 | 0.25 | 0.45 (0.27) | −0.09 to 0.99 | | .10 | 0.18 | −1.27 (0.38) | −2.02 to −0.52 | *.001* | −0.41 |
|  | CR | 50% | 0.17 (0.24) | −0.30 to 0.64 | .48 | 0.07 | 1.19 (0.33) | 0.56 to 1.83 | *<.001* | 0.39 | 0.88 (0.31) | 0.27 to 1.49 | | *.004* | 0.32 | −0.87 (0.43) | −1.72 to −0.02 | .04 | −0.26 |
|  | CR | 75% | 0.22 (0.25) | −0.27 to 0.72 | .38 | 0.09 | 1.69 (0.37) | 0.96 to 2.43 | *<.001* | 0.48 | 1.32 (0.36) | 0.62 to 2.02 | | *<.001* | 0.42 | −0.48 (0.49) | −1.45 to 0.49 | .33 | −0.13 |
|  | EXPO | 25% | 0.12 (0.21) | −0.29 to 0.53 | .57 | 0.06 | 0.34 (0.31) | −0.27 to 0.96 | .27 | 0.12 | −0.13 (0.32) | −0.76 to 0.49 | | .68 | −0.05 | −1.48 (0.37) | −2.21 to −0.75 | **<***.001* | −0.46 |
|  | EXPO | 50% | 0.21 (0.22) | −0.22 to 0.64 | .34 | 0.09 | 0.81 (0.36) | 0.10 to 1.52 | .03 | 0.25 | 0.23 (0.36) | −0.48 to 0.93 | | .53 | 0.07 | −1.11 (0.42) | −1.94 to −0.28 | .01 | −0.31 |
|  | EXPO | 75% | 0.30 (0.23) | −0.16 to 0.76 | .20 | 0.12 | 1.27 (0.42) | 0.46 to 2.09 | *.002* | 0.35 | 0.58 (0.41) | −0.22 to 1.38 | | .16 | 0.17 | −0.74 (0.48) | −1.68 to 0.20 | .12 | −0.19 |
|  | WAIT | 25% | 0.43 (0.23) | −0.02 to 0.88 | .06 | 0.17 | 0.45 (0.25) | −0.04 to 0.93 | .07 | 0.17 | 0.52 (0.24) | 0.04 to 1.00 | | .03 | 0.22 | 0.48 (0.24) | 0.01 to 0.95 | .04 | 0.20 |
|  | WAIT | 50% | 0.54 (0.25) | 0.05 to 1.02 | .03 | 0.20 | 0.73 (0.28) | 0.18 to 1.28 | .01 | 0.25 | 0.93 (0.28) | 0.38 to 1.49 | | *.001* | 0.35 | 0.71 (0.27) | 0.18 to 1.24 | .008 | 0.26 |
|  | WAIT | 75% | 0.64 (0.27) | 0.11 to 1.17 | .02 | 0.23 | 1.01 (0.32) | 0.38 to 1.65 | *.002* | 0.31 | 1.35 (0.33) | 0.71 to 1.99 | | *<.001* | 0.43 | 0.94 (0.31) | 0.35 to 1.54 | *.002* | 0.31 |
| **Negative alterations in cognitions and mood** | | | | | | | | | | | | | | | | | | | |
|  | CR | 25% | 0.14 (0.50) | −0.84 to 1.12 | .78 | 0.03 | 0.97 (0.60) | −0.21 to 2.15 | .12 | 0.17 | −1.52 (0.78) | −3.04 to 0.01 | | .05 | −0.21 | −6.11 (1.12) | −8.30 to −3.91 | *<.001* | −0.69 |
|  | CR | 50% | 0.37 (0.55) | −0.71 to 1.44 | .51 | 0.06 | 2.63 (0.74) | 1.17 to 4.09 | *<.001* | 0.36 | −0.24 (0.90) | −2.00 to 1.52 | | .79 | −0.03 | −4.88 (1.29) | −7.41 to −2.35 | *<.001* | −0.50 |
|  | CR | 75% | 0.59 (0.61) | −0.60 to 1.79 | .33 | 0.09 | 4.29 (0.91) | 2.49 to 6.08 | *<.001* | 0.48 | 1.03 (1.04) | −1.01 to 3.08 | | .32 | 0.10 | −3.65 (1.48) | −6.55 to −0.76 | .01 | −0.33 |
|  | EXPO | 25% | −0.70 (0.56) | −1.80 to 0.40 | .21 | −0.12 | −0.44 (0.74) | −1.89 to 1.02 | .56 | −0.06 | −2.06 (0.74) | −3.51 to −0.61 | | .005 | −0.29 | −5.44 (0.98) | −7.37 to −3.51 | *<.001* | −0.69 |
|  | EXPO | 50% | −0.39 (0.59) | −1.55 to 0.78 | .52 | −0.06 | 1.03 (0.86) | −0.67 to 2.72 | .24 | 0.12 | −0.92 (0.83) | −2.56 to 0.72 | | .27 | −0.12 | −4.23 (1.14) | −6.45 to −2.00 | *<.001* | −0.49 |
|  | EXPO | 75% | −0.08 (0.65) | −1.34 to 1.19 | .91 | −0.01 | 2.49 (1.01) | 0.50 to 4.48 | .01 | 0.26 | 0.22 (0.95) | −1.65 to 2.09 | | .82 | 0.02 | −3.01 (1.30) | −5.56 to −0.47 | .02 | −0.31 |
|  | WAIT | 25% | −0.46 (0.48) | −1.39 to 0.48 | .34 | −0.09 | −0.11 (0.52) | −1.13 to 0.91 | .84 | −0.02 | −0.41 (0.57) | −1.52 to 0.71 | | .48 | −0.08 | −0.51 (0.56) | −1.59 to 0.58 | .36 | −0.09 |
|  | WAIT | 50% | −0.08 (0.54) | −1.14 to 0.98 | .88 | −0.01 | 0.86 (0.62) | −0.35 to 2.07 | .16 | 0.14 | 0.94 (0.68) | −0.39 to 2.26 | | .17 | 0.14 | 0.26 (0.64) | −0.99 to 1.52 | .68 | 0.04 |
|  | WAIT | 75% | 0.29 (0.62) | −0.92 to 1.51 | .63 | 0.04 | 1.83 (0.74) | 0.38 to 3.28 | .01 | 0.24 | 2.28 (0.81) | 0.68 to 3.88 | | .005 | 0.29 | 1.03 (0.75) | −0.44 to 2.50 | .17 | 0.14 |
| **Hyperarousal** | | | | | | | | | | | | | | | | | | | |
|  | CR | 25% | 0.02 (0.43) | −0.82 to 0.86 | .96 | 0.00 | 0.72 (0.52) | −0.30 to 1.75 | .17 | 0.15 | −0.81 (0.64) | −2.06 to 0.44 | | .20 | −0.14 | −3.77 (0.82) | −5.38 to −2.16 | *<.001* | −0.60 |
|  | CR | 50% | 0.19 (0.46) | −0.71 to 1.10 | .68 | 0.04 | 1.96 (0.62) | 0.73 to 3.18 | *.002* | 0.33 | 0.14 (0.73) | −1.29 to 1.57 | | .85 | 0.02 | −2.78 (0.95) | −4.65 to −0.91 | *.004* | −0.40 |
|  | CR | 75% | 0.36 (0.50) | −0.62 to 1.35 | .47 | 0.07 | 3.19 (0.75) | 1.72 to 4.65 | *<.001* | 0.45 | 1.09 (0.84) | −0.55 to 2.74 | | .19 | 0.14 | −1.79 (1.09) | −3.93 to 0.36 | .10 | −0.23 |
|  | EXPO | 25% | 0.16 (0.38) | −0.58 to 0.90 | .66 | 0.04 | 0.22 (0.55) | −0.86 to 1.30 | .69 | 0.04 | −1.29 (0.57) | −2.40 to −0.18 | | .02 | −0.26 | −3.52 (0.71) | −4.91 to −2.13 | *<.001* | −0.58 |
|  | EXPO | 50% | 0.42 (0.42) | −0.40 to 1.23 | .32 | 0.10 | 1.40 (0.66) | 0.12 to 2.68 | .03 | 0.23 | −0.40 (0.65) | −1.68 to 0.88 | | .54 | −0.07 | −2.53 (0.81) | −4.13 to −0.94 | *.002* | −0.38 |
|  | EXPO | 75% | 0.67 (0.47) | −0.25 to 1.59 | .15 | 0.14 | 2.58 (0.78) | 1.05 to 4.11 | *.001* | 0.35 | 0.49 (0.76) | −0.99 to 1.98 | | .51 | 0.07 | −1.54 (0.93) | −3.37 to 0.29 | .10 | −0.20 |
|  | WAIT | 25% | −0.14 (0.41) | −0.95 to 0.67 | .73 | −0.03 | 0.19 (0.43) | −0.65 to 1.04 | .66 | 0.04 | 0.28 (0.52) | −0.75 to 1.30 | | .60 | 0.06 | −0.37 (0.44) | −1.23 to 0.50 | .41 | −0.08 |
|  | WAIT | 50% | 0.16 (0.46) | −0.75 to 1.06 | .73 | 0.03 | 0.94 (0.51) | −0.05 to 1.94 | .06 | 0.18 | 1.33 (0.61) | 0.13 to 2.53 | | .03 | 0.24 | 0.22 (0.51) | −0.77 to 1.21 | .67 | 0.04 |
|  | WAIT | 75% | 0.46 (0.52) | −0.57 to 1.48 | .38 | 0.08 | 1.70 (0.60) | 0.52 to 2.88 | .005 | 0.28 | 2.39 (0.72) | 0.97 to 3.80 | | *.001* | 0.36 | 0.80 (0.59) | −0.35 to 1.96 | .17 | 0.14 |

^a^All estimates are pooled across 100 imputed data sets. For sensitivity analysis, individuals scores imputed under MAR (missing at random) assumption were increased by 25%, 50% and 75%, respectively.

^b^PCL-5: Posttraumatic Stress Disorder Checklist for the Diagnostic and Statistical Manual of Mental Disorders, Fifth Edition.

^c^T2: assessment immediately before starting treatment or waiting time.

^d^T1: baseline assessment.

^e^T3: assessment after 2 letters or 1 week of waiting.

^f^T4: assessment after 4 letters or 2 weeks of waiting.

^g^T5: postassessment time point.

^h^M: change score (mean change in raw score units of the questionnaire).

^i^CR: cognitive restructuring treatment.

^j^Significant *P* values.

^k^EXPO: exposure treatment.

^l^WAIT: waitlist control group.

**Table S7.** Estimated between-group differences in posttraumatic stress symptom severity between baseline and subsequent assessments under various missing not at random conditions (25%, 50% and 75% increase of individual imputed values) in the intention-to-treat sample ^a^.

| Outcome (PCL-5^b^), group comparison and condition | | | T2^c^ − T1^d^ | | | | T3^e^ − T1 | | | | T4^f^ − T1 | | | | T5^g^ − T1 | | | |
| --- | --- | --- | --- | --- | --- | --- | --- | --- | --- | --- | --- | --- | --- | --- | --- | --- | --- | --- |
|  | | | ΔM^h^ (SE) | 95% CI | *P* value | *d* | ΔM (SE) | 95% CI | *P* value | *d* | ΔM (SE) | 95% CI | *P* value | *d* | ΔM (SE) | 95% CI | *P* value | *d* |
|  | | | | | | | | | | | | | | | | | | |
| **Overall** | | | | | | | | | | | | | | | | | | |
|  | CR^i^ vs. EXPO^j^ | 25% | −0.20 (1.53) | −3.19 to 2.80 | .90 | −0.02 | −1.49 (2.18) | −5.77 to 2.79 | .49 | −0.10 | −1.26 (2.39) | −5.95 to 3.43 | .60 | −0.08 | 2.24 (3.27) | −4.18 to 8.66 | .49 | 0.12 |
|  | CR vs. EXPO | 50% | 0.09 (1.69) | −3.21 to 3.39 | .96 | 0.01 | −1.69 (2.67) | −6.93 to 3.54 | .53 | −0.10 | −1.53 (2.78) | −6.99 to 3.92 | .58 | −0.08 | 2.36 (3.77) | −5.02 to 9.74 | .53 | 0.11 |
|  | CR vs. EXPO | 75% | 0.38 (1.90) | −3.36 to 4.11 | .84 | 0.03 | −1.90 (3.27) | −8.30 to 4.50 | .56 | −0.09 | −1.81 (3.27) | −8.22 to 4.60 | .58 | −0.08 | 2.48 (4.31) | −5.97 to 10.93 | .57 | 0.10 |
|  | EXPO vs. WAIT^k^ | 25% | −0.41 (1.60) | −3.55 to 2.73 | .80 | −0.03 | −0.97 (2.03) | −4.95 to 3.01 | .63 | −0.07 | 3.99 (2.15) | −0.24 to 8.21 | .06 | 0.28 | 11.97 (2.58) | 6.92 to 17.02 | *<.001^l^* | 0.75 |
|  | EXPO vs. WAIT | 50% | −0.26 (1.81) | −3.80 to 3.28 | .89 | −0.02 | −2.49 (2.44) | −7.27 to 2.30 | .31 | −0.14 | 4.49 (2.54) | −0.48 to 9.46 | .08 | 0.27 | 10.73 (2.99) | 4.88 to 16.58 | *<.001* | 0.59 |
|  | EXPO vs. WAIT | 75% | −0.10 (2.08) | −4.17 to 3.97 | .96 | −0.01 | −4.00 (2.95) | −9.79 to 1.79 | .18 | −0.19 | 5.00 (3.02) | −0.93 to 10.92 | .10 | 0.24 | 9.49 (3.46) | 2.70 to 16.28 | .006 | 0.45 |
|  | CR vs. WAIT | 25% | −0.61 (1.60) | −3.74 to 2.52 | .70 | −0.05 | −2.46 (1.80) | −6.00 to 1.07 | .17 | −0.19 | 2.73 (2.25) | −1.67 to 7.13 | .22 | 0.18 | 14.21 (2.82) | 8.68 to 19.73 | *<.001* | 0.84 |
|  | CR vs. WAIT | 50% | −0.17 (1.81) | −3.72 to 3.39 | .93 | −0.01 | −4.18 (2.23) | −8.55 to 0.19 | .06 | −0.26 | 2.96 (2.65) | −2.24 to 8.16 | .27 | 0.17 | 13.09 (3.26) | 6.70 to 19.47 | *<.001* | 0.68 |
|  | CR vs. WAIT | 75% | 0.28 (2.08) | −3.80 to 4.35 | .89 | 0.02 | −5.90 (2.77) | −11.32 to −0.48 | .03 | −0.30 | 3.19 (3.17) | −3.02 to 9.40 | .32 | 0.15 | 11.97 (3.76) | 4.60 to 19.34 | *.001* | 0.55 |
| **Re-experiencing** | | | | | | | | | | | | | | | | | | |
|  | CR vs. EXPO | 25% | 0.49 (0.49) | −0.47 to 1.46 | .32 | 0.14 | 0.77 (0.71) | −0.62 to 2.16 | .28 | 0.17 | 0.34 (0.73) | −1.10 to 1.78 | .64 | 0.07 | 1.53 (0.96) | −0.36 to 3.42 | .11 | 0.26 |
|  | CR vs. EXPO | 50% | 0.58 (0.53) | −0.45 to 1.60 | .27 | 0.15 | 0.84 (0.82) | −0.77 to 2.46 | .31 | 0.17 | 0.34 (0.84) | −1.30 to 1.98 | .68 | 0.07 | 1.70 (1.09) | −0.44 to 3.84 | .12 | 0.26 |
|  | CR vs. EXPO | 75% | 0.66 (0.57) | −0.45 to 1.77 | .25 | 0.16 | 0.92 (0.96) | −0.97 to 2.81 | .34 | 0.15 | 0.34 (0.96) | −1.54 to 2.22 | .72 | 0.06 | 1.86 (1.23) | −0.55 to 4.28 | .13 | 0.26 |
|  | EXPO vs. WAIT | 25% | −0.67 (0.54) | −1.72 to 0.39 | .22 | −0.17 | −1.38 (0.66) | −2.68 to −0.07 | .04 | −0.30 | 0.11 (0.72) | −1.29 to 1.52 | .88 | 0.02 | 1.92 (0.86) | 0.23 to 3.61 | .03 | 0.36 |
|  | EXPO vs. WAIT | 50% | −0.63 (0.58) | −1.77 to 0.51 | .28 | −0.15 | −1.79 (0.76) | −3.27 to −0.30 | .02 | −0.35 | 0.20 (0.82) | −1.42 to 1.81 | .81 | 0.04 | 1.67 (0.98) | −0.26 to 3.60 | .09 | 0.28 |
|  | EXPO vs. WAIT | 75% | −0.59 (0.64) | −1.86 to 0.67 | .36 | −0.13 | −2.20 (0.87) | −3.91 to −0.48 | .01 | −0.38 | 0.28 (0.95) | −1.58 to 2.14 | .77 | 0.05 | 1.41 (1.12) | −0.79 to 3.62 | .21 | 0.21 |
|  | CR vs. WAIT | 25% | −0.17 (0.56) | −1.27 to 0.93 | .76 | −0.04 | −0.61 (0.67) | −1.92 to 0.70 | .37 | −0.14 | 0.46 (0.74) | −0.99 to 1.90 | .54 | 0.10 | 3.45 (0.86) | 1.76 to 5.15 | *<.001* | 0.64 |
|  | CR vs. WAIT | 50% | −0.05 (0.60) | −1.23 to 1.13 | .93 | −0.01 | −0.94 (0.77) | −2.44 to 0.56 | .22 | −0.19 | 0.54 (0.85) | −1.13 to 2.21 | .53 | 0.10 | 3.36 (0.98) | 1.45 to 5.28 | *.001* | 0.57 |
|  | CR vs. WAIT | 75% | 0.06 (0.66) | −1.22 to 1.34 | .93 | 0.01 | −1.28 (0.89) | −3.01 to 0.46 | .15 | −0.23 | 0.62 (0.98) | −1.30 to 2.54 | .53 | 0.10 | 3.27 (1.10) | 1.11 to 5.44 | *.003* | 0.50 |
| **Avoidance** | | | | | | | | | | | | | | | | | | |
|  | CR vs. EXPO | 25% | 0.01 (0.31) | −0.60 to 0.62 | .99 | 0.00 | −0.35 (0.43) | −1.19 to 0.49 | .41 | −0.13 | −0.58 (0.41) | −1.39 to 0.23 | .16 | −0.22 | −0.21 (0.55) | −1.29 to 0.88 | .71 | −0.07 |
|  | CR vs. EXPO | 50% | 0.04 (0.33) | −0.60 to 0.68 | .90 | 0.02 | −0.39 (0.49) | −1.35 to 0.58 | .43 | −0.12 | −0.66 (0.47) | −1.57 to 0.25 | .16 | −0.22 | −0.24 (0.63) | −1.48 to 1.00 | .71 | −0.07 |
|  | CR vs. EXPO | 75% | 0.08 (0.35) | −0.60 to 0.76 | .82 | 0.03 | −0.42 (0.57) | −1.53 to 0.69 | .46 | −0.12 | −0.74 (0.53) | −1.77 to 0.30 | .16 | −0.22 | −0.26 (0.72) | −1.67 to 1.14 | .71 | −0.07 |
|  | EXPO vs. WAIT | 25% | 0.31 (0.31) | −0.29 to 0.92 | .31 | 0.13 | 0.10 (0.39) | −0.67 to 0.87 | .79 | 0.04 | 0.65 (0.40) | −0.14 to 1.44 | .11 | 0.25 | 1.96 (0.46) | 1.07 to 2.85 | *<.001* | 0.68 |
|  | EXPO vs. WAIT | 50% | 0.33 (0.33) | −0.32 to 0.97 | .32 | 0.13 | −0.08 (0.45) | −0.96 to 0.80 | .86 | −0.03 | 0.71 (0.46) | −0.19 to 1.61 | .12 | 0.24 | 1.82 (0.52) | 0.81 to 2.84 | *<.001* | 0.58 |
|  | EXPO vs. WAIT | 75% | 0.34 (0.36) | −0.36 to 1.04 | .34 | 0.13 | −0.26 (0.51) | −1.27 to 0.75 | .61 | −0.07 | 0.77 (0.53) | −0.26 to 1.79 | .15 | 0.23 | 1.69 (0.58) | 0.54 to 2.83 | *.004* | 0.48 |
|  | CR vs. WAIT | 25% | 0.32 (0.32) | −0.32 to 0.95 | .33 | 0.13 | −0.25 (0.38) | −0.99 to 0.49 | .51 | −0.09 | 0.07 (0.37) | −0.65 to 0.80 | .84 | 0.03 | 1.75 (0.46) | 0.86 to 2.65 | *<.001* | 0.63 |
|  | CR vs. WAIT | 50% | 0.37 (0.34) | −0.30 to 1.04 | .28 | 0.14 | −0.46 (0.43) | −1.31 to 0.38 | .28 | −0.15 | 0.05 (0.42) | −0.78 to 0.88 | .91 | 0.02 | 1.59 (0.52) | 0.57 to 2.61 | *.002* | 0.52 |
|  | CR vs. WAIT | 75% | 0.42 (0.37) | −0.30 to 1.14 | .25 | 0.15 | −0.68 (0.49) | −1.64 to 0.29 | .17 | −0.20 | 0.03 (0.49) | −0.93 to 0.99 | .96 | 0.01 | 1.42 (0.59) | 0.27 to 2.58 | .02 | 0.42 |
| **Negative alterations in cognitions and mood** | | | | | | | | | | | | | | | | | | |
|  | CR vs. EXPO | 25% | −0.84 (0.75) | −2.31 to 0.63 | .26 | −0.15 | −1.41 (0.96) | −3.29 to 0.48 | .15 | −0.22 | −0.54 (1.09) | −2.69 to 1.60 | .62 | −0.07 | 0.67 (1.48) | −2.22 to 3.56 | .65 | 0.08 |
|  | CR vs. EXPO | 50% | −0.75 (0.80) | −2.33 to 0.82 | .35 | −0.13 | −1.60 (1.15) | −3.86 to 0.66 | .17 | −0.21 | −0.68 (1.25) | −3.14 to 1.78 | .59 | −0.08 | 0.65 (1.70) | −2.68 to 3.99 | .70 | 0.07 |
|  | CR vs. EXPO | 75% | −0.67 (0.88) | −2.40 to 1.06 | .45 | −0.10 | −1.79 (1.38) | −4.50 to 0.91 | .19 | −0.19 | −0.81 (1.45) | −3.65 to 2.02 | .57 | −0.09 | 0.64 (1.95) | −3.18 to 4.46 | .74 | 0.06 |
|  | EXPO vs. WAIT | 25% | 0.24 (0.73) | −1.20 to 1.68 | .74 | 0.04 | 0.33 (0.91) | −1.45 to 2.10 | .72 | 0.05 | 1.66 (0.94) | −0.18 to 3.49 | .08 | 0.26 | 4.93 (1.13) | 2.72 to 7.15 | *<.001* | 0.72 |
|  | EXPO vs. WAIT | 50% | 0.31 (0.80) | −1.26 to 1.88 | .70 | 0.05 | −0.17 (1.06) | −2.25 to 1.91 | .88 | −0.02 | 1.86 (1.08) | −0.26 to 3.97 | .09 | 0.26 | 4.49 (1.30) | 1.93 to 7.05 | *.001* | 0.59 |
|  | EXPO vs. WAIT | 75% | 0.37 (0.89) | −1.38 to 2.12 | .68 | 0.06 | −0.66 (1.25) | −3.12 to 1.80 | .60 | −0.08 | 2.06 (1.26) | −0.41 to 4.53 | .10 | 0.24 | 4.04 (1.50) | 1.10 to 6.99 | .007 | 0.46 |
|  | CR vs. WAIT | 25% | −0.59 (0.69) | −1.95 to 0.76 | .39 | −0.12 | −1.08 (0.79) | −2.63 to 0.47 | .17 | −0.19 | 1.11 (0.96) | −0.78 to 3.00 | .25 | 0.17 | 5.60 (1.23) | 3.19 to 8.01 | *<.001* | 0.76 |
|  | CR vs. WAIT | 50% | −0.45 (0.77) | −1.96 to 1.07 | .56 | −0.08 | −1.76 (0.96) | −3.65 to 0.12 | .07 | −0.26 | 1.18 (1.12) | −1.03 to 3.38 | .30 | 0.16 | 5.14 (1.42) | 2.35 to 7.93 | *<.001* | 0.62 |
|  | CR vs. WAIT | 75% | −0.30 (0.87) | −2.01 to 1.41 | .73 | −0.05 | −2.45 (1.17) | −4.75 to −0.16 | .04 | −0.30 | 1.25 (1.32) | −1.35 to 3.84 | .35 | 0.14 | 4.68 (1.63) | 1.48 to 7.88 | *.004* | 0.50 |
| **Hyperarousal** | | | | | | | | | | | | | | | | | | |
|  | CR vs. EXPO | 25% | 0.14 (0.57) | −0.98 to 1.26 | .80 | 0.03 | −0.50 (0.78) | −2.04 to 1.03 | .52 | −0.10 | −0.48 (0.86) | −2.16 to 1.21 | .58 | −0.09 | 0.25 (1.11) | −1.93 to 2.42 | .82 | 0.04 |
|  | CR vs. EXPO | 50% | 0.23 (0.62) | −0.99 to 1.44 | .72 | 0.05 | −0.56 (0.94) | −2.39 to 1.28 | .55 | −0.09 | −0.54 (0.99) | −2.48 to 1.40 | .59 | −0.09 | 0.24 (1.29) | −2.28 to 2.77 | .85 | 0.04 |
|  | CR vs. EXPO | 75% | 0.31 (0.69) | −1.04 to 1.65 | .65 | 0.06 | −0.61 (1.12) | −2.80 to 1.58 | .59 | −0.09 | −0.60 (1.15) | −2.84 to 1.64 | .60 | −0.09 | 0.24 (1.48) | −2.65 to 3.14 | .87 | 0.03 |
|  | EXPO vs. WAIT | 25% | −0.30 (0.56) | −1.40 to 0.79 | .59 | −0.07 | −0.03 (0.70) | −1.39 to 1.34 | .97 | −0.01 | 1.56 (0.77) | 0.06 to 3.07 | .04 | 0.32 | 3.16 (0.84) | 1.51 to 4.80 | *<.001* | 0.59 |
|  | EXPO vs. WAIT | 50% | −0.26 (0.62) | −1.48 to 0.96 | .68 | −0.06 | −0.46 (0.82) | −2.07 to 1.16 | .58 | −0.08 | 1.73 (0.89) | −0.02 to 3.48 | .05 | 0.31 | 2.75 (0.96) | 0.86 to 4.64 | *.004* | 0.46 |
|  | EXPO vs. WAIT | 75% | −0.21 (0.70) | −1.59 to 1.16 | .76 | −0.04 | −0.88 (0.98) | −2.81 to 1.04 | .37 | −0.13 | 1.89 (1.04) | −0.15 to 3.94 | .07 | 0.29 | 2.35 (1.11) | 0.17 to 4.52 | .04 | 0.34 |
|  | CR vs. WAIT | 25% | −0.16 (0.60) | −1.33 to 1.01 | .79 | −0.04 | −0.53 (0.68) | −1.86 to 0.80 | .43 | −0.11 | 1.09 (0.83) | −0.55 to 2.72 | .19 | 0.20 | 3.40 (0.92) | 1.59 to 5.21 | *<.001* | 0.63 |
|  | CR vs. WAIT | 50% | −0.04 (0.66) | −1.32 to 1.25 | .96 | −0.01 | −1.01 (0.80) | −2.58 to 0.56 | .21 | −0.18 | 1.19 (0.96) | −0.70 to 3.08 | .22 | 0.20 | 3.00 (1.07) | 0.90 to 5.09 | *.004* | 0.49 |
|  | CR vs. WAIT | 75% | 0.09 (0.73) | −1.33 to 1.52 | .90 | 0.02 | −1.49 (0.96) | −3.37 to 0.39 | .12 | −0.22 | 1.29 (1.12) | −0.91 to 3.50 | .25 | 0.18 | 2.59 (1.23) | 0.18 to 5.00 | .04 | 0.38 |

^a^All estimates are pooled across 100 imputed data sets. For sensitivity analysis, individuals scores imputed under MAR (missing at random) assumption were increased by 25%, 50% and 75%, respectively.

^b^PCL-5: Posttraumatic Stress Disorder Checklist for the Diagnostic and Statistical Manual of Mental Disorders, Fifth Edition.

^c^T2: assessment immediately before starting treatment or waiting time.

^d^T1: baseline assessment.

^e^T3: assessment after 2 letters or 1 week of waiting.

^f^T4: assessment after 4 letters or 2 weeks of waiting.

^g^T5: postassessment time point.

^h^ΔM: difference between group-specific means of change scores.

^i^CR: cognitive restructuring treatment.

^j^EXPO: exposure treatment.

^k^WAIT: waitlist control group.

^l^Significant *P* values.

**Table S8.** Rates of reliable change, remission, and reliable change and significant improvement (RCSI) under various missing not at random conditions (25%, 50% and 75% increase of individual imputed values) in the intention-to-treat-sample^a^.

|  | Baseline assessment to postassessment time point (PCL-5^b^), n (%) | | |
| --- | --- | --- | --- |
|  | CR^c^ (n=118) | EXPO^d^ (n=122) | WAIT^e^ (n=125) |
|  | | | |
| **MAR^f^ imputation + 25%** | | | |
| Reliable change | | | |
| Improvement | 58.54 (49.6) | 60.06 (49.2) | 19.56 (15.6) |
| No change | 52.13 (44.2) | 53.69 (44) | 91.56 (73.2) |
| Deterioration | 7.33 (6.2) | 8.25 (6.8) | 13.88 (11.1) |
| Remission | 43.59 (36.9) | 39.78 (32.6) | 4.2 (3.4) |
| RCSI | 38.72 (32.8) | 36.33 (29.8) | 3.08 (2.5) |
| **MAR imputation + 50%** | | | |
| Reliable change | | | |
| Improvement | 52.96 (44.9) | 53.89 (44.2) | 18.43 (14.7) |
| No change | 52.65 (44.6) | 54.34 (44.5) | 86.49 (69.2) |
| Deterioration | 12.39 (10.5) | 13.77 (11.3) | 20.08 (16.1) |
| Remission | 40 (33.9) | 35.59 (29.2) | 4.06 (3.2) |
| RCSI | 35.19 (29.8) | 32.52 (26.7) | 3.01 (2.4) |
| **MAR imputation + 75%** | | | |
| Reliable change | | | |
| Improvement | 48.9 (41.4) | 49.56 (40.6) | 18.18 (14.5) |
| No change | 51.67 (43.8) | 52.7 (43.2) | 81.78 (65.4) |
| Deterioration | 17.43 (14.8) | 19.74 (16.2) | 25.04 (20) |
| Remission | 37.78 (32) | 33.22 (27.2) | 4.03 (3.2) |
| RCSI | 33.02 (28) | 30.36 (24.9) | 3 (2.4) |

^a^All results are averaged across imputed data sets; counts therefore contain decimals.

^b^PCL-5: Posttraumatic Stress Disorder Checklist for the Diagnostic and Statistical Manual of Mental Disorders, Fifth Edition. An increase or decrease of 16 PCL-5 points between the baseline and postassessment time points was defined as reliable deterioration or improvement, respectively. Remission was defined as a score of ≥23 at baseline and a score of <23 at the postassessment time point. RCSI was defined as experiencing both remission and reliable improvement from baseline to the postassessment time point.

^c^CR: cognitive restructuring treatment.

^d^EXPO: exposure treatment.

^e^WAIT: waitlist control group.

^f^MAR: missing at random.
