# Supplementary material for: Exposure Versus Cognitive Restructuring Techniques in Brief Internet-Based Cognitive Behavioral Treatment for Arabic-Speaking People With Posttraumatic Stress Disorder: Randomized Clinical Trial
Source: JMIR Ment Health. 2023 Dec 13;10:e48689. doi: 10.2196/48689 (PMC10753431; doi:10.2196/48689)
Supplement: Multimedia Appendix 2 [file mental_v10i1e48689_app2.pdf]

CONSORT-EHEALTH (V 1.6.1) - Submission/Publication Form

The CONSORT-EHEALTH checklist is intended for authors of randomized trials evaluating web-based and Internet-based applications/interventions, including mobile interventions, electronic games (incl multiplayer games), social media, certain telehealth applications, and other interactive and/or networked electronic applications. Some of the items (e.g. all subitems under item 5 - description of the intervention) may also be applicable for other study designs.

The goal of the CONSORT EHEALTH checklist and guideline is to be
a) a guide for reporting for authors of RCTs,
b) to form a basis for appraisal of an ehealth trial (in terms of validity)

CONSORT-EHEALTH items/subitems are MANDATORY reporting items for studies published in the Journal of Medical Internet Research and other journals / scientific societies endorsing the checklist.

Items numbered 1., 2., 3., 4a., 4b etc are original CONSORT or CONSORT-NPT (non-pharmacologic treatment) items.
Items with Roman numerals (i., ii, iii, iv etc.) are CONSORT-EHEALTH extensions/clarifications.

As the CONSORT-EHEALTH checklist is still considered in a formative stage, we would ask that you also RATE ON A SCALE OF 1-5 how important/useful you feel each item is FOR THE PURPOSE OF THE CHECKLIST and reporting guideline (optional).

Mandatory reporting items are marked with a red *.
In the textboxes, either copy & paste the relevant sections from your manuscript into this form - please include any quotes from your manuscript in QUOTATION MARKS,
or answer directly by providing additional information not in the manuscript, or elaborating on why the item was not relevant for this study.

YOUR ANSWERS WILL BE PUBLISHED AS A SUPPLEMENTARY FILE TO YOUR PUBLICATION IN JMIR AND ARE CONSIDERED PART OF YOUR PUBLICATION (IF ACCEPTED).
Please fill in these questions diligently. Information will not be copyedited, so please use proper spelling and grammar, use correct capitalization, and avoid abbreviations.

DO NOT FORGET TO SAVE AS PDF _AND_ CLICK THE SUBMIT BUTTON SO YOUR ANSWERS ARE IN OUR DATABASE !!!

Citation Suggestion (if you append the pdf as Appendix we suggest to cite this paper in the caption):

Eysenbach G, CONSORT-EHEALTH Group
CONSORT-EHEALTH: Improving and Standardizing Evaluation Reports of Web-based and Mobile Health Interventions
J Med Internet Res 2011;13(4):e126
URL: http://www.jmir.org/2011/4/e126/
doi: 10.2196/jmir.1923
PMID: 22209829

In Google anmelden, um den Fortschritt zu speichern. Weitere Informationen

* Gibt eine erforderliche Frage an

Your name *
First Last

Jana Stein

Primary Affiliation (short), City, Country *
University of Toronto, Toronto, Canada

Freie Universität Berlin, Germany

Your e-mail address *
abc@gmail.com

j.stein@ueberleben.org

Title of your manuscript *

Provide the (draft) title of your manuscript.

Exposure versus cognitive restructuring techniques in brief internet-based cognitive behavioral treatment for Arabic-speaking people with PTSD: A randomized clinical trial

Name of your App/Software/Intervention *

If there is a short and a long/alternate name, write the short name first and add the long name in brackets.

Ilajnafsy

Evaluated Version (if any)

e.g. "V1", "Release 2017-03-01", "Version 2.0.27913"

Meine Antwort

Language(s) *

What language is the intervention/app in? If multiple languages are available, separate by comma (e.g. "English, French")

Arabic

URL of your Intervention Website or App

e.g. a direct link to the mobile app on app in appstore (itunes, Google Play), or URL of the website. If the intervention is a DVD or hardware, you can also link to an Amazon page.

https://ilajnafsy.bzfo.de/portal/

URL of an image/screenshot (optional)

Meine Antwort

Accessibility *

Can an enduser access the intervention presently?

- (x) access is free and open
- ( ) access only for special usergroups, not open
- ( ) access is open to everyone, but requires payment/subscription/in-app purchases
- ( ) app/intervention no longer accessible
- ( ) Sonstiges:

Primary Medical Indication/Disease/Condition *

e.g. "Stress", "Diabetes", or define the target group in brackets after the condition, e.g. "Autism (Parents of children with)", "Alzheimers (Informal Caregivers of)"

Posttraumatic stress disorder

Primary Outcomes measured in trial *

comma-separated list of primary outcomes reported in the trial

Posttraumatic stress symptom severity

Secondary/other outcomes

Are there any other outcomes the intervention is expected to affect?

posttraumatic maladaptive cognitions, anxiety symptom severity, depressive symptom severity and somatoform symptom severity, and quality of life

Recommended "Dose" *

What do the instructions for users say on how often the app should be used?

- ○ Approximately Daily
- ◉ Approximately Weekly
- ○ Approximately Monthly
- ○ Approximately Yearly
- ○ "as needed"
- ○ Sonstiges:

Approx. Percentage of Users (starters) still using the app as recommended after 3 months *

- (x) unknown / not evaluated
- ( ) 0-10%
- ( ) 11-20%
- ( ) 21-30%
- ( ) 31-40%
- ( ) 41-50%
- ( ) 51-60%
- ( ) 61-70%
- ( ) 71%-80%
- ( ) 81-90%
- ( ) 91-100%
- ( ) Sonstiges:

Overall, was the app/intervention effective? *

- (x) yes: all primary outcomes were significantly better in intervention group vs control
- ( ) partly: SOME primary outcomes were significantly better in intervention group vs control
- ( ) no statistically significant difference between control and intervention
- ( ) potentially harmful: control was significantly better than intervention in one or more outcomes
- ( ) inconclusive: more research is needed
- ( ) Sonstiges:

Article Preparation Status/Stage *

At which stage in your article preparation are you currently (at the time you fill in this form)

- ( ) not submitted yet - in early draft status
- ( ) not submitted yet - in late draft status, just before submission
- ( ) submitted to a journal but not reviewed yet
- ( ) submitted to a journal and after receiving initial reviewer comments
- (x) submitted to a journal and accepted, but not published yet
- ( ) published
- ( ) Sonstiges:

Journal *

If you already know where you will submit this paper (or if it is already submitted), please provide the journal name (if it is not JMIR, provide the journal name under "other")

- ◯ not submitted yet / unclear where I will submit this
- ◯ Journal of Medical Internet Research (JMIR)
- ◯ JMIR mHealth and UHealth
- ◯ JMIR Serious Games
- ◉ JMIR Mental Health
- ◯ JMIR Public Health
- ◯ JMIR Formative Research
- ◯ Other JMIR sister journal
- ◯ Sonstiges:

Is this a full powered effectiveness trial or a pilot/feasibility trial? *

- ◯ Pilot/feasibility
- ◉ Fully powered

Manuscript tracking number *

If this is a JMIR submission, please provide the manuscript tracking number under "other" (The ms tracking number can be found in the submission acknowledgement email, or when you login as author in JMIR. If the paper is already published in JMIR, then the ms tracking number is the four-digit number at the end of the DOI, to be found at the bottom of each published article in JMIR)

- ○ no ms number (yet) / not (yet) submitted to / published in JMIR
- ◉ Sonstiges: 48689

TITLE AND ABSTRACT

1a) TITLE: Identification as a randomized trial in the title

1a) Does your paper address CONSORT item 1a? *

I.e does the title contain the phrase "Randomized Controlled Trial"? (if not, explain the reason under "other")

- ○ yes
- ◉ Sonstiges: Randomized Clinical Trial

1a-i) Identify the mode of delivery in the title

Identify the mode of delivery. Preferably use “web-based” and/or “mobile” and/or “electronic game” in the title. Avoid ambiguous terms like “online”, “virtual”, “interactive”. Use “Internet-based” only if Intervention includes non-web-based Internet components (e.g. email), use “computer-based” or “electronic” only if offline products are used. Use “virtual” only in the context of “virtual reality” (3-D worlds). Use “online” only in the context of “online support groups”. Complement or substitute product names with broader terms for the class of products (such as “mobile” or “smart phone” instead of “iphone”), especially if the application runs on different platforms.

	1	2	3	4	5	
subitem not at all important	○	○	○	◉	○	essential

Auswahl löschen

Does your paper address subitem 1a-i? *

Copy and paste relevant sections from manuscript title (include quotes in quotation marks "like this" to indicate direct quotes from your manuscript), or elaborate on this item by providing additional information not in the ms, or briefly explain why the item is not applicable/relevant for your study

Exposure versus cognitive restructuring techniques in brief internet-based cognitive behavioral treatment for Arabic-speaking people with PTSD: A randomized clinical trial

1a-ii) Non-web-based components or important co-interventions in title

Mention non-web-based components or important co-interventions in title, if any (e.g., “with telephone support”).

	1	2	3	4	5	
subitem not at all important	○	◉	○	○	○	essential

Auswahl löschen

Does your paper address subitem 1a-ii?

Copy and paste relevant sections from manuscript title (include quotes in quotation marks "like this" to indicate direct quotes from your manuscript), or elaborate on this item by providing additional information not in the ms, or briefly explain why the item is not applicable/relevant for your study

Meine Antwort

1a-iii) Primary condition or target group in the title

Mention primary condition or target group in the title, if any (e.g., "for children with Type I Diabetes") Example: A Web-based and Mobile Intervention with Telephone Support for Children with Type I Diabetes: Randomized Controlled Trial

	1	2	3	4	5	
subitem not at all important	○	○	○	◉	○	essential

Auswahl löschen

Does your paper address subitem 1a-iii? *

Copy and paste relevant sections from manuscript title (include quotes in quotation marks "like this" to indicate direct quotes from your manuscript), or elaborate on this item by providing additional information not in the ms, or briefly explain why the item is not applicable/relevant for your study

Exposure versus cognitive restructuring techniques in brief internet-based cognitive behavioral treatment for Arabic-speaking people with PTSD: A randomized clinical trial

1b) ABSTRACT: Structured summary of trial design, methods, results, and conclusions

NPT extension: Description of experimental treatment, comparator, care providers, centers, and blinding status.

1b-i) Key features/functionalities/components of the intervention and comparator in the METHODS section of the ABSTRACT

Mention key features/functionalities/components of the intervention and comparator in the abstract. If possible, also mention theories and principles used for designing the site. Keep in mind the needs of systematic reviewers and indexers by including important synonyms. (Note: Only report in the abstract what the main paper is reporting. If this information is missing from the main body of text, consider adding it)

	1	2	3	4	5	
subitem not at all important	○	○	○	○	◉	essential

Auswahl löschen

Does your paper address subitem 1b-i? *

Copy and paste relevant sections from the manuscript abstract (include quotes in quotation marks "like this" to indicate direct quotes from your manuscript), or elaborate on this item by providing additional information not in the ms, or briefly explain why the item is not applicable/relevant for your study

Both treatments were developed in line with Interapy, an internet-based, therapist-assisted cognitive behavioral therapy protocol for PTSD and adapted to the specific research question. The first treatment comprised self-confrontation and social sharing (EXPO; 6 sessions) and the second comprised cognitive restructuring and social sharing (CR; 6 sessions). The two treatments were compared with each other and with a waitlist control group (WAIT).

1b-ii) Level of human involvement in the METHODS section of the ABSTRACT

Clarify the level of human involvement in the abstract, e.g., use phrases like “fully automated” vs. “therapist/nurse/care provider/physician-assisted” (mention number and expertise of providers involved, if any). (Note: Only report in the abstract what the main paper is reporting. If this information is missing from the main body of text, consider adding it)

	1	2	3	4	5	
subitem not at all important	○	○	◉	○	○	essential

Auswahl löschen

Does your paper address subitem 1b-ii?

Copy and paste relevant sections from the manuscript abstract (include quotes in quotation marks "like this" to indicate direct quotes from your manuscript), or elaborate on this item by providing additional information not in the ms, or briefly explain why the item is not applicable/relevant for your study

Meine Antwort

1b-iii) Open vs. closed, web-based (self-assessment) vs. face-to-face assessments in the METHODS section of the ABSTRACT

Mention how participants were recruited (online vs. offline), e.g., from an open access website or from a clinic or a closed online user group (closed usergroup trial), and clarify if this was a purely web-based trial, or there were face-to-face components (as part of the intervention or for assessment). Clearly say if outcomes were self-assessed through questionnaires (as common in web-based trials). Note: In traditional offline trials, an open trial (open-label trial) is a type of clinical trial in which both the researchers and participants know which treatment is being administered. To avoid confusion, use “blinded” or “unblinded” to indicated the level of blinding instead of “open”, as “open” in web-based trials usually refers to “open access” (i.e. participants can self-enrol). (Note: Only report in the abstract what the main paper is reporting. If this information is missing from the main body of text, consider adding it)

	1	2	3	4	5	
subitem not at all important	○	○	◉	○	○	essential

Auswahl löschen

Does your paper address subitem 1b-iii?

Copy and paste relevant sections from the manuscript abstract (include quotes in quotation marks "like this" to indicate direct quotes from your manuscript), or elaborate on this item by providing additional information not in the ms, or briefly explain why the item is not applicable/relevant for your study

Meine Antwort

1b-iv) RESULTS section in abstract must contain use data

Report number of participants enrolled/assessed in each group, the use/uptake of the intervention (e.g., attrition/adherence metrics, use over time, number of logins etc.), in addition to primary/secondary outcomes. (Note: Only report in the abstract what the main paper is reporting. If this information is missing from the main body of text, consider adding it)

	1	2	3	4	5	
subitem not at all important	○	○	○	●	○	essential

Auswahl löschen

Does your paper address subitem 1b-iv?

Copy and paste relevant sections from the manuscript abstract (include quotes in quotation marks "like this" to indicate direct quotes from your manuscript), or elaborate on this item by providing additional information not in the ms, or briefly explain why the item is not applicable/relevant for your study

Meine Antwort

1b-v) CONCLUSIONS/DISCUSSION in abstract for negative trials

Conclusions/Discussions in abstract for negative trials: Discuss the primary outcome - if the trial is negative (primary outcome not changed), and the intervention was not used, discuss whether negative results are attributable to lack of uptake and discuss reasons. (Note: Only report in the abstract what the main paper is reporting. If this information is missing from the main body of text, consider adding it)

	1	2	3	4	5	
subitem not at all important	○	○	○	●	○	essential

Auswahl löschen

Does your paper address subitem 1b-v?

Copy and paste relevant sections from the manuscript abstract (include quotes in quotation marks "like this" to indicate direct quotes from your manuscript), or elaborate on this item by providing additional information not in the ms, or briefly explain why the item is not applicable/relevant for your study

Meine Antwort

INTRODUCTION

2a) In INTRODUCTION: Scientific background and explanation of rationale

2a-i) Problem and the type of system/solution

Describe the problem and the type of system/solution that is object of the study: intended as stand-alone intervention vs. incorporated in broader health care program? Intended for a particular patient population? Goals of the intervention, e.g., being more cost-effective to other interventions, replace or complement other solutions? (Note: Details about the intervention are provided in "Methods" under 5)

	1	2	3	4	5	
subitem not at all important	○	○	○	●	○	essential

Auswahl löschen

Does your paper address subitem 2a-i? *

Copy and paste relevant sections from the manuscript (include quotes in quotation marks "like this" to indicate direct quotes from your manuscript), or elaborate on this item by providing additional information not in the ms, or briefly explain why the item is not applicable/relevant for your study

"While exposure-based techniques delivered online have been successfully implemented for Arabic-speaking people with PTSD [overall PTSD symptom improvement during treatment: d = 1.13], exposure might not be a suitable treatment option for all individuals suffering from PTSD, as some may be unwilling to confront themselves with the traumatic event in detail and may drop out of the intervention before treatment gains become apparent. In particular, for people in Arabic-speaking cultures who have experienced any form of sexual violence, going through the traumatic event in detail may be a huge burden, as this type of trauma is likely to be associated with great shame, loss of honor, and/or feelings of guilt. For internet-delivered interventions combining exposure and cognitive methods in Arabic-speaking people with PTSD, dropout rates of approximately 37% have been reported. Similar dropout rates were found when only providing exposure treatment for this population, highlighting the need for additional treatment options without a focus on exposure."

2a-ii) Scientific background, rationale: What is known about the (type of) system

Scientific background, rationale: What is known about the (type of) system that is the object of the study (be sure to discuss the use of similar systems for other conditions/diagnoses, if appropiate), motivation for the study, i.e. what are the reasons for and what is the context for this specific study, from which stakeholder viewpoint is the study performed, potential impact of findings [2]. Briefly justify the choice of the comparator.

	1	2	3	4	5	
subitem not at all important	○	○	○	◉	○	essential

Auswahl löschen

Does your paper address subitem 2a-ii? *

Copy and paste relevant sections from the manuscript (include quotes in quotation marks "like this" to indicate direct quotes from your manuscript), or elaborate on this item by providing additional information not in the ms, or briefly explain why the item is not applicable/relevant for your study

"Studies conducted in face-to-face psychotherapy settings have proven that both exposure and cognitive methods have beneficial effects on PTSD and comorbid mental health symptoms. However, little research has investigated differential effects of cognitive and exposure-based treatments by comparing the two treatment techniques with each other directly and with a passive control group in internet-based settings. To the best of our knowledge, no study has addressed this topic in Arabic-speaking populations. Therefore, addressing this issue is of considerable practical relevance, especially in areas with limited access to treatment."

2b) In INTRODUCTION: Specific objectives or hypotheses

Does your paper address CONSORT subitem 2b? *

Copy and paste relevant sections from the manuscript (include quotes in quotation marks "like this" to indicate direct quotes from your manuscript), or elaborate on this item by providing additional information not in the ms, or briefly explain why the item is not applicable/relevant for your study

"The aim of the study was to evaluate two brief internet-based treatments - one including cognitive restructuring as the main treatment component and the other including exposure treatment - for Arabic-speaking participants with PTSD. Specifically, we sought to examine the association of the two treatment conditions with treatment use, investigating the proportion of individuals who started treatment and the proportion who dropped out during treatment. We expected that the proportion of treatment starters and of treatment dropouts would not differ between the two treatment conditions. Furthermore, we compared completers' treatment satisfaction between the two treatment conditions, and again did not expect any differences between conditions. Finally, changes in posttraumatic stress symptom severity, posttraumatic cognitions, anxiety, depressive, and somatoform symptom severity, and quality of life during the two treatments were examined and compared to a waitlist control group. Based on previous research, we assumed that both treatments would lead to significant improvements in all treatment outcomes between baseline and post-treatment. We expected that the two treatments would lead to similar changes in terms of treatment outcomes and would outperform the waitlist control group."

METHODS

3a) Description of trial design (such as parallel, factorial) including allocation ratio

Does your paper address CONSORT subitem 3a? *

Copy and paste relevant sections from the manuscript (include quotes in quotation marks "like this" to indicate direct quotes from your manuscript), or elaborate on this item by providing additional information not in the ms, or briefly explain why the item is not applicable/relevant for your study

"The allocation schedule was created with the R package Blockrand and embedded in the web portal. Allocation to one of the three conditions was performed invisibly and automatically on the web portal itself and was thus concealed, i.e. participants, counselors, and researchers had no prior knowledge of, and therefore no control over, the group to which a participant would be allocated. Due to the nature of the provided treatments, participants and counselors could not be blinded regarding the treatment condition received. "

3b) Important changes to methods after trial commencement (such as eligibility criteria), with reasons

Does your paper address CONSORT subitem 3b? *

Copy and paste relevant sections from the manuscript (include quotes in quotation marks "like this" to indicate direct quotes from your manuscript), or elaborate on this item by providing additional information not in the ms, or briefly explain why the item is not applicable/relevant for your study

No changes after trial commencement made

3b-i) Bug fixes, Downtimes, Content Changes

Bug fixes, Downtimes, Content Changes: ehealth systems are often dynamic systems. A description of changes to methods therefore also includes important changes made on the intervention or comparator during the trial (e.g., major bug fixes or changes in the functionality or content) (5-iii) and other “unexpected events” that may have influenced study design such as staff changes, system failures/downtimes, etc. [2].

	1	2	3	4	5	
subitem not at all important	○	◉	○	○	○	essential

Auswahl löschen

Does your paper address subitem 3b-i?

Copy and paste relevant sections from the manuscript (include quotes in quotation marks "like this" to indicate direct quotes from your manuscript), or elaborate on this item by providing additional information not in the ms, or briefly explain why the item is not applicable/relevant for your study

Meine Antwort

4a) Eligibility criteria for participants

Does your paper address CONSORT subitem 4a? *

Copy and paste relevant sections from the manuscript (include quotes in quotation marks "like this" to indicate direct quotes from your manuscript), or elaborate on this item by providing additional information not in the ms, or briefly explain why the item is not applicable/relevant for your study

"The present study included Arabic-speaking adults from different countries who were seeking help online for posttraumatic stress and depressive symptoms. As an inclusion criterion, all participants were required to be able to speak, read, and write standard Arabic. Individuals were excluded if they self-reported any of the following in the screening battery: age below 18 years; no private email address or access to a computer and Internet; simultaneously receiving psychotherapeutic treatment elsewhere or planning psychotherapeutic treatment within the next four weeks; or suffering from severe depressive symptoms (Beck Depression Inventory II ≥ 45). After successfully passing the screening battery, a clinical interview was conducted, in which interviewers checked whether participants met diagnostic criteria for a depressive disorder or PTSD according to the DSM-5, assessed using the Structured Clinical Interview for DSM-5 Disorders [SCID-5 Clinical Version], as a requirement for participation in one of the offered treatments. If the diagnostic criteria were not met, participants were excluded. In the interview, participants were further screened for symptoms of mania or hypomania, psychotic experiences, risk of suicide, drug and alcohol use, and current risk of re-traumatization (i.e. still living together with the perpetrator). We further excluded participants who reported psychotic tendencies (i.e. at least one delusion or one hallucination symptom, lifetime), manic or hypomanic episodes, a high risk of suicide (i.e. serious suicide attempts within the last three years or a current intent), dependency on or abuse of drugs or alcohol with current use, or a current danger of re-traumatization. In addition, interviewers checked whether any participants receiving psychopharmacological treatment were on a stable dose and whether participants had not completed our treatment program within the last months. Again, participants failing to meet these criteria were excluded."

4a-i) Computer / Internet literacy

Computer / Internet literacy is often an implicit “de facto” eligibility criterion - this should be explicitly clarified.

	1	2	3	4	5	
subitem not at all important	(●)	○	○	○	○	essential

Auswahl löschen

Does your paper address subitem 4a-i?

Copy and paste relevant sections from the manuscript (include quotes in quotation marks "like this" to indicate direct quotes from your manuscript), or elaborate on this item by providing additional information not in the ms, or briefly explain why the item is not applicable/relevant for your study

Meine Antwort

4a-ii) Open vs. closed, web-based vs. face-to-face assessments:

Open vs. closed, web-based vs. face-to-face assessments: Mention how participants were recruited (online vs. offline), e.g., from an open access website or from a clinic, and clarify if this was a purely web-based trial, or there were face-to-face components (as part of the intervention or for assessment), i.e., to what degree got the study team to know the participant. In online-only trials, clarify if participants were quasi-anonymous and whether having multiple identities was possible or whether technical or logistical measures (e.g., cookies, email confirmation, phone calls) were used to detect/prevent these.

	1	2	3	4	5	
subitem not at all important	○	○	○	◉	○	essential

Auswahl löschen

Does your paper address subitem 4a-ii? *

Copy and paste relevant sections from the manuscript (include quotes in quotation marks "like this" to indicate direct quotes from your manuscript), or elaborate on this item by providing additional information not in the ms, or briefly explain why the item is not applicable/relevant for your study

"Participants were recruited through the program’s website (Ilajnafsy (العلاج النفسي): Arabic for psychotherapy, https://ilajnafsy.bzfo.de), word-of-mouth recommendation and social media (i.e. facebook)."

4a-iii) Information giving during recruitment

Information given during recruitment. Specify how participants were briefed for recruitment and in the informed consent procedures (e.g., publish the informed consent documentation as appendix, see also item X26), as this information may have an effect on user self-selection, user expectation and may also bias results.

	1	2	3	4	5	
subitem not at all important	○	◉	○	○	○	essential

Auswahl löschen

Does your paper address subitem 4a-iii?

Copy and paste relevant sections from the manuscript (include quotes in quotation marks "like this" to indicate direct quotes from your manuscript), or elaborate on this item by providing additional information not in the ms, or briefly explain why the item is not applicable/relevant for your study

Meine Antwort

4b) Settings and locations where the data were collected

Does your paper address CONSORT subitem 4b? *

Copy and paste relevant sections from the manuscript (include quotes in quotation marks "like this" to indicate direct quotes from your manuscript), or elaborate on this item by providing additional information not in the ms, or briefly explain why the item is not applicable/relevant for your study

"Primary and secondary outcome measures were self-reported and administered online in a password-protected area."

4b-i) Report if outcomes were (self-)assessed through online questionnaires

Clearly report if outcomes were (self-)assessed through online questionnaires (as common in web-based trials) or otherwise.

	1	2	3	4	5	
subitem not at all important	○	○	○	○	◉	essential

Auswahl löschen

Does your paper address subitem 4b-i? *

Copy and paste relevant sections from the manuscript (include quotes in quotation marks "like this" to indicate direct quotes from your manuscript), or elaborate on this item by providing additional information not in the ms, or briefly explain why the item is not applicable/relevant for your study

"Primary and secondary outcome measures were self-reported and administered online in a password-protected area. "

4b-ii) Report how institutional affiliations are displayed

Report how institutional affiliations are displayed to potential participants [on ehealth media], as affiliations with prestigious hospitals or universities may affect volunteer rates, use, and reactions with regards to an intervention.(Not a required item – describe only if this may bias results)

	1	2	3	4	5	
subitem not at all important	○	◉	○	○	○	essential

Auswahl löschen

Does your paper address subitem 4b-ii?

Copy and paste relevant sections from the manuscript (include quotes in quotation marks "like this" to indicate direct quotes from your manuscript), or elaborate on this item by providing additional information not in the ms, or briefly explain why the item is not applicable/relevant for your study

Meine Antwort

5) The interventions for each group with sufficient details to allow replication, including how and when they were actually administered

5-i) Mention names, credential, affiliations of the developers, sponsors, and owners

Mention names, credential, affiliations of the developers, sponsors, and owners [6] (if authors/evaluators are owners or developer of the software, this needs to be declared in a “Conflict of interest” section or mentioned elsewhere in the manuscript).

	1	2	3	4	5	
subitem not at all important	○	○	●	○	○	essential

Auswahl löschen

Does your paper address subitem 5-i?

Copy and paste relevant sections from the manuscript (include quotes in quotation marks "like this" to indicate direct quotes from your manuscript), or elaborate on this item by providing additional information not in the ms, or briefly explain why the item is not applicable/relevant for your study

Meine Antwort

5-ii) Describe the history/development process

Describe the history/development process of the application and previous formative evaluations (e.g., focus groups, usability testing), as these will have an impact on adoption/use rates and help with interpreting results.

	1	2	3	4	5	
subitem not at all important	○	◉	○	○	○	essential

Auswahl löschen

Does your paper address subitem 5-ii?

Copy and paste relevant sections from the manuscript (include quotes in quotation marks "like this" to indicate direct quotes from your manuscript), or elaborate on this item by providing additional information not in the ms, or briefly explain why the item is not applicable/relevant for your study

Meine Antwort

5-iii) Revisions and updating

Revisions and updating. Clearly mention the date and/or version number of the application/intervention (and comparator, if applicable) evaluated, or describe whether the intervention underwent major changes during the evaluation process, or whether the development and/or content was “frozen” during the trial. Describe dynamic components such as news feeds or changing content which may have an impact on the replicability of the intervention (for unexpected events see item 3b).

	1	2	3	4	5	
subitem not at all important	○	◉	○	○	○	essential

Auswahl löschen

Does your paper address subitem 5-iii?

Copy and paste relevant sections from the manuscript (include quotes in quotation marks "like this" to indicate direct quotes from your manuscript), or elaborate on this item by providing additional information not in the ms, or briefly explain why the item is not applicable/relevant for your study

Meine Antwort

5-iv) Quality assurance methods

Provide information on quality assurance methods to ensure accuracy and quality of information provided [1], if applicable.

	1	2	3	4	5	
subitem not at all important	○	○	◉	○	○	essential

Auswahl löschen

Does your paper address subitem 5-iv?

Copy and paste relevant sections from the manuscript (include quotes in quotation marks "like this" to indicate direct quotes from your manuscript), or elaborate on this item by providing additional information not in the ms, or briefly explain why the item is not applicable/relevant for your study

Meine Antwort

5-v) Ensure replicability by publishing the source code, and/or providing screenshots/screen-capture video, and/or providing flowcharts of the algorithms used

Ensure replicability by publishing the source code, and/or providing screenshots/screen-capture video, and/or providing flowcharts of the algorithms used. Replicability (i.e., other researchers should in principle be able to replicate the study) is a hallmark of scientific reporting.

	1	2	3	4	5	
subitem not at all important	○	◉	○	○	○	essential

Auswahl löschen

Does your paper address subitem 5-v?

Copy and paste relevant sections from the manuscript (include quotes in quotation marks "like this" to indicate direct quotes from your manuscript), or elaborate on this item by providing additional information not in the ms, or briefly explain why the item is not applicable/relevant for your study

Meine Antwort

5-vi) Digital preservation

Digital preservation: Provide the URL of the application, but as the intervention is likely to change or disappear over the course of the years; also make sure the intervention is archived (Internet Archive, webcitation.org, and/or publishing the source code or screenshots/videos alongside the article). As pages behind login screens cannot be archived, consider creating demo pages which are accessible without login.

	1	2	3	4	5	
subitem not at all important	○	◉	○	○	○	essential

Auswahl löschen

Does your paper address subitem 5-vi?

Copy and paste relevant sections from the manuscript (include quotes in quotation marks "like this" to indicate direct quotes from your manuscript), or elaborate on this item by providing additional information not in the ms, or briefly explain why the item is not applicable/relevant for your study

Meine Antwort

5-vii) Access

Access: Describe how participants accessed the application, in what setting/context, if they had to pay (or were paid) or not, whether they had to be a member of specific group. If known, describe how participants obtained “access to the platform and Internet” [1]. To ensure access for editors/reviewers/readers, consider to provide a “backdoor” login account or demo mode for reviewers/readers to explore the application (also important for archiving purposes, see vi).

	1	2	3	4	5	
subitem not at all important	○	○	○	●	○	essential

Auswahl löschen

Does your paper address subitem 5-vii? *

Copy and paste relevant sections from the manuscript (include quotes in quotation marks "like this" to indicate direct quotes from your manuscript), or elaborate on this item by providing additional information not in the ms, or briefly explain why the item is not applicable/relevant for your study

"Participants were recruited through the program’s website (Ilajnafsy (العلاج النفسي): Arabic for psychotherapy, https://ilajnafsy.bzfo.de), word-of-mouth recommendation and social media (i.e. facebook)."

5-viii) Mode of delivery, features/functionalities/components of the intervention and comparator, and the theoretical framework

Describe mode of delivery, features/functionalities/components of the intervention and comparator, and the theoretical framework [6] used to design them (instructional strategy [1], behaviour change techniques, persuasive features, etc., see e.g., [7, 8] for terminology). This includes an in-depth description of the content (including where it is coming from and who developed it) [1],” whether [and how] it is tailored to individual circumstances and allows users to track their progress and receive feedback” [6]. This also includes a description of communication delivery channels and – if computer-mediated communication is a component – whether communication was synchronous or asynchronous [6]. It also includes information on presentation strategies [1], including page design principles, average amount of text on pages, presence of hyperlinks to other resources, etc. [1].

	1	2	3	4	5	
subitem not at all important	○	○	○	○	◉	essential

Auswahl löschen

Does your paper address subitem 5-viii? *

Copy and paste relevant sections from the manuscript (include quotes in quotation marks "like this" to indicate direct quotes from your manuscript), or elaborate on this item by providing additional information not in the ms, or briefly explain why the item is not applicable/relevant for your study

" The two treatments were based on an internet-based cognitive behavioral treatment approach for PTSD [Interapy]. An overview of the procedure and writing examples of both treatment conditions can be found in the supplementary material, lingustic and cultural changes made to the original protocol can be found in the manuscript. Both treatments consisted of twice-weekly 45-min structured writing assignments in the form of letters over a period of approximately three weeks (approximately two letters per week).
Exposure treatment (EXPO): Besides the introductory part, the exposure treatment included two different phases. In the first phase of self-confrontation, participants were instructed to write four letters about the traumatic event and their related thoughts, fears, and physiological reactions. They were asked to describe sensory perceptions in detail and to focus on the most distressing situation of the trauma. We also included a section on how traumatic events are processed and why symptoms are maintained, as well as how exposure treatment could help, in order to make the condition comparable in length with the cognitive restructuring treatment. In the second phase – the social sharing phase – participants were asked to write two letters to summarize their memories of the trauma and to consider how they were going to deal with the trauma in the future. The phase of social sharing focused on a symbolic farewell letter that participants were instructed to address to themselves or to a significant other.
Cognitive restructuring treatment (CR): Besides the introductory part, the cognitive restructuring treatment included two different phases. The first phase of cognitive restructuring encompassed four letters to reflect on automatic dysfunctional cognitions and to adjust unrealistic assumptions (for example guilt). Participants were instructed to write a letter to a hypothetical friend who had experienced the same traumatic event, without necessarily going into details of the traumatic experience. Compared to the original Interapy protocol, in which the cognitive restructuring phase was implemented after the exposure phase, we had to adapt the cognitive restructuring part to enable participants to begin this phase without the prior knowledge gained through the exposure phase. Therefore, detailed information was provided in advance regarding the impact of traumatic events, i.e., how traumatic experiences can influence thoughts and beliefs about oneself, other people, and the world, and how these unhelpful thoughts/beliefs lead to emotions like guilt and shame. In addition, to encourage participants to identify, challenge, and modify unhelpful beliefs, numerous reflective questions (e.g., Why did the traumatic event occur? What evidence/counterevidence is there that your friend is responsible for what happened?) were included before starting with the writing assignments. The second, social sharing phase was identical to that described for the exposure treatment.
Waitlist control group (WAIT): The comparison with the waitlist control group was conducted to account for the potential influence of elapsed time and quantify the efficacy of the two treatments. After a waiting period of three weeks, the waitlist participants received an email invitation to start the program. Before starting one of the two treatments (to which they had been randomized in advance), they completed all symptom questionnaires. "

5-ix) Describe use parameters

Describe use parameters (e.g., intended “doses” and optimal timing for use). Clarify what instructions or recommendations were given to the user, e.g., regarding timing, frequency, heaviness of use, if any, or was the intervention used ad libitum.

	1	2	3	4	5	
subitem not at all important	○	○	◉	○	○	essential

Auswahl löschen

Does your paper address subitem 5-ix?

Copy and paste relevant sections from the manuscript (include quotes in quotation marks "like this" to indicate direct quotes from your manuscript), or elaborate on this item by providing additional information not in the ms, or briefly explain why the item is not applicable/relevant for your study

Meine Antwort

5-x) Clarify the level of human involvement

Clarify the level of human involvement (care providers or health professionals, also technical assistance) in the e-intervention or as co-intervention (detail number and expertise of professionals involved, if any, as well as “type of assistance offered, the timing and frequency of the support, how it is initiated, and the medium by which the assistance is delivered”. It may be necessary to distinguish between the level of human involvement required for the trial, and the level of human involvement required for a routine application outside of a RCT setting (discuss under item 21 – generalizability).

	1	2	3	4	5	
subitem not at all important	○	○	○	◉	○	essential

Auswahl löschen

Does your paper address subitem 5-x?

Copy and paste relevant sections from the manuscript (include quotes in quotation marks "like this" to indicate direct quotes from your manuscript), or elaborate on this item by providing additional information not in the ms, or briefly explain why the item is not applicable/relevant for your study

Meine Antwort

5-xi) Report any prompts/reminders used

Report any prompts/reminders used: Clarify if there were prompts (letters, emails, phone calls, SMS) to use the application, what triggered them, frequency etc. It may be necessary to distinguish between the level of prompts/reminders required for the trial, and the level of prompts/reminders for a routine application outside of a RCT setting (discuss under item 21 – generalizability).

	1	2	3	4	5	
subitem not at all important	○	○	○	●	○	essential

Auswahl löschen

Does your paper address subitem 5-xi? *

Copy and paste relevant sections from the manuscript (include quotes in quotation marks "like this" to indicate direct quotes from your manuscript), or elaborate on this item by providing additional information not in the ms, or briefly explain why the item is not applicable/relevant for your study

"Participants received automated emails when they were supposed to log in to the portal (e.g. when there was a letter or message from the counselor, or a writing assignment was due). Additionally, they received automated reminders at each step of the procedure if they were inactive: During the registration and screening process, participants received automated reminders after three and after seven days of inactivity and were excluded after 14 days of inactivity. During the interview process, they were reminded after three, seven and 14 days and excluded after 21 days of inactivity. Participants who had already been included and allocated to one of the conditions received an automated email after three and seven days if they did not respond to the automated invitation (waitlist control group) or if they did not complete the letters on the chosen dates (treatment groups). Additionally, if participants had not responded to the two reminder messages, the counselor contacted them by telephone (if possible) to encourage them to continue. If they could not be reached by telephone, a message was sent, including a deadline for a response. After 14 days of non-response, participants were considered as dropouts."

5-xii) Describe any co-interventions (incl. training/support)

Describe any co-interventions (incl. training/support): Clearly state any interventions that are provided in addition to the targeted eHealth intervention, as ehealth intervention may not be designed as stand-alone intervention. This includes training sessions and support [1]. It may be necessary to distinguish between the level of training required for the trial, and the level of training for a routine application outside of a RCT setting (discuss under item 21 – generalizability.

	1	2	3	4	5	
subitem not at all important	○	○	○	◉	○	essential

Auswahl löschen

Does your paper address subitem 5-xii? *

Copy and paste relevant sections from the manuscript (include quotes in quotation marks "like this" to indicate direct quotes from your manuscript), or elaborate on this item by providing additional information not in the ms, or briefly explain why the item is not applicable/relevant for your study

"Ten native Arabic-speaking counselors living in Egypt or Germany performed the treatments. All counselors had a diploma in psychology or psychology-related disciplines (e.g., social work, counseling, psychotherapy) and/or extensive work experience. Counselors received continuous training covering information about and treatment options for PTSD, fundamentals and technical aspects of internet-based treatments, specific treatment rationales, providing feedback, and dealing with challenging situations. Furthermore, all counselors attended regular supervision meetings held by experienced psychotherapists. Support for participants by email or telephone was limited to emergency situations (i.e., in cases of suicidality, dropout), technical support, or reminders to continue treatment."

6a) Completely defined pre-specified primary and secondary outcome measures, including how and when they were assessed

Does your paper address CONSORT subitem 6a? *

Copy and paste relevant sections from the manuscript (include quotes in quotation marks "like this" to indicate direct quotes from your manuscript), or elaborate on this item by providing additional information not in the ms, or briefly explain why the item is not applicable/relevant for your study

"Sociodemographic characteristics and exposure to traumatic events were assessed in the screening test battery only. For this purpose, we used items from the Harvard Trauma Questionnaire [HTQ], the Posttraumatic Diagnostic Scale [PDS] and the Life Events Checklist for DSM-5 [LEC-5], with a total of 25 items asking about exposure to various potentially traumatic events, as well as the extended version of the LEC-5 asking about further details of the most distressing event. All outcome measures were assessed as part of the screening test battery (baseline assessment, T1) and at the end of treatment/waiting time (post-assessment, T5). Questionnaires asking about satisfaction with the treatment were administered after participants had completed one of the treatment conditions. The PTSD Checklist for DSM-5 [PCL-5;] was additionally administered at three measurement time points during treatment (T2: assessment immediately before starting treatment; T3: assessment after two letters; T4: assessment after four letters). In the waitlist control group, intermediate measures were administered in correspondence with the treatment groups (i.e. participants were invited to complete the PCL-5 every week during the waiting period; T2: immediately before starting the waiting time; T3: assessment after one week; T4: assessment after two weeks).
Primary outcome measure:
Symptoms of posttraumatic stress in the past month were assessed using the PCL-5.
Secondary outcome measure:
Posttraumatic maladaptive beliefs about the world, others, and the self were assessed using the self-report Posttraumatic Maladaptive Beliefs Scale.
Trauma-related guilt cognitions were assessed using the guilt cognitions scale of the self-report Trauma-Related Guilt Inventory.
Anxiety symptom severity was measured using the self-report Arabic version of the Generalized Anxiety Disorder Scale-7.
Depressive symptom severity was assessed using the self-report Patient Health Questionnaire-9.
Somatoform symptom severity was measured using the self-report Patient Health Questionnaire-15.
Quality of life was assessed using the self-report EUROHIS-QOL 8-item index, an adapted version of the WHOQOL-100 and the WHOQOL-BREF

6a-i) Online questionnaires: describe if they were validated for online use and apply CHERRIES items to describe how the questionnaires were designed/deployed

If outcomes were obtained through online questionnaires, describe if they were validated for online use and apply CHERRIES items to describe how the questionnaires were designed/deployed [9].

	1	2	3	4	5	
subitem not at all important	○	○	◉	○	○	essential

Auswahl löschen

Does your paper address subitem 6a-i?

Copy and paste relevant sections from manuscript text

Meine Antwort

6a-ii) Describe whether and how “use” (including intensity of use/dosage) was defined/measured/monitored

Describe whether and how “use” (including intensity of use/dosage) was defined/measured/monitored (logins, logfile analysis, etc.). Use/adoption metrics are important process outcomes that should be reported in any ehealth trial.

	1	2	3	4	5	
subitem not at all important	○	◉	○	○	○	essential

Auswahl löschen

Does your paper address subitem 6a-ii?

Copy and paste relevant sections from manuscript text

Meine Antwort

6a-iii) Describe whether, how, and when qualitative feedback from participants was obtained

Describe whether, how, and when qualitative feedback from participants was obtained (e.g., through emails, feedback forms, interviews, focus groups).

	1	2	3	4	5	
subitem not at all important	○	○	○	●	○	essential

Auswahl löschen

Does your paper address subitem 6a-iii?

Copy and paste relevant sections from manuscript text

Meine Antwort

6b) Any changes to trial outcomes after the trial commenced, with reasons

Does your paper address CONSORT subitem 6b? *

Copy and paste relevant sections from the manuscript (include quotes in quotation marks "like this" to indicate direct quotes from your manuscript), or elaborate on this item by providing additional information not in the ms, or briefly explain why the item is not applicable/relevant for your study

No changes after trial commenced

7a) How sample size was determined

NPT: When applicable, details of whether and how the clustering by care provides or centers was addressed

7a-i) Describe whether and how expected attrition was taken into account when calculating the sample size

Describe whether and how expected attrition was taken into account when calculating the sample size.

	1	2	3	4	5	
subitem not at all important	○	○	◉	○	○	essential

Auswahl löschen

Does your paper address subitem 7a-i?

Copy and paste relevant sections from manuscript title (include quotes in quotation marks "like this" to indicate direct quotes from your manuscript), or elaborate on this item by providing additional information not in the ms, or briefly explain why the item is not applicable/relevant for your study

Meine Antwort

7b) When applicable, explanation of any interim analyses and stopping guidelines

Does your paper address CONSORT subitem 7b? *

Copy and paste relevant sections from the manuscript (include quotes in quotation marks "like this" to indicate direct quotes from your manuscript), or elaborate on this item by providing additional information not in the ms, or briefly explain why the item is not applicable/relevant for your study

this item is not applicable

8a) Method used to generate the random allocation sequence

NPT: When applicable, how care providers were allocated to each trial group

Does your paper address CONSORT subitem 8a? *

Copy and paste relevant sections from the manuscript (include quotes in quotation marks "like this" to indicate direct quotes from your manuscript), or elaborate on this item by providing additional information not in the ms, or briefly explain why the item is not applicable/relevant for your study

"Randomization was performed using a block randomization with variable block sizes of six, nine and twelve blocks. The allocation schedule was created with the R package Blockrand and embedded in the web portal."

8b) Type of randomisation; details of any restriction (such as blocking and block size)

Does your paper address CONSORT subitem 8b? *

Copy and paste relevant sections from the manuscript (include quotes in quotation marks "like this" to indicate direct quotes from your manuscript), or elaborate on this item by providing additional information not in the ms, or briefly explain why the item is not applicable/relevant for your study

"block randomization with variable block sizes of six, nine and twelve blocks"

9) Mechanism used to implement the random allocation sequence (such as sequentially numbered containers), describing any steps taken to conceal the sequence until interventions were assigned

Does your paper address CONSORT subitem 9? *

Copy and paste relevant sections from the manuscript (include quotes in quotation marks "like this" to indicate direct quotes from your manuscript), or elaborate on this item by providing additional information not in the ms, or briefly explain why the item is not applicable/relevant for your study

"The allocation schedule was created with the R package Blockrand and embedded in the web portal."

10) Who generated the random allocation sequence, who enrolled participants, and who assigned participants to interventions

Does your paper address CONSORT subitem 10? *

Copy and paste relevant sections from the manuscript (include quotes in quotation marks "like this" to indicate direct quotes from your manuscript), or elaborate on this item by providing additional information not in the ms, or briefly explain why the item is not applicable/relevant for your study

"Allocation to one of the three conditions was performed invisibly and automatically on the web portal itself and was thus concealed, i.e. participants, counselors, and researchers had no prior knowledge of, and therefore no control over, the group to which a participant would be allocated."

11a) If done, who was blinded after assignment to interventions (for example, participants, care providers, those assessing outcomes) and how

NPT: Whether or not administering co-interventions were blinded to group assignment

11a-i) Specify who was blinded, and who wasn't

Specify who was blinded, and who wasn't. Usually, in web-based trials it is not possible to blind the participants [1, 3] (this should be clearly acknowledged), but it may be possible to blind outcome assessors, those doing data analysis or those administering co-interventions (if any).

	1	2	3	4	5	
subitem not at all important	○	○	○	◉	○	essential

Auswahl löschen

Does your paper address subitem 11a-i? *

Copy and paste relevant sections from the manuscript (include quotes in quotation marks "like this" to indicate direct quotes from your manuscript), or elaborate on this item by providing additional information not in the ms, or briefly explain why the item is not applicable/relevant for your study

"Due to the nature of the provided treatments, participants and counselors could not be blinded regarding the treatment condition received."

11a-ii) Discuss e.g., whether participants knew which intervention was the "intervention of interest" and which one was the "comparator"

Informed consent procedures (4a-ii) can create biases and certain expectations - discuss e.g., whether participants knew which intervention was the "intervention of interest" and which one was the "comparator".

	1	2	3	4	5	
subitem not at all important	○	○	○	◉	○	essential

Auswahl löschen

Does your paper address subitem 11a-ii?

Copy and paste relevant sections from the manuscript (include quotes in quotation marks "like this" to indicate direct quotes from your manuscript), or elaborate on this item by providing additional information not in the ms, or briefly explain why the item is not applicable/relevant for your study

Meine Antwort

11b) If relevant, description of the similarity of interventions

(this item is usually not relevant for ehealth trials as it refers to similarity of a placebo or sham intervention to a active medication/intervention)

Does your paper address CONSORT subitem 11b? *

Copy and paste relevant sections from the manuscript (include quotes in quotation marks "like this" to indicate direct quotes from your manuscript), or elaborate on this item by providing additional information not in the ms, or briefly explain why the item is not applicable/relevant for your study

"The two treatments were based on an internet-based cognitive behavioral treatment approach for PTSD [Interapy]. An overview of the procedure and writing examples of both treatment conditions can be found in the supplementary material. The protocols were translated into Modern Standard Arabic. To obtain linguistically and culturally appropriate protocols, we made the following changes: 1) different versions for female and male participants regarding particularities in the Arabic language; 2) strengthening the advice to not mention real names or places involved in the traumatic event due to basic precautionary measures; 3) use of pictorial metaphors (i.e. scar/wound metaphor, linen cupboard metaphor for PTSD) to explain the purpose and process of trauma treatment in a less technical way; and 4) use of an encouraging and motivational but directive writing style. If needed, e.g., the participant expressed a high level of faith, counselors could include quotes from the Qur´an. In addition, the layout of the protocols and as well as technical descriptions within the protocols were adapted to fit the format of the web portal (e.g., participants were instructed to use the online planner). Both treatments consisted of twice-weekly 45-min structured writing assignments in the form of letters over a period of approximately three weeks (approximately two letters per week). The writing sessions were planned, and participants were instructed to plan the date and hour when they would write each letter. After receiving each letter from a participant, the counselors provided individual feedback and instructions for the next letter within two working days. The feedback and instructions consisted of standard examples that were tailored to the participants´ individual needs and the content of the previous letter(s). Both treatments began with an introduction by the counselor, providing information on writing treatment in general, the procedure of the treatment in detail, and psychoeducational information on PTSD. As the counselors already had knowledge of the traumatic event to be addressed in the treatment through the interview report, they could directly refer to the most distressing traumatic event and/or associated dysfunctional thoughts and feelings in their first letter. At the beginning of each module, psychoeducational information on the specific treatment phase was given. In both treatments, a final letter was sent at the end of treatment, in which the counselor summarized the participant’s progress during treatment. "

12a) Statistical methods used to compare groups for primary and secondary outcomes

NPT: When applicable, details of whether and how the clustering by care providers or centers was addressed

Does your paper address CONSORT subitem 12a? *

Copy and paste relevant sections from the manuscript (include quotes in quotation marks "like this" to indicate direct quotes from your manuscript), or elaborate on this item by providing additional information not in the ms, or briefly explain why the item is not applicable/relevant for your study

"Analyses were performed using R statistical software, version 4.2.2 and Mplus statistical modeling software, version 8. All three conditions were compared regarding baseline characteristics to see if randomization worked properly. We examined the association of treatment condition with the proportion of participants who did not start treatment vs. wrote at least one letter (non-starters vs. starters). Non-starters and starters were further compared regarding sociodemographic, trauma-related, and clinical characteristics reported at baseline. Similarly, we investigated the association of treatment condition with the proportion of participants who stopped treatment before completing all six letters vs. those who completed all six letters (dropouts vs. completers). Furthermore, in both treatment conditions, dropouts and completers were compared regarding sociodemographic, trauma-related, and clinical characteristics reported at baseline. In addition, the duration of both treatment conditions (in days) was compared between both treatment conditions. Results of the post-treatment evaluation questions, as markers of treatment satisfaction, were compared between the two treatment conditions. All of the aforementioned group differences were investigated using Welch or Chi-square tests. If assumptions for the Welch tests were not met, Kruskal-Wallis or Mann-Whitney tests were applied. Fisher's exact test was used as an alternative for Chi-square test.(...). Treatment-associated changes in primary and secondary outcome measures across different measurement time points were modeled using multi-group latent change models. Models were estimated with the robust maximum likelihood estimator. The rate of change is determined under the assumption that the score at a specific measurement time point after the initial assessment is composed of the initial score and the difference between the initial score and the score obtained at the specific measurement time point after the initial assessment (i.e. the post-assessment score) [i.e. 54]. Thus, the rate of change between measurement time points is directly modelled in the form of the change score. The mean of the change scores represents the average change (decrease or increase) between two measurements within each condition in units of the questionnaire. Between-group effects are represented by the differences between the group-specific mean change scores. Within-group effect sizes were computed by dividing the mean change scores by their standard deviation for each group. Between-group effect sizes were computed by dividing the mean difference between the mean change scores of two groups by the pooled standard deviation. All results on treatment-associated changes were pooled across multiple imputed data sets. Bonferroni correction was applied to keep the error rate at .05 for within-group changes and between-group differences. Therefore, a P value of < .005 (adjusted for eleven treatment outcomes) was considered statistically significant for within- and between-group comparisons. To assess reliable changes in individual posttraumatic stress symptom severity between baseline and post-assessment in all three conditions, we calculated the reliable change index for each participant, using the test-retest reliability of r=0.82 for the PCL-5 and the standard deviation at baseline of the present sample (SD of 13.28, pooled across imputed data sets). According to this calculation, changes in posttraumatic stress symptom severity were considered as statistically significant if the difference between baseline and post-assessment exceeded 16 points in the PCL-5 (α=.05). Proportions of participants with reliable improvement (minimum 16-point decrease) or deterioration (minimum 16-point increase) were calculated. Furthermore, we calculated rates of remitted participants, i.e. participants with a baseline PCL-5 value ≥ 23, as an indicator of caseness (suffering from PTSD), and a post-assessment PCL-5 value < 23. The cut-off value of 23 was chosen based on a study with Arabic-speaking people. Results of the study showed that the PCL-5 achieved the best balance between [illegible]

sensitivity and specificity in the Arabic-speaking sample when this cut-off was used. In addition, proportions of participants who experienced both reliable and clinically significant improvement (RCSI) were determined. The association between all three conditions and the proportion of participants with reliable change, experience of remission, and RCSI was examined using Chi-square tests, which were pooled across all imputed data sets]. Analyses of treatment-associated change were performed on the intention-to-treat (ITT) and the completer sample. Completers in both treatment conditions were defined as those participants who had completed all six letters. In the WAIT condition, completers were defined as participants who had completed all questionnaires of the post-assessment (T5)."

12a-i) Imputation techniques to deal with attrition / missing values

Imputation techniques to deal with attrition / missing values: Not all participants will use the intervention/comparator as intended and attrition is typically high in ehealth trials. Specify how participants who did not use the application or dropped out from the trial were treated in the statistical analysis (a complete case analysis is strongly discouraged, and simple imputation techniques such as LOCF may also be problematic [4]).

	1	2	3	4	5	
subitem not at all important	○	○	○	○	◉	essential

Auswahl löschen

Does your paper address subitem 12a-i? *

Copy and paste relevant sections from the manuscript (include quotes in quotation marks "like this" to indicate direct quotes from your manuscript), or elaborate on this item by providing additional information not in the ms, or briefly explain why the item is not applicable/relevant for your study

"To deal with missing data, multiple imputation (100 imputed data sets, 50 iterations) for primary and secondary outcome measures was performed with the R package MICE. All outcome measures were used in the imputation model. Predictive mean matching on the level of sum scores was applied for all variables except for the overall sum score of the PCL-5. For the overall sum scores of the PCL-5 at all measurement time points, passive imputation was used to account for the dependency of the overall PCL-5 sum score on the sum scores of the symptom clusters. Multiple imputation was conducted separately for each of the three conditions. "

12b) Methods for additional analyses, such as subgroup analyses and adjusted analyses

Does your paper address CONSORT subitem 12b? *

Copy and paste relevant sections from the manuscript (include quotes in quotation marks "like this" to indicate direct quotes from your manuscript), or elaborate on this item by providing additional information not in the ms, or briefly explain why the item is not applicable/relevant for your study

"A sensitivity analysis was conducted to investigate whether deviations from the missing at random (MAR) assumption would impact the conclusions drawn from the results calculated under the assumptions that data are MAR. For the primary outcome measured with the PCL-5, three different conditions were modeled for the ITT sample. Individual imputed scores at each measurement time point (after the baseline assessment) were increased by 25%, 50%, and 75% for all participants."

X26) REB/IRB Approval and Ethical Considerations [recommended as subheading under "Methods"] (not a CONSORT item)

X26-i) Comment on ethics committee approval

	1	2	3	4	5	
subitem not at all important	○	○	○	◉	○	essential

Auswahl löschen

Does your paper address subitem X26-i?

Copy and paste relevant sections from the manuscript (include quotes in quotation marks "like this" to indicate direct quotes from your manuscript), or elaborate on this item by providing additional information not in the ms, or briefly explain why the item is not applicable/relevant for your study

Meine Antwort

x26-ii) Outline informed consent procedures

Outline informed consent procedures e.g., if consent was obtained offline or online (how? Checkbox, etc.?), and what information was provided (see 4a-ii). See [6] for some items to be included in informed consent documents.

	1	2	3	4	5	
subitem not at all important	○	○	◉	○	○	essential

Auswahl löschen

Does your paper address subitem X26-ii?

Copy and paste relevant sections from the manuscript (include quotes in quotation marks "like this" to indicate direct quotes from your manuscript), or elaborate on this item by providing additional information not in the ms, or briefly explain why the item is not applicable/relevant for your study

Meine Antwort

X26-iii) Safety and security procedures

Safety and security procedures, incl. privacy considerations, and any steps taken to reduce the likelihood or detection of harm (e.g., education and training, availability of a hotline)

	1	2	3	4	5	
subitem not at all important	○	○	○	◉	○	essential

Auswahl löschen

Does your paper address subitem X26-iii?

Copy and paste relevant sections from the manuscript (include quotes in quotation marks "like this" to indicate direct quotes from your manuscript), or elaborate on this item by providing additional information not in the ms, or briefly explain why the item is not applicable/relevant for your study

Meine Antwort

RESULTS

13a) For each group, the numbers of participants who were randomly assigned, received intended treatment, and were analysed for the primary outcome

NPT: The number of care providers or centers performing the intervention in each group and the number of patients treated by each care provider in each center

Does your paper address CONSORT subitem 13a? *

Copy and paste relevant sections from the manuscript (include quotes in quotation marks "like this" to indicate direct quotes from your manuscript), or elaborate on this item by providing additional information not in the ms, or briefly explain why the item is not applicable/relevant for your study

see flow chart in manuscript

13b) For each group, losses and exclusions after randomisation, together with reasons

Does your paper address CONSORT subitem 13b? (NOTE: Preferably, this is shown in a CONSORT flow diagram) *

Copy and paste relevant sections from the manuscript (include quotes in quotation marks "like this" to indicate direct quotes from your manuscript), or elaborate on this item by providing additional information not in the ms, or briefly explain why the item is not applicable/relevant for your study

see flow chart in manuscript, reasons are unknown

13b-i) Attrition diagram

Strongly recommended: An attrition diagram (e.g., proportion of participants still logging in or using the intervention/comparator in each group plotted over time, similar to a survival curve) or other figures or tables demonstrating usage/dose/engagement.

	1	2	3	4	5	
subitem not at all important	○	○	●	○	○	essential

Auswahl löschen

Does your paper address subitem 13b-i?

Copy and paste relevant sections from the manuscript or cite the figure number if applicable (include quotes in quotation marks "like this" to indicate direct quotes from your manuscript), or elaborate on this item by providing additional information not in the ms, or briefly explain why the item is not applicable/relevant for your study

Meine Antwort

14a) Dates defining the periods of recruitment and follow-up

Does your paper address CONSORT subitem 14a? *

Copy and paste relevant sections from the manuscript (include quotes in quotation marks "like this" to indicate direct quotes from your manuscript), or elaborate on this item by providing additional information not in the ms, or briefly explain why the item is not applicable/relevant for your study

"Recruitment took place between 9th of February 2021 and 13th of December 2022."

14a-i) Indicate if critical “secular events” fell into the study period

Indicate if critical “secular events” fell into the study period, e.g., significant changes in Internet resources available or “changes in computer hardware or Internet delivery resources”

	1	2	3	4	5	
subitem not at all important	○	○	○	○	○	essential

Does your paper address subitem 14a-i?

Copy and paste relevant sections from the manuscript (include quotes in quotation marks "like this" to indicate direct quotes from your manuscript), or elaborate on this item by providing additional information not in the ms, or briefly explain why the item is not applicable/relevant for your study

Meine Antwort

14b) Why the trial ended or was stopped (early)

Does your paper address CONSORT subitem 14b? *

Copy and paste relevant sections from the manuscript (include quotes in quotation marks "like this" to indicate direct quotes from your manuscript), or elaborate on this item by providing additional information not in the ms, or briefly explain why the item is not applicable/relevant for your study

end of funding period for the project

15) A table showing baseline demographic and clinical characteristics for each group

NPT: When applicable, a description of care providers (case volume, qualification, expertise, etc.) and centers (volume) in each group

Does your paper address CONSORT subitem 15? *

Copy and paste relevant sections from the manuscript (include quotes in quotation marks "like this" to indicate direct quotes from your manuscript), or elaborate on this item by providing additional information not in the ms, or briefly explain why the item is not applicable/relevant for your study

"In total, 365 Arabic-speaking participants (CR: n=118; EXPO: n=122; WAIT: n=125) were included in the study. Participants were mainly female (n=272, 74.5%), single (n=227, 62.2%), living in urban areas (n=327, 89.6%), highly educated (n=331, 90.7%), and young adults (mean 25.49, SD 6.68; range 18 - 53). The largest share of participants originated from Egypt (26.3%), Saudi Arabia (18.9%), and Syria (12.6%), and were currently residing in Egypt (27.4%), Saudi Arabia (16.4%), and Jordan (6.8%). On average, participants reported 5.19 (SD 3.72) different traumatic events in the trauma exposure questionnaire, with the worst event most frequently involving sexual violence (i.e. 'sexual assault by family member or acquaintance' (n=69), 'sexual contact while under the age of 18 with a person at least 5 years older' (n=55), 'sexual assault by a stranger' (n=27)). PCL-5 scores at baseline ranged between 5 and 77, with a mean of 48.1 (SD 13.31). On average, participants reported an elevated level of depressive symptoms (mean 17.55, SD 5.17; range 2 - 27), anxiety symptoms (mean 14.21, SD 4.4; range 2 - 21), and somatoform symptoms (mean 14.5, SD 5.19; range 2 - 29). Besides suffering from PTSD, the majority of participants (n=268, 73.4%) suffered from a comorbid depressive disorder (current or past depressive episode, dysthymia, or both ("double depression")), as assessed using the SCID-5. An overview of sociodemographic, trauma-related, and clinical characteristics for participants in the total sample and in each condition is displayed in table 1 in the manuscript."

15-i) Report demographics associated with digital divide issues

In ehealth trials it is particularly important to report demographics associated with digital divide issues, such as age, education, gender, social-economic status, computer/Internet /ehealth literacy of the participants, if known.

	1	2	3	4	5	
subitem not at all important	○	○	○	◉	○	essential

Auswahl löschen

Does your paper address subitem 15-i? *

Copy and paste relevant sections from the manuscript (include quotes in quotation marks "like this" to indicate direct quotes from your manuscript), or elaborate on this item by providing additional information not in the ms, or briefly explain why the item is not applicable/relevant for your study

see table 1 in manuscript for sociodemographic information

16) For each group, number of participants (denominator) included in each analysis and whether the analysis was by original assigned groups

16-i) Report multiple "denominators" and provide definitions

Report multiple "denominators" and provide definitions: Report N's (and effect sizes) "across a range of study participation [and use] thresholds" [1], e.g., N exposed, N consented, N used more than x times, N used more than y weeks, N participants "used" the intervention/comparator at specific pre-defined time points of interest (in absolute and relative numbers per group). Always clearly define "use" of the intervention.

	1	2	3	4	5	
subitem not at all important	○	○	○	●	○	essential

Auswahl löschen

Does your paper address subitem 16-i? *

Copy and paste relevant sections from the manuscript (include quotes in quotation marks "like this" to indicate direct quotes from your manuscript), or elaborate on this item by providing additional information not in the ms, or briefly explain why the item is not applicable/relevant for your study

Figure 1 in the manuscript provides the flow of participants through the trial.

16-ii) Primary analysis should be intent-to-treat

Primary analysis should be intent-to-treat, secondary analyses could include comparing only “users”, with the appropriate caveats that this is no longer a randomized sample (see 18-i).

	1	2	3	4	5	
subitem not at all important	○	○	○	◉	○	essential

Auswahl löschen

Does your paper address subitem 16-ii?

Copy and paste relevant sections from the manuscript (include quotes in quotation marks "like this" to indicate direct quotes from your manuscript), or elaborate on this item by providing additional information not in the ms, or briefly explain why the item is not applicable/relevant for your study

Meine Antwort

17a) For each primary and secondary outcome, results for each group, and the estimated effect size and its precision (such as 95% confidence interval)

Does your paper address CONSORT subitem 17a? *

Copy and paste relevant sections from the manuscript (include quotes in quotation marks "like this" to indicate direct quotes from your manuscript), or elaborate on this item by providing additional information not in the ms, or briefly explain why the item is not applicable/relevant for your study

Main results (including effect sizes and CI) for the ITT are reported in table 3 to table 6 of the manuscript. Main results (including effect sizes and CI) for the completer sampe are reported in the suplementary material of the manuscript.

17a-i) Presentation of process outcomes such as metrics of use and intensity of use

In addition to primary/secondary (clinical) outcomes, the presentation of process outcomes such as metrics of use and intensity of use (dose, exposure) and their operational definitions is critical. This does not only refer to metrics of attrition (13-b) (often a binary variable), but also to more continuous exposure metrics such as "average session length". These must be accompanied by a technical description how a metric like a "session" is defined (e.g., timeout after idle time) [1] (report under item 6a).

	1	2	3	4	5	
subitem not at all important	○	○	◉	○	○	essential

Auswahl löschen

Does your paper address subitem 17a-i?

Copy and paste relevant sections from the manuscript (include quotes in quotation marks "like this" to indicate direct quotes from your manuscript), or elaborate on this item by providing additional information not in the ms, or briefly explain why the item is not applicable/relevant for your study

"Duration of treatment
Participants who started the CR condition (n=102) were in treatment for an average of 38.8 days (SD 19.7 days). Participants who started the EXPO condition (n=98) were in treatment for an average of 38 days (SD 18.4 days). For participants who completed the CR condition (n=64), mean treatment duration was 35.2 days (SD 15 days). For participants who completed the EXPO condition (n=59), mean treatment duration was 32.2 days (SD 13.3 days). The duration of treatment did not differ between both treatment conditions, neither regarding participants who started one of the treatments (U=4882, P=.78) nor regarding participants who completed one of the treatments (U=1652, P=.23).

Treatment satisfaction
Of all completers who answered the evaluation questions, 89.8 % (n=53) in the CR condition and 86.4% (n=51) in the EXPO condition were completely satisfied, very satisfied or satisfied with the treatment (U=1867, P=.48). Moreover, 93.2% (n=55) in the CR condition and 91.5% (n=54) in the EXPO condition experienced the treatment as very helpful, helpful or rather helpful (U=1920, P=.31). In both conditions, 94.9% of participants (n=56) would recommend the treatment to someone else (U=1787.5, P=.78). In terms of treatment duration, 57.6% (n=34) in the CR condition and 64.4% (n=38) in the EXPO condition experienced the treatment duration as sufficient (CR: n=0 too long, n=25 too short; EXPO: n=1 too long, n=20 too short; P=.45). "

17b) For binary outcomes, presentation of both absolute and relative effect sizes is recommended

Does your paper address CONSORT subitem 17b? *

Copy and paste relevant sections from the manuscript (include quotes in quotation marks "like this" to indicate direct quotes from your manuscript), or elaborate on this item by providing additional information not in the ms, or briefly explain why the item is not applicable/relevant for your study

"Overall, 40 participants (16.7%) did not start one of the treatments (CR: n=16, 13.6%; EXPO: n=24, 19.7%) after allocation to treatment. There was no significant association between treatment condition and the proportion of participants who did not start treatment vs. those who wrote at least one letter, $\chi 2=1.6$, P=.20. Concerning trauma-related and clinical characteristics, none of the comparisons between non-starters and starters reached significance (all P>.05). Regarding sociodemographic characteristics, starters included a significantly higher proportion of females (79%) than did non-starters (60%).
Of the n=200 participants who began one of the treatments, 123 (61.5%) completed all six letters (CR: n=64, 62.7%; EXPO: n=59, 60.2%). There was no significant association between treatment condition and the proportion of participants who completed the treatment vs. participants who dropped out, $\chi 2=0.1$, P=.71. The Welch tests revealed significant differences between completers and dropouts in both treatment conditions regarding baseline scores of overall posttraumatic stress symptom severity, negative alterations in cognition and mood, maladaptive posttraumatic beliefs, depressive symptom severity, and quality of life. Other comparisons were non-significant (all P>.05). The Games-Howell post hoc tests indicated that compared to participants who dropped out of the CR condition, those who completed the CR condition had lower baseline posttraumatic stress symptom severity, with a mean difference of 6.93 (95% CI [0.90, 12.95], P=.02), negative alterations in cognition and mood, with a mean difference of 2.69 (95% CI [0.33, 5.06], P=.02), and maladaptive posttraumatic beliefs, with a mean difference of 8.39 (95% CI [0.59, 16.20], P=.03). Furthermore, compared to participants who dropped out of the CR condition, those who completed either the EXPO or the CR condition had lower depressive symptom severity at baseline, with a mean difference of 2.97 (95% CI [0.47, 5.48], P=.01) and 2.71, (95% CI [0.20, 5.22], P=.03), respectively. Quality of life at baseline was significantly higher in participants who completed the CR condition compared to those who dropped out of the CR condition, with a mean difference of 2.50 (95% CI [0.20, 4.80], P=.03). Table 2 summarizes the characteristics of participants who dropped out and participants who completed one of the treatments as well as statistical results."

"Table 5 summarizes rates of reliable change, remission, and RCSI in all three conditions in the ITT sample. The two treatment conditions did not significantly differ regarding the rates of participants who experienced reliable change (P=.74), remission (P=.49), or RCSI (P=.52) between baseline and post-assessment. Rates of reliable change (P<.001), remission (P<.001), and RCSI (P<.001) differed significantly between the CR and the WAIT condition. Similarly, the EXPO and the WAIT condition differed significantly with regard to rates of reliable change (P<.001), remission (P<.001), and RCSI (P<.001)."

"Table S4 summarizes rates of reliable change, remission, and RCSI in all three conditions in the completer sample. The two treatment conditions did not significantly differ regarding the rates of participants who experienced reliable change (P=.87), remission (P=.57), or RCSI (P=.81) between baseline and post-assessment. Rates of reliable change (P<.001), remission (P<.001), and RCSI (P<.001) differed significantly between the CR and the WAIT condition. Similarly, the EXPO and the WAIT condition differed significantly with regard to rates of reliable change (P<.001), remission (P<.001), and RCSI (P<.001)."

18) Results of any other analyses performed, including subgroup analyses and adjusted analyses, distinguishing pre-specified from exploratory

Does your paper address CONSORT subitem 18? *

Copy and paste relevant sections from the manuscript (include quotes in quotation marks "like this" to indicate direct quotes from your manuscript), or elaborate on this item by providing additional information not in the ms, or briefly explain why the item is not applicable/relevant for your study

Results of the sensitivity analysis can be found in the supplementary material of the manuscript.

18-i) Subgroup analysis of comparing only users

A subgroup analysis of comparing only users is not uncommon in ehealth trials, but if done, it must be stressed that this is a self-selected sample and no longer an unbiased sample from a randomized trial (see 16-iii).

	1	2	3	4	5	
subitem not at all important	○	◉	○	○	○	essential

Auswahl löschen

Does your paper address subitem 18-i?

Copy and paste relevant sections from the manuscript (include quotes in quotation marks "like this" to indicate direct quotes from your manuscript), or elaborate on this item by providing additional information not in the ms, or briefly explain why the item is not applicable/relevant for your study

Meine Antwort

19) All important harms or unintended effects in each group
(for specific guidance see CONSORT for harms)

Does your paper address CONSORT subitem 19? *

Copy and paste relevant sections from the manuscript (include quotes in quotation marks "like this" to indicate direct quotes from your manuscript), or elaborate on this item by providing additional information not in the ms, or briefly explain why the item is not applicable/relevant for your study

Support for participants was provided via mail or phone in emergency situations (i.e., in case of suicidality, dropout). We are not aware of any harms caused by the intervention.

19-i) Include privacy breaches, technical problems

Include privacy breaches, technical problems. This does not only include physical “harm” to participants, but also incidents such as perceived or real privacy breaches [1], technical problems, and other unexpected/unintended incidents. “Unintended effects” also includes unintended positive effects [2].

	1	2	3	4	5	
subitem not at all important	○	◉	○	○	○	essential

Auswahl löschen

Does your paper address subitem 19-i?

Copy and paste relevant sections from the manuscript (include quotes in quotation marks "like this" to indicate direct quotes from your manuscript), or elaborate on this item by providing additional information not in the ms, or briefly explain why the item is not applicable/relevant for your study

Meine Antwort

19-ii) Include qualitative feedback from participants or observations from staff/researchers

Include qualitative feedback from participants or observations from staff/researchers, if available, on strengths and shortcomings of the application, especially if they point to unintended/unexpected effects or uses. This includes (if available) reasons for why people did or did not use the application as intended by the developers.

	1	2	3	4	5	
subitem not at all important	○	○	○	○	◉	essential

Auswahl löschen

Does your paper address subitem 19-ii?

Copy and paste relevant sections from the manuscript (include quotes in quotation marks "like this" to indicate direct quotes from your manuscript), or elaborate on this item by providing additional information not in the ms, or briefly explain why the item is not applicable/relevant for your study

Meine Antwort

DISCUSSION

22) Interpretation consistent with results, balancing benefits and harms, and considering other relevant evidence

NPT: In addition, take into account the choice of the comparator, lack of or partial blinding, and unequal expertise of care providers or centers in each group

22-i) Restate study questions and summarize the answers suggested by the data, starting with primary outcomes and process outcomes (use)

Restate study questions and summarize the answers suggested by the data, starting with primary outcomes and process outcomes (use).

	1	2	3	4	5	
subitem not at all important	○	○	○	◉	○	essential

Auswahl löschen

Does your paper address subitem 22-i? *

Copy and paste relevant sections from the manuscript (include quotes in quotation marks "like this" to indicate direct quotes from your manuscript), or elaborate on this item by providing additional information not in the ms, or briefly explain why the item is not applicable/relevant for your study

"The present study aimed to evaluate an internet-based cognitive restructuring treatment and an exposure treatment for Arabic-speaking participants with PTSD in terms of efficacy and course of PTSD symptom severity during treatment. First, we sought to examine the association of the two treatment conditions with treatment use. For this purpose, we investigated the proportion of participants who started treatment and the proportion who dropped out during treatment in both treatment conditions. Overall, 16.7% of participants did not start treatment (...). The proportion of participants who dropped out after starting treatment was 38.5% (...). There was no significant association between treatment condition and the proportion of participants who did not start treatment vs. those who wrote at least one letter, or between dropouts and completers or the duration of treatment. These findings demonstrate that the two treatment conditions were comparable with regard to use of the treatments. (...). Notably, some differences between completers and dropouts emerged in the current study, mainly in the CR condition: Participants who completed CR were less impaired by general posttraumatic stress and depressive symptoms and reported lower levels of negative alterations in cognition and mood and posttraumatic maladaptive beliefs. (...). Furthermore, treatment satisfaction of completers was examined and compared between the two treatments. In the present study, those participants who completed one of the treatments seemed to be satisfied with the treatment and experienced it as helpful, highlighting that a good working alliance, which forms the basis for therapeutic processes, can be established in highly standardized internet-based treatments in Arabic-speaking populations. However, in both conditions, nearly half of completers reported that they experienced the treatment as too short. (...) Finally, we examined changes in posttraumatic stress symptom severity, posttraumatic cognitions, anxiety, depressive, and somatoform symptom severity, and quality of life between baseline and post-assessment and compared them between all three conditions. Consistent with previous research on internet-based trauma-focused interventions in Arabic-speaking people, both treatments resulted in significant changes in PTSD symptom severity between baseline and post-assessment, and performed better than the waitlist control group. Between baseline and post-assessment, we found large effects for overall PTSD symptom severity and for most symptom clusters within both treatment conditions. The majority of participants reliably improved during one of the treatment conditions, and approximately 40% of the ITT sample experienced remission. (...). Even though the mean duration of both treatment conditions was a little bit longer than intended (mean duration of approx. 30 days), the treatments were still much shorter than most cognitive behavioral treatments conducted in a face-to-face setting. (...) Interestingly, significant changes in PTSD symptom severity within both conditions mainly emerged after four letters, with no significant differences between the two conditions. (...) Differences in the magnitude of change between the exposure treatment and the waitlist control group, as well as between the cognitive restructuring treatment and the waitlist control group, were significant for the comparison between baseline and post-assessment. (...). Notably, some significant symptom changes between baseline and post-assessment were also observed in the waitlist control group, with re-experiencing symptoms declining significantly, leading to a significant decline in overall PTSD symptom severity, although effect sizes were small. (...). Nevertheless, a significant change in overall posttraumatic stress symptom severity between baseline and post-assessment could not be detected in the waitlist control group under MNAR conditions.
In line with previous research with Arabic-speaking people, changes in secondary outcomes between baseline and post-assessment were significant in both treatment conditions. In our

sample, the largest effects for within-group changes were found for depressive and anxiety symptom severity and the lowest for somatoform symptom severity and trauma-related guilt cognitions. As the two treatment conditions did not differ significantly in any of the secondary outcomes, they appear to be equally effective for the treatment of other comorbid mental health symptoms, posttraumatic cognitions, and quality of life aspects in Arabic-speaking populations. The treatment conditions outperformed the waitlist control group regarding most secondary outcomes, with mostly medium-sized effects."

22-ii) Highlight unanswered new questions, suggest future research

Highlight unanswered new questions, suggest future research.

	1	2	3	4	5	
subitem not at all important	○	○	○	○	◉	essential

Auswahl löschen

Does your paper address subitem 22-ii?

Copy and paste relevant sections from the manuscript (include quotes in quotation marks "like this" to indicate direct quotes from your manuscript), or elaborate on this item by providing additional information not in the ms, or briefly explain why the item is not applicable/relevant for your study

Meine Antwort

20) Trial limitations, addressing sources of potential bias, imprecision, and, if relevant, multiplicity of analyses

20-i) Typical limitations in ehealth trials

Typical limitations in ehealth trials: Participants in ehealth trials are rarely blinded. Ehealth trials often look at a multiplicity of outcomes, increasing risk for a Type I error. Discuss biases due to non-use of the intervention/usability issues, biases through informed consent procedures, unexpected events.

	1	2	3	4	5	
subitem not at all important	○	○	○	◉	○	essential

Auswahl löschen

Does your paper address subitem 20-i? *

Copy and paste relevant sections from the manuscript (include quotes in quotation marks "like this" to indicate direct quotes from your manuscript), or elaborate on this item by providing additional information not in the ms, or briefly explain why the item is not applicable/relevant for your study

"Some limitations need to be taken into account when interpreting the present findings. The sample represented a rather specific group from the Arabic-speaking population, as it mainly consisted of female, young, and well-educated participants living in metropolitan cities. It seems that young and well-educated people with a stable internet connection are more familiar with new media and are more comfortable in using the Internet as a medium to receive treatment. In line with this, research has shown that women are more likely to use the Internet for health purposes, and that youth and higher education seem to positively affect health-related internet use. Moreover, due to limited personnel resources, we were unable to assess the participants' clinical status at the end of treatment by means of a clinical interview. Thus, results on the efficacy of the intervention are based on self-report measures. Finally, due to the limited follow-up data, we did not study the long-term effect of the interventions. "

21) Generalisability (external validity, applicability) of the trial findings

NPT: External validity of the trial findings according to the intervention, comparators, patients, and care providers or centers involved in the trial

21-i) Generalizability to other populations

Generalizability to other populations: In particular, discuss generalizability to a general Internet population, outside of a RCT setting, and general patient population, including applicability of the study results for other organizations

	1	2	3	4	5	
subitem not at all important	○	○	○	◉	○	essential

Auswahl löschen

Does your paper address subitem 21-i?

Copy and paste relevant sections from the manuscript (include quotes in quotation marks "like this" to indicate direct quotes from your manuscript), or elaborate on this item by providing additional information not in the ms, or briefly explain why the item is not applicable/relevant for your study

Meine Antwort

21-ii) Discuss if there were elements in the RCT that would be different in a routine application setting

Discuss if there were elements in the RCT that would be different in a routine application setting (e.g., prompts/reminders, more human involvement, training sessions or other co-interventions) and what impact the omission of these elements could have on use, adoption, or outcomes if the intervention is applied outside of a RCT setting.

	1	2	3	4	5	
subitem not at all important	○	○	◉	○	○	essential

Auswahl löschen

Does your paper address subitem 21-ii?

Copy and paste relevant sections from the manuscript (include quotes in quotation marks "like this" to indicate direct quotes from your manuscript), or elaborate on this item by providing additional information not in the ms, or briefly explain why the item is not applicable/relevant for your study

Meine Antwort

OTHER INFORMATION

23) Registration number and name of trial registry

Does your paper address CONSORT subitem 23? *

Copy and paste relevant sections from the manuscript (include quotes in quotation marks "like this" to indicate direct quotes from your manuscript), or elaborate on this item by providing additional information not in the ms, or briefly explain why the item is not applicable/relevant for your study

German Clinical Trial Register DRKS00010245

24) Where the full trial protocol can be accessed, if available

Does your paper address CONSORT subitem 24? *

Cite a Multimedia Appendix, other reference, or copy and paste relevant sections from the manuscript (include quotes in quotation marks "like this" to indicate direct quotes from your manuscript), or elaborate on this item by providing additional information not in the ms, or briefly explain why the item is not applicable/relevant for your study

A trial protocol is not available

25) Sources of funding and other support (such as supply of drugs), role of funders

Does your paper address CONSORT subitem 25? *

Copy and paste relevant sections from the manuscript (include quotes in quotation marks "like this" to indicate direct quotes from your manuscript), or elaborate on this item by providing additional information not in the ms, or briefly explain why the item is not applicable/relevant for your study

The Ilajnafsy project was funded by Misereor, Germany

X27) Conflicts of Interest (not a CONSORT item)

X27-i) State the relation of the study team towards the system being evaluated

In addition to the usual declaration of interests (financial or otherwise), also state the relation of the study team towards the system being evaluated, i.e., state if the authors/evaluators are distinct from or identical with the developers/sponsors of the intervention.

	1	2	3	4	5	
subitem not at all important	○	○	○	◉	○	essential

Auswahl löschen

Does your paper address subitem X27-i?

Copy and paste relevant sections from the manuscript (include quotes in quotation marks "like this" to indicate direct quotes from your manuscript), or elaborate on this item by providing additional information not in the ms, or briefly explain why the item is not applicable/relevant for your study

Meine Antwort

About the CONSORT EHEALTH checklist

As a result of using this checklist, did you make changes in your manuscript? *

- ○ yes, major changes
- ○ yes, minor changes
- ◉ no

What were the most important changes you made as a result of using this checklist?

Meine Antwort

How much time did you spend on going through the checklist INCLUDING making changes in your manuscript *

approximately two to three hours

As a result of using this checklist, do you think your manuscript has improved? *

- ○ yes
- ◉ no
- ○ Sonstiges:

Would you like to become involved in the CONSORT EHEALTH group?

This would involve for example becoming involved in participating in a workshop and writing an "Explanation and Elaboration" document

- (x) yes
- ( ) no
- ( ) Sonstiges:

Auswahl löschen

Any other comments or questions on CONSORT EHEALTH

Meine Antwort

STOP - Save this form as PDF before you click submit

To generate a record that you filled in this form, we recommend to generate a PDF of this page (on a Mac, simply select "print" and then select "print as PDF") before you submit it.

When you submit your (revised) paper to JMIR, please upload the PDF as supplementary file.

Don't worry if some text in the textboxes is cut off, as we still have the complete information in our database. Thank you!

Final step: Click submit !

Click submit so we have your answers in our database!

Senden

Alle Eingaben löschen

Geben Sie niemals Passwörter über Google Formulare weiter.

Dieser Inhalt wurde nicht von Google erstellt und wird von Google auch nicht unterstützt. Missbrauch melden - Nutzungsbedingungen - Datenschutzerklärung

Google Formulare
